# Eight New Peptaibols from Sponge-Associated *Trichoderma atroviride*

**DOI:** 10.3390/md11124937

**Published:** 2013-12-11

**Authors:** Irina Panizel, Oded Yarden, Micha Ilan, Shmuel Carmeli

**Affiliations:** 1 Raymond and Beverly Sackler School of Chemistry and Faculty of Exact Sciences, Tel Aviv University, Tel Aviv 69978, Israel; E-Mail: irinakha@post.tau.ac.il; 2 Department of Zoology, George S. Wise Faculty of Life Sciences, Tel Aviv University, Tel Aviv 69978, Israel; E-Mail: Milan@post.tau.ac.il; 3 Department of Plant Pathology and Microbiology, The Robert H. Smith Faculty of Agriculture, Food and Environment, The Hebrew University of Jerusalem, Rehovot 76100, Israel; E-Mail: Oded.Yarden@mail.huji.ac.il

**Keywords:** peptaibols, trichorzianines, *Trichoderma atroviride*, *Axinella polypoides*, *Axinella verrucosa*

## Abstract

Eight new and four known peptaibols were isolated from a strain of the fungus, *Trichoderma atroviride* (NF16), which was cultured from an Axinellid sponge collected from the East Mediterranean coast of Israel. The structures of the pure compounds were determined using HRMS, MS/MS and one- and two-dimensional NMR measurements. The isolated compounds belong to the trichorzianines, a family of 19-residue linear hydrophobic peptides containing a high proportion of α-aminoisobutyric acid (Aib), an acetylated *N*-terminus and a *C-*terminal amino alcohol. These new peptaibols exhibited antimicrobial activity against environmental bacteria isolated from the Mediterranean coast of Israel.

## 1. Introduction

Peptaibols are linear peptides of 5–20 residues, characterized by an acetate or acyl group in the *N-*terminus, *C*-terminal amino alcohol, a high proportion of α-aminoisobutyric acid (Aib) and other dialkylamino acids, such as isovaline (Iva). Most peptaibols were isolated from *Trichoderma* spp. or other closely related genera of fungi [1,2]. Peptaibols are produced by nonribosomal peptide synthetases [3]. To date, more than 300 peptaibols have been described, and their properties are summarized in the Peptaibol Database [4]. Peptaibols exhibit antimicrobial activity against fungi and Gram-positive bacteria and have an important role in biocontrol by *Trichoderma* spp. They act synergistically with cell wall degrading enzymes to inhibit the growth of fungal pathogens [5]. Trichorzianines are a subgroup of 19-residue peptaibols, initially isolated from *Trichoderma harzianum* [6,7,8]. They have similar amino acids sequences, except for differences in residues 5 (Aib or Iva), 14 (Leu or Val), 16 (Ile or Leu), 18 (Gln or Glu) and 19 (Pheol or Trpol) (Table S1 in the supporting material). A study, in which a trichorzianine type A (neutral trichorzianines) mixture was analyzed by MS/MS, revealed that in addition to the above-mentioned variable residues, other residues might be changed. In position 2, Ala might be substituted with Gly, in position 3 Ala with Gly or Aib, in position 5 Aib with Iva or Val, in positions 7, 8 and 9 Aib with Ala and in position 14 Val or Ile or Leu with Ala [9]. Recent investigation of five marine-derived *T*. *atroviride* strains afforded a new class of 17 residue-long peptaibols along with 19-residue peptaibols [10]. Peptaibols initially isolated from *Trichoderma atroviride* are termed atroviridins [11] and neoatroviridins [12]. LC-MS/MS study of *T*. *atroviride* peptaibols [2,13] revealed trichorzianines isolated from *T*. *harzianum* [7,8], as well.

In the current research, eight new and four known trichorzianines were isolated from the extract of *T. atroviride* (strain NF16), which was cultivated from an Axinellid sponge, collected by SCUBA diving from the Mediterranean Sea near Akhziv, Israel, as a part of a study on the chemical ecology of sponge-associated fungi. In re-isolation and culture trials, *T. atroviride* was re-isolated from both *Axinella polypoides* and *A. verrucosa* specimens (from 75% to 90% of the specimens; *n* = 4 and *n* = 11, respectively).

## 2. Results and Discussion

The culture medium was treated with Amberlite XAD-7 HP, and the compounds absorbed to the resin were washed from it with acetone. The crude acetone extract was subject to a Sephadex LH-20 column, and the active fraction was repeatedly separated on a preparative reversed phase HPLC column. The twelve peptaibols isolated in this study vary in four of 19 positions: position 5 was occupied by Aib or Iva, position 9 by Ala or Aib, position 14 by Leu or Val and position 17 by Glu, Glu-OMe or Gln (Table 1).

### 2.1. Structure Elucidation of Trichorzianine 1938 (TA1938) (**1**)

**Figure 1:**
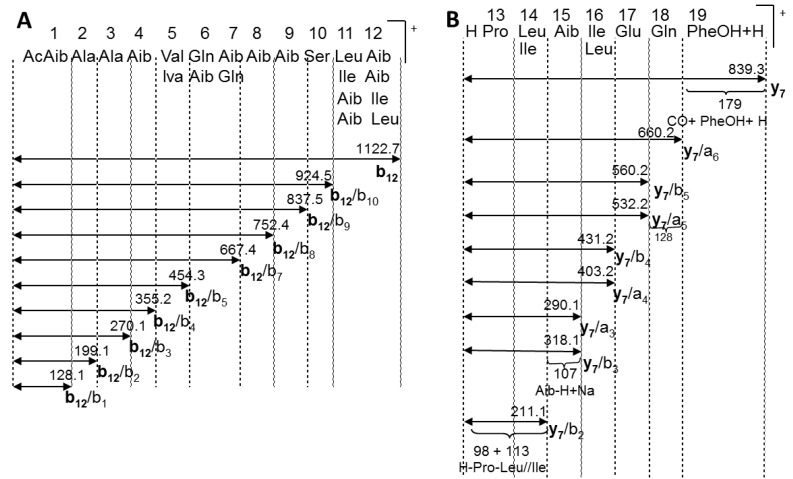
Secondary MS fragmentation of trichorzianine 1938 (**1**) ions at *m/z* 1122.5, **b_12_**-ion (**A**); and *m/z* 839.5, **y_7_**-ion (**B**).

Trichorzianine 1938 (TA1938) (**1**) was isolated as an amorphous white solid. In the ESI^+^ TOF MS of **1**, the quasi-molecular ions *m/z* 1939.1 ([M + H]^+^), *m/z* 1961.2 ([M + Na]^+^), *m/z* 981.1 ([M + H + Na]^2+^), *m/z* 992.0 ([M + 2Na]^2+^), and *m/z* 669.0 ([M + 3Na]^3+^) were observed. High-resolution mass measurement established its molecular formula, C_91_H_151_N_21_O_25_, for the [M + Na]^+^ ion of **1**, at *m/z* 1961.1099. In addition to the molecular ions, some prominent fragment ions were also present in the ESI^+^ spectrum; including the two complementary fragments, *m/z* 1122.7 and *m/z* 817.5, derived from the cleavage between the proline nitrogen and carboxyl group of the adjacent amino acid, characteristic of the peptaibols [14]. These two fragments were advantageous in the structural elucidation of **1** by MS/MS (Figure 1). 

**Table 1:** Trichorzianines (TA) **1**–**12** isolated from *Trichoderma atroviride* (NF16). Positions marked in grey differ between compounds. TA1938, trichorzianine 1938. Aib, α-amino-isobutyric acid.

Compound name	New/known	1	2	3	4	5	6	7	8	9	10	11	12	13	14	15	16	17	18	19
TA1938 (**1**)	**new**	AcAib	Ala	Ala	Aib	Iva	Gln	Aib	Aib	Aib	Ser	Leu	Aib	Pro	Leu	Aib	Ile	Glu	Gln	Pheol
TA1909 (**2**)	**new**	AcAib	Ala	Ala	Aib	Aib	Gln	Aib	Aib	Ala	Ser	Leu	Aib	Pro	Leu	Aib	Ile	Gln	Gln	Pheol
TA1895 (**3**)	**new**	AcAib	Ala	Ala	Aib	Aib	Gln	Aib	Aib	Ala	Ser	Leu	Aib	Pro	Val	Aib	Ile	Gln	Gln	Pheol
TA1896 (**4**)	**new**	AcAib	Ala	Ala	Aib	Aib	Gln	Aib	Aib	Ala	Ser	Leu	Aib	Pro	Val	Aib	Ile	Glu-OMe	Gln	Pheol
TA1924 (**5**)	**new**	AcAib	Ala	Ala	Aib	Iva	Gln	Aib	Aib	Aib	Ser	Leu	Aib	Pro	Val	Aib	Ile	Glu-OMe	Gln	Pheol
TA1910 (**6**)	**new**	AcAib	Ala	Ala	Aib	Iva	Gln	Aib	Aib	Ala	Ser	Leu	Aib	Pro	Val	Aib	Ile	Glu-OMe	Gln	Pheol
TA1924a (**7**)	**new**	AcAib	Ala	Ala	Aib	Aib	Gln	Aib	Aib	Aib	Ser	Leu	Aib	Pro	Leu	Aib	Ile	Glu-OMe	Gln	Pheol
TA1909a (**8**)	**new**	AcAib	Ala	Ala	Aib	Iva	Gln	Aib	Aib	Ala	Ser	Leu	Aib	Pro	Val	Aib	Ile	Gln	Gln	Pheol
TA.VIb (**9**)	known	AcAib	Ala	Ala	Aib	Aib	Gln	Aib	Aib	Aib	Ser	Leu	Aib	Pro	Val	Aib	Ile	Gln	Gln	Pheol
TA.VIa (**10**)	known	AcAib	Ala	Ala	Aib	Iva	Gln	Aib	Aib	Aib	Ser	Leu	Aib	Pro	Leu	Aib	Ile	Gln	Gln	Pheol
TA.VII (**11**)	known	AcAib	Ala	Ala	Aib	Iva	Gln	Aib	Aib	Aib	Ser	Leu	Aib	Pro	Val	Aib	Ile	Gln	Gln	Pheol
TA.Vb (**12**)	known	AcAib	Ala	Ala	Aib	Aib	Gln	Aib	Aib	Aib	Ser	Leu	Aib	Pro	Leu	Aib	Ile	Gln	Gln	Pheol

The ^1^H and ^13^C NMR spectra (Table 2 and Supplementary Figures S1 and S2) revealed that **1** is composed of the amino alcohol, phenylalaninol (Pheol), acetate, the non-proteinogenic amino acids, α-amino-isobutyric acid (Aib) and isovaline, and the proteinogenic amino acids, leucine, isoleucine, serine, glutamine, glutamic acid, proline and alanine. According to the MS/MS data (Figure 1), Aib appeared seven times in the molecule. The presence of the amino alcohol and isovaline and a high proportion of Aib indicated that the compound belonged to the peptaibol family. The amino acid sequence of the peptide, which was partially determined by the MS/MS, was confirmed and completed by analysis of the 2D NMR data (Table 2 and Supplementary Figures S3–S6). Initially, the structure of the amino acids were determined through interpretation of the COSY, TOCSY, HSQC and HMBC correlations, followed by their connection into the complete planar peptide structure through the HMBC and ROESY correlations and MS/MS data. 

**Table 2:** NMR data of trichorzianine 1938 ^a^ in DMSO-*d*_6_.

Position		δ_C_, mult. ^b^	δ_H_, mult., *J* (Hz)	LR C–H correlations ^c^	NOE correlations ^d^
Ac	1	171.1 s		Ac-2, ^1^Aib-NH,4	
	2	23.1 q	1.93 s		^1^Aib-NH,3,4
^1^Aib	1	175.7 ^e^ s		^2^Ala-NH, ^1^Aib-NH,3,4	
	2	55.8 s		^1^Aib-NH,3,4	
	3	26.2 ^g^ q	1.35 s	^1^Aib-NH	^1^Aib-NH
	4	24.0 ^h^ q	1.32 s	^1^Aib-3	^1^Aib-NH
	NH		8.56 s		Ac-2, ^1^Aib-3,4
^2^Ala	1	174.5 s		^3^Ala-NH, ^2^Ala-2,3	
	2	50.9 d	4.02 m	^2^Ala-NH,3	^2^Ala-NH,3
	3	16.8 q	1.31 d	^2^Ala-NH,2	^2^Ala-NH,2
	NH		8.25 d (5.2)		^3^Ala-NH, ^2^Ala-2,3
^3^Ala	1	174.5 s		^4^Aib-NH, ^3^Ala-2,3	
	2	51.0 d	3.99 m	^3^Ala-NH,3	^3^Ala-NH,3, ^4^Aib-NH
	3	16.2 q	1.33 d	^3^Ala-NH,2	^3^Ala-NH,2
	NH		7.68 d (5.6)		^4^Aib-NH, ^3^Ala-2,3, ^2^Ala-NH
^4^Aib	1	175.0 s		^5^Iva-NH	
	2	56.0 ^f^ s		^4^Aib-NH,3,4	
	3	26.1 ^g^ q	1.43 ^k^ s	^4^Aib-NH,4	^4^Aib-NH
	4	23.2 ^h^ q	1.35 ^l^ s	^4^Aib-NH,3	^4^Aib-NH
	NH		7.87 s		^4^Aib-2,3, ^3^Ala-NH,2
^5^Iva	1	176.4 s		^6^Gln-NH, ^5^Iva-3,NH	
	2	58.6 s		^5^Iva-NH,3a,3b,4,5	
	3a	25.6 t	2.20 m	^5^Iva-4	^5^Iva-4,3b
	3b		1.62 m		^5^Iva-4,3a
	4	7.4 q	0.74 t	^5^Iva-3a,3b	^5^Iva-NH,3a,3b
	5	22.7 q	1.35 ^l^ s	^5^Iva-NH,3a	^5^Iva-NH,
	NH		7.47 s		^5^Iva-4,5
^6^Gln	1	174.0 s		^7^Aib-NH, ^6^Gln-2	
	2	56.0 d	3.78 m	^6^Gln-NH,3,4a,4b	^7^Aib-NH, ^6^Gln-NH,3,4a,4b
	3	26.2 t	1.97 m	^6^Gln-2,4a,4b,NH	^6^Gln-2,4a,4b,NH, ^7^Aib-NH
	4a	31.6 ^i^ t	2.15 m	^6^Gln-3,NH_2_b	^6^Gln-2,3,4b,NH,NH_2_a
	4b		2.23 m		^6^Gln-2,3,4a
	5	173.6 s		^6^Gln-3,4a,4b,NH_2_a,b	
	NH_2_a		7.17 s		^6^Gln-4a,NH_2_b
	NH_2_b		6.76 s		^6^Gln-NH_2_a
	NH		7.74 m		^6^Gln-2,3,4a
^7^Aib	1	175.8 ^e^ s		^8^Aib-NH, ^7^Aib-NH,3,4	
	2	56.1 ^f^ s		^7^Aib-NH,3,4	
	3	25.8 ^g^ q	1.42 ^k^ s	^7^Aib-NH,4	^7^Aib-NH
	4	22.6 ^h^ q	1.34 s	^7^Aib-NH	^7^Aib-NH
	NH		7.86 s		^6^Gln-2,3, ^7^Aib-3,4, ^8^Aib-NH
^8^Aib	1	175.9 ^e^ s		^9^Aib-NH, ^8^Aib-3,4	
	2	56.1 ^f^ s		^8^Aib-NH,3,4	
	3	26.3 ^g^ q	1.42 ^k^ s	^8^Aib-NH,4	^8^Aib-NH
	4	23.3 ^h^ q	1.39 ^l^ s	^8^Aib-3	^8^Aib-NH
	NH		7.55		^8^Aib-3,4, ^7^Aib-NH, ^9^Aib-NH
^9^Aib	1	176.4 s		^10^Ser-NH, ^9^Aib-3,4	
	2	56.1 ^f^ s		^9^Aib-NH,3,4	
	3	26.5 ^g^ q	1.46 ^k^ s	^9^Aib-NH,4	
	4	23.0 ^h^ q	1.42 ^l^ s	^9^Aib-3	^9^Aib-NH
	NH		7.77		^9^Aib-4, ^8^Aib-NH
^10^Ser	1	170.6 s		^11^Leu-NH, ^10^Ser-2,3a	-
	2	58.9 d	4.02 m		^10^Ser-NH,3a,3b, ^11^Leu -NH
	3a	61.2 t	3.73 m	^10^Ser-NH,2	^10^Ser-NH,2
	3b		3.78 m		^10^Ser-NH,2
	OH				
	NH		7.74 m		^10^Ser-2,3a,3b
^11^Leu	1	173.7 ^j^ s		^12^Aib-NH, ^11^Leu-2	
	2	51.5 d	4.27 m	^11^Leu-NH,4	^12^Aib-NH, ^11^Leu-NH,3a,3b,5, ^13^Pro-5a
	3a	39.2 t	1.56 m	^11^Leu-2,5,6	^11^Leu-2,3b,5
	3b		1.70 m		^11^Leu-NH,2,3a,5
	4	23.9 d	1.73 m	^11^Leu-5,6	^11^Leu-NH,6
	5	20.5 q	0.76 d	^11^Leu-6,3b	^11^Leu-2,3a,3b
	6	23.0 q	0.82 d	^11^Leu-5,3b	^11^Leu-4
	NH		7.59 d (8.0)		^10^Ser-2, ^11^Leu-2,3b,4
^12^Aib	1	173.0 s		^12^Aib-NH,3,4	
	2	56.2 ^f^ s		^12^Aib-NH,3,4	
	3	25.8 ^g^ q	1.39 s	^12^Aib-NH	^12^Aib-NH
	4	23.2 ^h^ q	1.49 s	^12^Aib-NH	^12^Aib-NH, ^13^Pro-5b
	NH		7.89 s		^13^Pro-5a,5b, ^11^Leu-2, ^12^Aib-3,4
^13^Pro	1	173.4 s		^14^Leu-NH, ^13^Pro-2,3a	
	2	63.1 d	4.21 t (8.0)	^13^Pro-3a,4	^14^Leu-NH,5, ^13^Pro-3b,4
	3a	28.7 t	1.59 m	^13^Pro-2,5b	^13^Pro-3b,5a
	3b		2.22 m		^13^Pro-3a,2,4,5b
	4	26.2 t	1.86 m	^13^Pro-2	^13^Pro-2,3b,5a,5b
	5a	48.7 t	3.44 m	^13^Pro-3b	^13^Pro-2,3a,4,5b, ^11^Leu-2, ^14^Leu-NH, ^12^Aib-NH
	5b		3.69 m		^13^Pro-4,5a, ^12^Aib-NH,3
^14^Leu	1	173.7 ^j^ s		^15^Aib-NH, ^14^Leu-2	
	2	53.4 d	3.91 m	^14^Leu-NH,4	^14^Leu-3a,3b,4,5,6,NH, ^15^Aib-NH
	3a	38.7 t	1.51 m	^14^Leu-2,5,6	^14^Leu-2,3b,6,NH
	3b		1.78 m		^14^Leu-2,3a,6,NH
	4	24.8 d	1.68 m	^14^Leu-5,6	^14^Leu-2,5,NH
	5	23.0 q	0.92 d (6.0)	^14^Leu-6	^14^Leu-2,4, ^13^Pro-2
	6	21.0 q	0.82 m	^14^Leu-5	^14^Leu-2,3a,3b
	NH		7.72 m		^14^Leu-2,3a,3b,4, ^13^Pro-2,5a
^15^Aib	1	175.4 s		^16^Ile-NH, ^15^Aib-NH,3,4	
	2	56.3 ^f^ s		^15^Aib-NH,3,4	
	3	26.1 ^g^ q	1.44 ^k^ s	^15^Aib-4	^15^Aib -NH
	4	23.4 ^h^ q	1.37 ^l^ s	^15^Aib-NH,3	^15^Aib-NH
	NH		7.64 s		^16^Ile-NH, ^15^Aib-3,4, ^14^Leu-2
^16^Ile	1	172.0 s		^17^Glu-NH, ^16^Ile-2	-
	2	58.9 d	3.93 m	^16^Ile-NH,4b,6	^16^Ile-NH,3,4b,6
	3	35.6 d	1.88 m	^16^Ile-2,4a,4b,5,6	^16^Ile-NH,2,6
	4a	25.0 t	1.20 m	^16^Ile-2,3,5,6	^16^Ile-NH,4b
	4b		1.47 m		^16^Ile-NH,2,4a,5
	5	11.5 q	0.82 m	^16^Ile-4a	^16^Ile-4b
	6	15.6 q	0.86 d	^16^Ile-4a,2	^16^Ile-NH,2,3
	NH		6.96 s		^16^Ile-2,3,4a,4b,6, ^15^Aib-NH
^17^Glu	1	171.3 s		^18^Gln-NH, ^17^Glu-2,3a,3b	
	2	53.6 d	4.06 m	^17^Glu-NH,4a,4b	^18^Gln-NH, ^17^Glu-NH,3a,3b,4a,4b
	3a	26.5 t	1.87 m	^17^Glu-2,4a,4b	^17^Glu-NH,2,4b
	3b		1.97 m		^17^Glu-NH,2.4a
	4a	30.4 t	2.25 m	^17^Glu-2,3a,3b	^17^Glu-NH,2,3b
	4b		2.37 m		^17^Glu-NH,2,3a
	5	173.8 ^j^ s		^17^Glu-4b	
	NH		7.68 d (5.6)		^17^Glu-2,3a,3b,4a,4b
^18^Gln	1	170.8 s		^19^PheOH-NH, ^18^Gln-2,3a,3b	
	2	53.1 d	4.06 m	^18^Gln-NH,3a,3b,4a,4b	^19^PheOH-NH, ^18^Gln-NH,3a,3b,,4b
	3a	27.7 d	1.83 m	^18^Gln-2,4a,4b,NH	^18^Gln-NH,2,3b,4a
	3b		1.70 m		^18^Gln-NH,2,3a,4b
	4a	31.7 ^i^ t	2.05 m	^18^Gln-NH_2_a,2,3a,3b	^18^Gln-3a
	4b		1.97 m		^18^Gln-NH,NH_2_b,2,3b
	5	173.6		^18^Gln-NH_2_a,b,3a,3b,4a,4b	
	NH_2_a		6.65 s		^18^Gln-NH_2_b
	NH_2_b		7.10 s		^18^Gln-NH_2_a,4b
	NH		7.46 d		^19^PheOH-NH, ^18^Gln-2,3a,3b,4b
^19^PheOH	1a	62.7 t	3.29 m	^19^PheOH-2,3a,3b	^19^PheOH-NH,2,3a,3b,5
	1b		3.32 m		^19^PheOH-NH,2,3a,3b
	2	52.5 d	3.86 m	^19^PheOH-1a,1b,3a,3b,NH	^19^PheOH-NH,1a,1b,3a,3b,5
	3a	36.6 t	2.61 dd (13.4, 8.2)	^19^PheOH-1a,1b,2,5	^19^PheOH-NH,1a,1b,2,5
	3b		2.84 dd (13.2,4.8)		^19^PheOH-1a,1b,2,5
	4	139.2 s		^19^PheOH-2,3a,3b,5	
	5	129.4 d	7.21 m	^19^PheOH-3a,3b,6,7	^19^PheOH-1a,2,3a,3b,7,NH
	6	128.2 d	7.19 m	^19^PheOH-5,7	
	7	126.0 d	7.12 m	^19^PheOH-5,6	^19^PheOH-5,NH
	OH				
	NH		7.27 d (8)		^18^Gln-NH,2, ^19^PheOH-1a,1b,2,3a,5,7

^a^ Carried out on an AVANCE-400 Bruker instrument; ^b^ multiplicity and assignment are from HSQC experiment; ^c^ values determined from HMBC experiment; ^n^*J*_CH_ = 8 Hz, recycle time 1 s; ^d^ selected NOE’s from a ROESY experiment; ^e,f,g,h,i,j,k,l^ these signals may interchange.

According to the MS/MS spectra (Figure 1), the difference of 85 mass units appears seven times, in the positions 1, 4, 7 (or 6), 8, 9, 12 (or 11) and 15. The difference of 213 mass units in positions 6–7, which was explained by a couple of amino acids, glutamine (128) and Aib (85), could also be explained by the amino acids, valine or isovaline (99) and asparagine (114). The difference of 198 mass units in positions 11–12, which was explained by the amino acids, leucine (123) and Aib (85), could also be explained by the presence of two Vxx (valine or isovaline). However, according to the NMR data, valine and asparagine were not present in the molecule, and the only isovaline was assigned to position 5 according to the MS/MS and NMR data analysis. In the NMR spectra, signals typical of α-amino-isobutyric acid (Aib) were observed, including eight singlet amide signals resonating at δ_H_ 7.47 (assigned to the isovaline), 7.89, 8.56, 7.55, 7.64, 7.77, 7.86 and 7.87 ppm, multiple carbon signals resonating at δ_C_ 55.8–56.3, typical of carbons α to carboxyl; all of them, except one, (δ_C_ 56.0, assigned to ^6^glutamine) are quaternary according to the HSQC spectrum and multiple overlapping singlet methyl signals in the region of 1.30–1.49 ppm of the ^1^H NMR spectrum. According to the HSQC map, some of the protons of these methyl signals were bound to carbons resonating between 22.6 and 23.4 or between 25.8 and 26.7 ppm, while one to a carbon at 24.2 ppm. In addition to the Aib methyls, a proton signal of a single methyl of isovaline, one of the methylene protons of the isoleucine and methyl signals of ^2^alanine and ^3^alanine, resonate in the region of 1.30–1.49 ppm. Four methyl carbons, resonating between 22.6 and 23.4 ppm, were assigned to ^11^leucine, ^14^leucine, acetate and ^5^isovaline, in addition to the seven Aib methyls. In the region of 25.8–26.7 ppm, three methylene carbons of ^13^proline, ^6^glutamine and ^17^glutamic acid resonate beside the Aib methyl groups. Due to signal overlap, some geminal methyl groups and carbon α to carboxyls of Aib could not be distinguished unambiguously and remain interchangeable. Carboxyamides and amide protons were assigned according to HMBC correlations with amide protons and carboxyamide carbons of the neighboring amino acids, when the amino acids were connected to the full peptide chain. 

The structure determination of acetyl-^1^Aib started with the methyl protons (δ_H_ 1.93, s, δ_C_ 23.1) that exhibited an HMBC correlation with the carboxyamide (δ_C_ 171.1), which, in turn, was connected to a singlet amide proton resonating at δ_H_ 8.56. The amide proton and two singlet methyl groups (δ_H_ 1.32 and 1.35) exhibited HMBC correlations with a carboxyamide (one of the three carbons resonating at δ_C_ 175.7–175.9) and to a quaternary carbon α to a carboxyamide, resonating at δ_C_ 55.8), assigning the amino acid as ^1^Aib. The amide proton exhibited ROESY correlations to the methyl signals (δ_H_ 1.35, δ_C_ 26.5 and δ_H_ 1.32, δ_C_ 24.1). The carbon of the second methyl group exhibited an HMBC correlation with the protons of the first methyl (δ_H_ 1.35). These correlations allowed the assignment of the acetyl-^1^Aib sub-structure.

The assignment of the structure of ^2^alanine and ^3^alanine started with COSY correlations of the protons of two doublet methyl groups (δ_H_ 1.31, δ_H_ 1.33) with the α-protons resonating at δ_H_ 4.02 and 3.99, respectively, which, in turn, were connected to amide protons resonating at 8.25 and 7.68 ppm, respectively. This structure was reinforced by HMBC correlations (see Table 2). HMBC correlations connected the methyl groups and α-protons (δ_H_ 1.31, 1.33, 4.02, 3.99) to carboxyamide carbons resonating at δ_C_ 174.5, culminating in the structure of the two alanine residues.

A singlet amide proton resonating at δ_H_ 7.87 exhibited an HMBC correlation with the quaternary carbon resonating at δ_C_ 56.0 ppm and ROESY and HMBC correlations with two singlet methyl residues (δ_H_ 1.35, δ_C_ 23.2 and δ_H_ 1.43, δ_C_ 26.1), assigning the signals, except for the carboxyamide carbon of ^4^Aib. The other Aib residues in positions 7, 8, 9, 12 and 15 were assigned in a similar way.

The methyl protons (δ_H_ 0.74 t, δ_C_ 7.4) exhibited COSY correlation to methylene protons (δ_H_ 2.20 and 1.62) and HMBC correlation to the methylene carbon (δ_C_ 25.6). HMBC correlations of a quaternary carbon at δ_C_ 58.6, typical of a carbon α to carboxyamide, with the methyl and the methylene protons, established it as the isovaline C-2 carbon, which, in turn, was coupled by HMBC correlations to an amide proton (δ_H_ 7.47) and with a single methyl group (δ_H_ 1.35). Several singlet methyl groups, most belonging to Aib residues, resonated at 1.35 ppm, interfering with the assignment of this methyl carbon by HSQC. However, this carbon was assigned by HMBC correlation of the amide proton (δ_H_ 7.47) with a carbon resonating at 22.7 ppm. HMBC correlations of the amide (δ_H_ 7.47), one of the methylene protons (δ_H_ 2.20), and amide proton of the neighboring amino acid, ^6^glutamine with a carboxyamide carbon resonating at δ_C_ 174.6 ppm, established it as the ^5^isovaline carboxyamide. 

The structure determination of ^6^glutamine and ^18^glutamine was initiated through COSY correlations of the two pairs of singlet amide protons resonating at 7.17 and 6.76 ppm and at 7.10 and 6.65 ppm, respectively. These amide protons exhibited HMBC correlations to two carbonyl signals resonating at δ_C_ 173.6, thus establishing two primary amides. These amides were connected through HMBC correlations of the amide protons resonating at 6.65 ppm and 6.76 ppm to two methylene carbons resonating at 31.7–31.6 ppm (^6^C-4 and ^18^C-4, δ_H_ 2.15, 2.23 and 2.05, 1.97, respectively), which, in turn, exhibited HMBC correlations with two pairs of methylene protons, (^6^Gln-3, δ_H_ 1.97, 2H, δ_C_ 26.2) and (^18^Gln-3, δ_H_ 1.70, 1.83 ppm, δ_C_ 27.7). One of the C-3 carbons (26.2 ppm) exhibited HMBC correlations to the pair of methylene-4 protons resonating at 2.15 and 2.23 ppm, while the second C-3 carbon (27.7 ppm) exhibited HMBC correlation with the methylene protons at 1.97 ppm and 2.05 ppm (H-4a, H-4b), allowing the differentiation between the two spin systems. HMBC correlations of C-3 carbons at 26.2 and 27.7 ppm with the methine protons resonating at δ_H_ 3.78 (δ_C_ 56.0) and δ_H_ 4.06 (δ_C_ 53.1), respectively, established the methines at position 2 of these amino acids. This assignment was further supported by the COSY correlations of H_2_-3 with H-2 of both spin systems. The α-amide protons were assigned through the COSY correlation of the α-proton, resonating at 3.78 ppm with the amide proton resonating at 7.74 ppm, and that at 4.06 ppm with that at 7.47 ppm, and reinforced by the HMBC correlations of C-2 carbons with these amide protons. HMBC correlations of the carbon resonating at 174.0 ppm with the proton resonating at 3.78 ppm, and of the carbon resonating at 170.8 ppm with protons resonating at 4.06 ppm, 1.70 ppm and 1.83 ppm, established those carbons as the carboxyamides of the glutamine residues.

The structure elucidation of ^10^serine was based on COSY correlations of the oxymethylene protons (δ_H_ 3.73, 3.78; δ_C_ 61.2) with a proton resonating at 4.02 ppm (δ_C_ 58.9), which, in turn, was connected to an amide proton resonating at δ_H_ 7.74. HMBC correlations of the protons resonating at δ_H_ 4.02 and 3.73 with the carbon resonating at δ_C_ 170.6 established the latter as a serine carboxyamide. The hydroxyl proton was not observed in the NMR spectrum of TA1938, but was observed at chemical shifts of 4.70–4.85 ppm in some of the other peptaibols (TA1895, TA1909, TA1909A, TA1896 and TA.Vb). 

Two methyl groups, H_3_-5 and H_3_-6 (δ_H_ 0.76 d, 0.82 d), were coupled to the same methine proton (H-4, δ_H_ 1.73, C-4: δ_C_ 23.9) through COSY correlations. In the HMBC spectrum, the two methyl groups exhibit correlations to C-4 (23.9 ppm) and to a methylene carbon (δ_C_ 39.2, C-3, δ_H_ 1.56 and 1.70). COSY correlations coupled H-3b and H-3a through H-2 (δ_H_ 4.27) to an amide resonating at δ_H_ 7.59 and HMBC correlation to one of the three carbons resonating at δ_C_ 173.7–173.8, establishing the structure of ^11^leucine. 

The structure elucidation of the ^13^proline was initiated with COSY correlation of the aminomethylene protons resonating at δ_H_ 3.44 and 3.69 (H-5a and H-5b) with methylene protons resonating at δ_H_ 1.86 (H-4a and -4b). The later protons were coupled through COSY correlations to protons of additional methylene δ_H_ 1.59 and 2.22 (H-3a and H-3b), which, in turn, exhibited COSY correlations with a methine proton (δ_H_ 4.21, t). The chemical shifts of this proton and the carbon to which it was attached (δ_C_ 63.1, established through HSQC experiment) indicated their location α to a carboxyamide and amine. HMBC correlations between the methine carbon (δ_C_ 63.1 d, C-2) and H-3b (δ_H_ 2.22), C-3 (δ_C_ 28.7, CH_2_) and H-2, C-5 (δ_C_ 48.7) and H-3b reinforced this structure. H-2 (δ_H_ 4.21) was connected through HMBC correlation to a carbon that resonated at δ_C_ 173.4, which was assigned as C-1 of this residue. No HMBC or ROESY correlations were present, which could prove the ring closure. However, the chemical shifts of the H-5a, H-5b and C-5 (δ_H_ 3.44 and 3.69, δ_C_ 48.7) indicated their vicinity to a nitrogen atom. Besides, none of the carbons or protons of this residue exhibited correlations with any of the amide protons, indicating a tertiary amide. In addition, the chemical shifts of the carbons and protons were similar to those of proline in TA1909 (**2**), in which a ^3^*J* HMBC correlation between C-2 (δ_C_ 63.0) and H-5b (δ_H_ 3.71) was observed, indicating the closure of a pyrrolidine ring. Based on the above evidences, the structure of this amino acid was established as proline. 

The structure elucidation of ^14^leucine started with COSY correlations of two methyl groups resonating at δ_H_ 0.92 and 0.82 (H_3_-5 and H_3_-6, respectively) with the same methine proton that resonated at δ_H_ 1.68 (H-4). In the HMBC spectrum, H_3_-5 and -6 exhibited correlations with a methine carbon (δ_C_ 24.8, C-4) and a methylene carbon, (δ_C_ 38.7, C-3, δ_H_ 1.51 and 1.78, H-3a and H-3b, respectively). H-3a and H-3b exhibited COSY correlations with a proton resonating at δ_H_ 3.91 (H-2), which, in turn, was correlated with the amide proton resonating at δ_H_ 7.72. HMBC correlation connected H-2 and one of three carbons resonating at δ_C_ 173.7–173.8. This HMBC correlation was weak, but the connectivity was reinforced by the HMBC correlation of this carbon with the amide proton δ_H_ 7.64 of the adjacent amino acid, ^15^Aib.

The structure elucidation of ^16^isoleucine started with COSY correlation of protons of a doublet methyl, (H_3_-6, resonating at δ_H_ 0.86, δ_C_ 15.7) with a methine proton, H-3 (δ_H_ 1.88). Methine-3 carbon (δ_C_ 35.6) exhibited HMBC correlations with the protons of CH_3_-6, the protons of an additional triplet methyl group (δ_H_ 0.82) and protons of a methylene (δ_H_ 1.20 and 1.47, δ_C_ 25.0 by HSQC). COSY correlations of the methylene protons with the methyl protons at δ_H_ 0.82 determined the ethyl segment. COSY correlations coupled the ethyl moiety through the methine proton at 1.88 ppm to a downfield shifted methine (H-2, δ_H_ 3.93, C-2, δ_C_ 58.9) and the later to an amide proton resonating at δ_H_ 6.96. H-2 presented an HMBC correlation to a carbon resonating at δ_C_ 170.6 that was assigned as the carboxyamide carbon of the isoleucine residue. In the ^13^C NMR spectrum, three carbons resonate at 173.7–173.8 ppm. Two of them were attributed to ^11^leucine and ^14^leucine. The third one exhibited an HMBC correlation to a proton that resonated at 2.37 ppm (H-4b, δ_C_ 30.4, δ_H-4a_ 2.25). The protons of the later methylene were coupled through COSY correlations to protons that resonated at δ_H_ 1.97 and 1.87 (H-3a and H-3b, δ_C_ 26.5), which, in turn, were connected to an α-proton resonating at 4.06 ppm and to an amide proton (δ_H_ 7.68). HMBC correlations of the carbon resonating at δ_C_ 171.3 with the protons at δ_H_ 4.06, 1.97 and 1.87 established it as the glutamic acid carboxamide, establishing the structure of ^17^glutamic acid. 

The four aromatic carbons presented in the ^13^C spectrum were assigned to ^19^phenylalaninol. The carbon at δ_C_ 139.2 was identified as a quaternary carbon and the other three carried protons (δ_C_ 126.0, δ_H_ 7.12), (δ_C_ 128.2, δ_H_ 7.18) and (δ_C_ 129.4, δ_H_ 7.21). The signal intensity of the carbons and integration of the protons indicated that two pairs of symmetric aromatic protons resonate at 7.18 and 7.21 ppm and one proton resonates at δ_H_ 7.12 ppm, in accordance with a mono-substituted phenyl ring. COSY correlations coupled the later proton with the protons resonating at δ_H_ 7.18 (H-6,6′, δ_C_ 128.2) and to those resonating at δ_H_ 7.21 (H-5,5′, δ_C_ 129.4). This was reinforced by HMBC correlations (see Table 2). The assignment of the aliphatic part of the amino alcohol was based on HMBC correlations of the aromatic carbons, C-4 and C-5, with the two methylene protons, δ_H_ 2.84 and 2.61 ppm (H-3a, H-3b), which were coupled by COSY correlations to a methine proton resonating at δ_H_ 3.86 (H-2). This methine proton was connected to an amide proton (δ_H_ 7.27 ppm) and to the protons of an oxymethylene resonating at δ_H_ 3.29 and 3.32 (H-1a and H-1b, δ_C_ 62.7). The hydroxyl proton of the phenylalaninol did not appear in the NMR spectra of **1**, but was present in other trichorzianine: TA1895, TA1909, TA1909A, TA1896 and TA.Vb.

**Figure 2:**
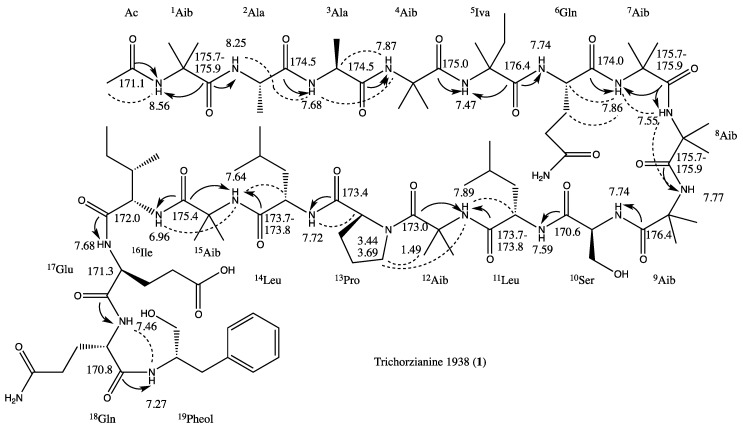
Structure of trichorzianine 1938 (**1**) with HMBC (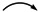) and ROESY (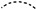) correlations.

The assembling of the amino acids to the planar peptide structure was based on HMBC correlations from the carboxyamide carbon of an amino acid to the amide protons of the adjacent amino acid, by NOE correlations (from ROESY experiment) of the α- or amide proton of an amino acid with the α- or amide proton of the adjacent amino acid and by interpretation of MS/MS data. The structure of the peptide with the most significant HMBC and NOE correlations that led to the structure elucidation are summarized in Figure 2. The sequence Ac-^1^Aib-^2^Ala-^3^Ala-^4^Aib-^5^Iva/Val was inferred from MS/MS data. ^1^Aib carboxyamide carbon resonated (δ_C_ 175.7–175.9) closely with those of ^7^Aib and ^8^Aib, excluding an unequivocal proof of the HMBC correlation of ^2^Ala-NH with ^1^Aib-C-1. 

The carboxyamides carbons of ^2^Ala and ^3^Ala resonated at the same chemical shift (δ_C_ 174.5), not allowing the confirmation of their connection by HMBC correlation, but NOE correlation of ^2^Ala-NH with ^3^Ala-NH confirmed their vicinity. NOE correlation of ^3^Ala-H-2 with ^4^Aib-NH and HMBC correlation of ^4^Aib-C-1 with ^5^Iva-NH established the ^3^Ala-^4^Aib-^5^Iva partial structure. The connection of ^5^Iva with ^6^Gln could not be proven either by HMBC or NOE correlations, since the carboxyamides of ^5^Iva and ^9^Aib (δ_C_ 174.6) and the doublet amide protons of ^6^Gln and ^1^^0^Ser (δ_H_ 7.74) resonated at the same chemical shifts. The correlation of these chemical shifts (δ_C_ 174.6 with δ_H_ 7.74) in the HMBC map remained unequivocal, while no correlations between ^5^Iva and ^6^Gln were observed in the ROESY map. The MS/MS data suggested that ^5^Iva was connected either to Gln or Aib. HMBC and especially NOE correlations (Table 2) confirmed the ^6^Gln-^7^Aib-^8^Aib-^9^Aib substructure, and in conjugation, with the MS/MS data, the ^5^Iva-^6^Gln-^7^Aib-^8^Aib-^9^Aib-^1^^0^Ser sequence could be secured. ^1^^0^Ser was connected to ^11^Leu based on the HMBC correlation of ^1^^0^Ser-carboxyamide with ^11^Leu-NH. ^11^Leu- and ^14^Leu- carboxyamides resonated close one to the other (δ_C_ 173.7–173.8), not allowing unambiguous assignment of their neighboring amino acid residues. However, NOE correlations of ^12^Aib-NH with ^11^Leu-NH and ^13^Pro-5-Ha and 5-Hb established the ^11^Leu-^12^Aib-^13^Pro sequence. HMBC correlation of ^13^Pro-C-1 with ^14^Leu-NH established the connectivity of the latter two amino acids. NOE correlations of ^15^Aib-NH with ^14^Leu-H-2 and ^16^Ile-NH determined the ^14^Leu-^15^Aib-^16^Ile fragment. The rest of the sequence, ^16^Ile-^17^Glu-^18^Gln-^19^Pheol, could be secured with correlations from the HMBC map, resulting in the full planar structure of **1**.

### 2.2. Structure Elucidation of **2**–**12**

The structures of compounds **2**–**12** were determined in a similar manner to that of TA1938 (**1**). Compounds **2**–**12** vary from **1** in the amino acid residues of positions 5, 9, 14 and 17. ^1^H- and ^13^C-NMR data of the new trichorzianines are summarized in Table 3 and Table 4. Schemes of the secondary fragmentation in the MS/MS (Supplementary Figures S7–S15), complete NMR data (Supplementary Tables S2–S12 and Figures S16–S29) and the assemblage of the amino acids to the planar peptide structures (Supplementary Figures S30–S40) of compounds **2**–**12** are displayed in the supplementary material. The presence of Glu-OMe in compounds **4**–**7** was supported by the direct connectivity of the OMe-proton signal with the side-chain carboxyl carbon of Glu.

**Table 3:** ^1^H NMR chemical shifts of trichorzianines **2**–**8** in DMSO-*d*_6_.

Position		TA1909 (2) ^a^	TA1895 (3) ^a^	TA1896 (4) ^b^	TA1924 (5) ^b^	TA1910 (6) ^a^	TA1924a (7) ^a^	TA1909a (8) ^a^
Ac	2	1.93 s	1.92 s	1.92 s	1.92 s	1.92 s	1.93 s	1.93 s
^1^Aib	3	1.35 s	1.35 s	1.35 s	1.35 s	1.35 s	1.36 s	1.36 s
	4	1.32 s	1.32 s	1.32 s	1.32 s	1.33 s	1.32 s	1.33 s
	NH	8.57 s	8.57 s	8.58 s	8.54 s	8.57 s	8.57 s	8.56 s
^2^Ala	2	3.99 m	3.98 m	3.98 m	4.00 m	4.01 m	4.01 m	4.01 m
	3	1.31 d	1.31 d	1.31 d	1.30 d	1.31 d	1.31 d	1.31 d
	NH	8.27 d	8.27 d	8.27 d	8.24 d	8.26 d	8.27 d	8.25 d
^3^Ala	2	3.99 m	3.99 m	3.98 m	4.02 m	4.01 m	4.01 m	4.01 m
	3	1.33 d	1.33 d	1.33 d	1.33 d	1.33 d	1.33 d	1.33 d
	NH	7.69 d	7.69 d	7.70 d	7.68 d	7.68 d	7.71 m	7.68 d
^4^Aib	3	1.41 ^d^ s	1.39 ^c^ s	1.43 ^c^ s	1.43 s	1.45 ^c^ s	1.45 s	1.46 s
	4	1.36 ^c^ s	1.38 s	1.38 ^d^ s	1.35 s	1.35 ^d^ s	1.38 ^c^ s	1.35 ^c^ s
	NH	7.81 s	7.81 s	7.82 s	7.86 s	7.87 s	7.83 s	7.87 s
^5^Iva/^5^Aib	3a	1.44 s	1.42 ^e^ s	1.43 ^c^ s	2.20 m	2.21 m	1.44 s	2.21 m
	3b				1.62 m	1.62 m		1.62 m
	4	1.39 s	1.37 ^d^ s	1.35 s	0.73 t	0.73 m	1.36 ^c^ s	0.73 t
	5				1.35 ^c^ s	1.35 s		1.36 s
	NH	7.54 s	7.53 s	7.53 s	7.46 s	7.47 s	7.55 s	7.47 s
^6^Gln	2	3.74 m	3.73 m	3.74 m	3.78 m	3.78 m	3.77 m	3.78 m
	3	1.98 m	1.99 m	1.99 m	1.95 m	1.97 m	1.97 m	1.97 m
	4a	2.13 m	2.13 m	2.13 m	2.14 m	2.14 m	2.15 m	2.14 m
	4b	2.23 m	2.23 m	2.23 m	2.23 m	2.23 m	2.23 m	2.23 m
	NH_2_a	7.13 s	7.13 s	7.17 s	7.16 s	7.16 s	7.16 s	7.16 s
	NH_2_b	6.75 s	6.75 s	6.75 s	6.74 s	6.77 s	6.76 s	6.76 s
	NH	7.74 m	7.74 m	7.74 m	7.72 m	7.73 m	7.77 m	7.73 m
^7^Aib	3	1.41 ^d^ s	1.41 ^c^ s	1.44 s	1.42 ^d^ s	1.43 ^c^ s	1.45 ^d^ s	1.43 ^d^ s
	4	1.34 ^c^ s	1.37 ^d^ s	1.39 ^d^ s	1.35 ^c^ s	1.36 ^d^ s	1.35 ^c^ s	1.36 ^c^ s
	NH	7.90 s	7.89 s	7.89 s	7.84 s	7.90 s	7.86 s	7.90 s
^8^Aib	3	1.46 ^c^ s	1.45 s	1.46 s	1.42 ^d^ s	1.45 ^c^ s	1.43 ^d^ s	1.43 ^d^ s
	4	1.38 ^d^ s	1.35 ^d^ s	1.39 ^d^ s	1.38 ^c^ s	1.35 ^d^ s	1.35 ^c^ s	1.39 ^c^ s
	NH	7.59	7.58 s	7.58 s	7.54 s	7.55 s	7.59 s	7.55 s
^9^Ala/^9^Aib	2	3.94 m	3.93 m	3.93 m		3.96 m		3.96 m
	3	1.40 d	1.40 d	1.40 d	1.46 s	1.40 d	1.48 s	1.40 d
	4				1.42 s		1.43 s	
	NH	7.73 m	7.73 m	7.73 m	7.75 s	7.72 s	7.79 s	7.72 s
^10^Ser	2	4.09 m	4.09 m	4.09 m	4.02 m	4.10 m	4.03 m	4.11 m
	3a	3.72 m	3.72 m	3.73 m	3.76 m	3.73 m	3.75 m	3.72 m
	3b	3.77 m	3.77 m	3.77 m	3.78 m	3.76 m	3.79 m	3.76
	OH	4.86 t	4.85 t	4.85 t	-	-	-	4.87 t
	NH	7.77 m	7.77 m	7.76 m	7.73 m	7.76 d	7.74 m	7.76 m
^11^Leu	2	4.27 m	4.27 m	4.27 m	4.28 m	4.28 m	4.27 m	4.28 m
	3a	1.54 m	1.52 m	1.53 m	1.55 m	1.55 m	1.56 m	1.55 m
	3b	1.67 m	1.65 m	1.66 m	1.68 m	1.67 m	1.70 m	1.67 m
	4	1.73 m	1.68 m	1.69 m	1.69 m	1.69 m	1.73 m	1.69 m
	5	0.76 d	0.77 d	0.77 m	0.76 d	0.78 d	0.76 m	0.78 d
	6	0.81 d	0.83 d	0.83 d	0.83 d	0.84 d	0.82 d	0.84 d
	NH	7.43 d	7.44 d	7.43 m	7.59 d	7.44 d	7.59 d	7.44 m
^12^Aib	3	1.40 s	1.38 ^e^ s	1.36 s	1.38 s	1.39 s	1.37 s	1.40 s
	4	1.46 s	1.45 s	1.47 s	1.48 s	1.46 s	1.49 s	1.49 s
	NH	7.78 s	7.91 s	7.89 s	7.93 s	7.91 s	7.92 s	7.93 s
^13^Pro	2	4.21 t	4.23 t	4.21 t	4.22 t	4.22 t	4.21 t	4.24 t
	3a	1.58 m	1.64 m	1.64 m	1.65 m	1.65 m	1.60 m	1.65 m
	3b	2.22 m	2.22 m	2.22 m	2.22 m	2.22 m	2.24 m	2.22 m
	4	1.84 m	1.84 m	1.84 m	1.86 m	1.86 m	1.86 m	1.86 m
	5a	3.38 m	3.47 m	3.47 m	3.52 m	3.48 m	3.45 m	3.48 m
	5b	3.71 m	3.69 m	3.69 m	3.67 m	3.70 m	3.70 m	3.70 m
^14^Leu/^14^Val	2	3.94 m	3.76 m	3.68 m	3.72 m	3.68 m	3.90 m	3.77 m
	3a	1.51 m	2.22 m	2.22 m	2.21 m	2.21 m	1.52 m	2.21 m
	3b	1.78 m					1.78 m	
	4	1.67 m	0.93 d	0.93 d	0.93 d	0.94 d	1.68 m	0.94 d
	5	0.91 d	0.86 d	0.86 d	0.87 d	0.89 d	0.92 d	0.88 d
	6	0.82 d					0.82 d	
	NH	7.74 m	7.59 m	7.59 m	7.58 m	7.59 d	7.72 m	7.60 d
^15^Aib	3	1.42 ^d^ s	1.43 s	1.42 ^c^ s	1.42 ^d^ s	1.43 ^c^ s	1.42 ^d^ s	1.45 ^d^ s
	4	1.35 ^c^ s	1.37 ^d^ s	1.35 s	1.35 ^c^ s	1.38 ^d^ s	1.36 ^c^ s	1.39 ^c^ s
	NH	7.63 s	7.48 s	7.45 s	7.43 s	7.43 s	7.63 s	7.43 s
^16^Ile	2	3.91 m	3.84 t	3.87 t	3.88 m	3.88 m	3.93 m	3.87 m
	3	1.87 m	1.88 m	1.88 m	1.88 m	1.88 m	1.88 m	1.88 m
	4a	1.20 m	1.20 m	1.20 m	1.20 m	1.20 m	1.20 m	1.20 m
	4b	1.47 m	1.47 m	1.47 m	1.50 m	1.50 m	1.47 m	1.50 m
	5	0.81 t	0.81 t	0.80 t	0.80 t	0.81 t	0.82 t	0.81 t
	6	0.85 d	0.85 d	0.85 d	0.84 d	0.85 d	0.86 d	0.86 d
	NH	6.96 d	7.22 m	7.20 m	7.20 m	7.21 m	6.94 d	7.22 m
^17^Gln/^17^Glu	2	4.03 m	3.95 m	4.01 m	4.01 m	4.02 m	4.06 m	3.97 m
	3a	1.85 m	1.90 m	1.98 m	1.95 m	1.95 m	1.87 m	1.90 m
	3b	1.94 m					1.97 m	
	4a	2.08 m	2.13 m	2.41 m	2.40 m	2.49 m	2.37 m	2.13 m
	4b	2.18 m	2.23 m	2.47 m	2.30 m	2.40 m	2.44 m	2.23 m
	NH	7.69 d	7.69 d	7.67 d	7.68 d	7.68 d	7.68 m	7.69 d
	OMe			3.55 s	3.55 s	3.55 s	3.54 s	
	NH_2_a	6.72 s	6.72 s					6.72 s
	NH_2_b	7.13 s	7.13 s					7.13 s
^18^Gln	2	4.04 m	4.00 m	4.01 m	4.03 m	4.03 m	4.06 m	4.03 m
	3a	1.83 m	1.81 m	1.83 m	1.81 m	1.83 m	1.83 m	1.85 m
	3b	1.70 m	1.70 m	1.70 m	1.70 m	1.72 m	1.70 m	1.72 m
	4a	2.03 m	2.04 m	2.05 m	2.05 m	2.05 m	2.05 m	2.05 m
	4b	1.97 m	1.97 m	1.97 m	1.97 m	1.98 m	1.97 m	1.98 m
	5							
	NH_2_a	6.64 s	6.62 s	6.51 s	6.61 s	6.63 s	6.65 s	6.63 s
	NH_2_b	7.10 s	7.07 s	7.05 s	7.04 s	7.07 s	7.10 s	7.07 s
	NH	7.47 d	7.44 d	7.45 d	7.44 d	7.46 d	7.48 d	7.46 d
^19^Pheol	1a	3.28 m	3.30 m	3.30 m	3.30 m	3.30 m	3.28 m	3.30 m
	1b	3.32 m	3.32 m	3.33 m	3.33 m	3.33 m	3.32 m	3.33 m
	2	3.86 m	3.84 t	3.86 m	3.86 m	3.86 m	3.87 m	3.86 m
	3a	2.61 dd	2.61 dd	2.61 dd	2.61 dd	2.62 dd	2.61 dd	2.63 dd
	3b	2.83 dd	2.83 dd	2.83 dd	2.84 dd	2.84 dd	2.84 dd	2.84 dd
	5	7.20 m	7.22 m	7.21 m	7.21 m	7.22 m	7.21 m	7.22 m
	6	7.19 m	7.18 m	7.19 m	7.19 m	7.20 m	7.19 m	7.20 m
	7	7.11 m	7.11 m	7.11 m	7.11 m	7.12 m	7.13 m	7.12 m
	OH	4.70 t	4.69 t	4.67 t				4.69 t
	NH	7.27 d	7.15 m	7.18 m	7.18 m	7.19 m	7.30 d	7.18 m

^a^ Instrument ^1^H frequency 400 MHz; ^b^ Instrument ^1^H frequency 500 MHz; ^c,d,e^ These signals may interchange in columns.

**Table 4:** ^13^C NMR data of new trichorzianines in DMSO-*d*_6_.

Position		TA1909 (2) ^a^	TA1895 (3) ^a^	TA1896 (4) ^b^	TA1924 (5) ^b^	TA1910 (6) ^a^	TA1924a (7) ^a^	TA1909a (8) ^a^
Ac	1	171.1 s	171.2 s	171.1s	171.0 s	171.1 s	171.1 s	171.1 s
	2	23.1 q	23.3 q	23.2 q	23.0 q	23.0 q	23.1 q	23.1 q
^1^Aib	1	176.0 ^e^ s	176.0 ^e^ s	175.9 ^e^ s	175.6 ^e^ s	175.8 s	175.9 ^e^ s	175.8 ^e^ s
	2	55.7 ^d^ s	55.8 ^d^ s	55.8 s	55.8 s	55.8 s	55.7 ^d^ s	55.8 ^d^ s
	3	26.5 ^g^ q	26.8 q	26.5 ^g^ q	26.5 q	26.5 q	26.5 q	26.3 q
	4	24.1 q	24.2 q	24.1 q	24.1 q	24.1 q	24.1 q	23.7 q
^2^Ala	1	174.8 s	174.6 s	174.6 s	174.5 s	174.6 s		174.6 s
	2	51.0 d	51.0 d	51.0 d	50.8 d	50.9 d	50.9 d	50.9 d
	3	16.8 q	16.8 q	16.8 q	16.8 q	16.9 q	16.8 q	16.9 q
^3^Ala	1	174.8 s	174.8 s	174.8 s	174.5 s	174.6 s	174.7 s	174.6 s
	2	51.4 d	51.3 d	51.3 d	51.0 d	51.1 d	51.2 d	51.1 d
	3	16.1 q	16.1 q	16.1 q	16.2 q	16.2 q	16.1 q	16.2 q
^4^Aib	1	175.0 s	175.0 s	175.0 s	175.0 s	175.0 s	175.0 s	175.0 s
	2	56.0 s	56.0 s	55.7 ^d^ s	55.9 ^d^ s	56.4 s	56.0 ^d^ s	56.4 s
	3	26.2q	25.8 ^j^ q	26.2 ^g^ q	26.5 q	26.0 ^g^ q	26.1 q	26.3 ^g^ q
	4	23.3 ^f^ q	22.6 ^f^ q	23.4 ^h^ q	22.9 ^h^ q	23.1 ^f^ q	22.5 ^f^ q	23.3 ^f^ q
^5^Aib/^5^Iva	1	176.0 ^e^ s	176.0 ^e^ s	175.9 ^e^ s	176.4 s	176.2 s	176.0 ^e^ s	176.2 s
	2	55.8 ^d^ s	55.7 ^d^ s	55.8 ^d^ s	58.6 s	58.5 s	55.8 ^d^ s	58.5 s
	3	26.7 ^g^ q	26.7 ^g^ s	26.5 ^g^ q	25.6 t	25.8 t	26.6 q	25.9 t
	4	22.6 q	22.8 ^f^ q	22.6 ^h^ q	7.4 q	7.4 q	22.7 ^f^ q	7.4 q
	5				22.7 ^h^ q	22.7 q		22.6 q
^6^Gln	1	174.1 s	174.1 s	174.1 s	173.8 s	174.1 s	173.8 s	174.1 s
	2	56.2 ^d^ d	56.4 d	56.4 d	56.0 d	56.0 d	56.0 d	56.0 d
	3	26.2 t	26.5 t	26.2 t	26.2 t	26.2 t	26.2 t	26.2 t
	4	31.5 ^c^ t	31.5 ^c^ t	31.5 ^c^ t	31.6 ^c^ t	31.6 ^c^ t	31.5 ^c^ t	31.7 ^c^ t
	5	173.6 s	173.6 ^k^ s	173.6 ^f^ s	173.8 s	173.6 s	173.7 s	173.6 ^f^ s
^7^Aib	1	176.0 ^e^ s	176.0 ^e^ s	176.0 ^e^ s	175.7 ^e^ s	176.0 ^e^ s	176.0 ^e^ s	176.0 ^e^ s
	2	56.0 s	56.0 s	55.9 ^d^ s	55.9 ^d^ s	56.4 ^d^ s	55.9 ^d^ s	56.2 ^d^ s
	3	26.4 ^g^ q	26.2 ^j^ q	26.5 ^g^ q	26.2 ^g^ q	26.3 ^g^ q	26.2 q	26.2 ^g^ q
	4	23.0 q	23.0 ^f^ q	22.9 ^h^ q	22.6 ^h^ q	22.7 ^e^ q	22.8 ^f^ q	22.9 ^f^ q
^8^Aib	1	176.3 s	176.2 s	176.2 s	175.9 ^e^ s	176.4 s	175.6 s	176.4 s
	2	56.0 s	56.0 s	56.0 ^d^ s	56.1 ^d^ s	56.0 s	56.0 ^d^ s	56.1 ^d^ s
	3	26.8 ^h^ q	26.8 q	26.8 ^g^ q	25.8 ^g^ q	26.8 ^g^ q	26.6 ^g^ q	26.4 ^g^ q
	4	22.7 q	23.2 ^h^ q	23.3 ^h^ q	23.0 ^h^ q	22.9 ^e^ q	22.8 ^f^ q	23.2 ^e^ q
^9^Ala/^9^Aib	1	174.7 s	174.6 s	174.6 s	176.5 s	174.6 s	176.4 s	174.6 s
	2	51.9 d	51.8 d	51.8 d	56.0 ^d^ s	51.7 d	56.1 ^d^ s	51.7 d
	3	16.5 q	16.5 q	16.5 q	26.2 ^g^ q	16.5 q	26.5 ^g^ q	16.5 q
	4				23.2 ^h^ q		22.9 ^f^ q	
^10^Ser	1	170.7 s	170.7 s	170.7 s	170.6 s	170.6 s	170.6 s	170.6 s
	2	58.3 d	58.2 d	58.2 d	58.8 d	58.1 d	58.9 d	58.1 d
	3	61.1 t	61.1 t	61.1 t	61.0 t	61.1 t	61.2 t	61.0 ^h^ t
^11^Leu	1	173.5 s	173.3 s	173.3 s	173.5 s	173.3 s	173.7 s	173.3 s
	2	51.6 d	51.5 d	51.5 d	51.4 d	51.4 d	51.6 d	51.4 d
	3	39.5 t	39.5 t	39.5 t	39.8 t	39.8 t	39.2 t	39.8 t
	4	24.0 d	24.2 d	24.2 d	24.1 d	24.2 d	23.9 d	24.0 d
	5	20.6 q	21.0 q	20.8 q	20.8 q	20.9 q	20.5 q	21.0 q
	6	23.0 q	23.1 q	23.1 q	23.4 q	22.9 q	23.0 q	22.9 q
^12^Aib	1	173.0 s	172.7 s	172.8 s	172.8 s	172.8 s	173.0 s	172.7 s
	2	55.9 s	56.4 s	56.1 ^d^ s	56.1 ^d^ s	56.2 ^d^ s	56.3 ^d^ s	56.1 ^d^ s
	3	25.6 q	26.3 ^g^ q	26.4 ^g^ q	26.4 ^g^ q	26.4 q	25.8 q	25.5 q
	4	23.4 ^f^ q	23.1 q	23.2 ^h^ q	22.6 q	23.0 q	23.1 q	22.9 q
^13^Pro	1	173.4 s	173.8 s	173.8 s	173.7 s	173.8 s	173.3 s	173.8 s
	2	63.0 d	63.0 d	63.0 d	63.0 d	63.0 d	63.0 d	63.0 d
	3	28.8 t	28.8 t	28.8 t	28.7 t	28.7–28.8 t	28.7 t	29.1 t
	4	25.9 t	25.8 t	25.8 t	26.0 t	25.9 t	26.1 t	26.0 t
	5	48.7 t	48.6 t	48.6 t	48.5 t	48.6 t	48.7 t	48.6 t
^14^Leu/^14^Val	1	173.7	172.7	172.6	172.7 s	172.7 s	173.8 s	172.7 s
	2	53.1 d	61.0 d	61.1 d	61.1 d	61.1 d	53.6 d	61.1 ^h^ d
	3	38.7 t	28.8 d	28.8 d	28.8 d	28.7–28.8 d	38.7 t	28.8 d
	4	24.8 d	19.1 q	19.1 ^k^ q	19.1 q	19.2 q	24.8 d	19.2 q
	5	23.0 q	19.1 q	19.2 ^k^ q	19.2 q	19.1 q	23.0 q	19.2 q
	6	21.1 q					20.9 q	
^15^Aib	1	175.4 s	175.7 s	175.6 s	175.6 s	175.7 s	175.3 s	175.7 s
	2	56.3 s	56.4 s	56.2 ^d^ s	55.9 ^d^ s	56.1 ^d^ s	56.2 ^d^ s	56.1 ^d^ s
	3	26.3 ^g^ q	26.4 ^g^ q	26.2 ^g^ q	26.6 ^g^ q	26.5 ^g^ q	26.1 ^g^ q	26.3 ^g^ q
	4	23.4 ^f^ q	23.4 ^f^ q	22.9 ^h^ q	23.3 ^h^ q	23.3 ^f^ q	23.4 ^f^ q	23.2 ^f^ q
^16^Ile	1	172.0 s	172.4 s	172.3 s	172.2 s	172.3 s	171.9 s	172.4 s
	2	58.9 d	59.6 d	59.5 d	59.5 d	59.5 d	58.9 d	59.6 d
	3	35.6 d	35.5 d	35.5 d	35.5 d	35.5 d	35.6 d	35.5 d
	4	25.0 t	25.4 t	25.3 t	25.4 t	25.4 t	25.0 t	25.4 t
	5	11.4 q	11.4 q	11.4 q	11.4 q	11.4 q	11.5 q	11.4 q
	6	15.7 q	15.7 q	15.7 q	15.7 q	15.7 q	15.7 q	15.7 q
^17^Glu/^17^Gln	1	171.5 s	171.8 s	171.4 s	171.6 s	171.4 s	171.1 s	171.8 s
	2	53.8 d	54.3 d	53.7 d	53.8 d	53.7 d	53.1 d	54.3 d
	3	27.1 t	26.8 t	26.0 t	26.3 t	26.3 t	26.5 t	26.8 t
	4	31.7 ^c^ t	31.8 ^c^ t	30.2 t	30.5 t	30.2 t	30.1 t	31.7 ^c^ t
	5	173.7 s	173.7 ^k^ s	173.0 s	172.9 s	173.0 s	172.9 s	173.7 ^f^ s
	OMe			51.4 q	51.4 q	51.4 q	51.4 q	
^18^Gln	1	170.9 s	171.0 s	170.9 s	170.9 s	170.9 s	170.8 s	171.0 s
	2	53.1 d	53.4 d	53.3 d	53.3 d	53.3 d	53.0 d	53.4 d
	3	27.7 t	27.5 t	27.5 t	27.5 t	27.5 t	27.7 t	27.4 t
	4	31.7 ^c^ t	31.7 ^c^ t	31.7 ^c^ t	31.7 ^c^ t	31.7 ^c^ t	31.6 ^c^ t	31.5 ^c^ t
	5	173.7	173.8 ^k^ s	173.5 ^f^ s	173.6 s	173.6	173.6 s	173.8 ^f^ s
^19^PheOH	1	62.7 t	62.9 t	62.8 t	62.8 t	62.8 t	62.7 t	62.9 t
	2	52.6 d	52.6 d	52.6 d	52.6 d	52.6 d	52.5 d	52.6 d
	3	36.7 t	36.7 t	36.7 t	36.7 t	36.7 t	36.6 t	36.7 t
	4	139.2 s	139.3 s	139.3 s	139.3 s	139.3 s	139.2 s	139.3 s
	5	129.4 d	129.4 d	129.4 d	129.4 d	129.4 d	129.4 d	129.4 d
	6	128.2 d	128.1 d	128.1 d	128.1 d	128.1 d	128.1 d	128.1 d
	7	126.0 d	126.0 d	125.9 d	126.0 d	126.0 d	126.0 d	126.0 d

^a^ Instrument ^13^C frequency 100 MHz; ^b^ Instrument ^13^C frequency 125 MHz; ^c,d,e,f,g,h,k^ These signals may interchange in columns.

### 2.3. Determination of the Absolute Stereochemistry

Marfey’s analysis [15] of TA1938 (**1**) using l-FDAA as derivatizing reagent established the l-configuration of Ile, Leu × 2, Glu × 3 (one from glutamic acid, two from glutamine), Pro and Ser. Advanced Marfey analysis [16] of TA1938 using l-FDAA and d-FDAA as derivatizing agents and analysis by LC/MS established the l-configuration of Ala × 2. Iva configuration was not established, and Aib is not chiral. Configuration of phenylalaninol was established as l in TA1909 (**2**) by Marfey’s analysis preceded by Jones oxidation [17] and comparison to the Phe standard and assumed as l in other compounds, due to the similarity of their structures. Marfey’s analysis of TA1910 (**6**) using l-FDAA as derivatizing reagent established the l-configuration of Ile, Leu, Glu × 3 (one from glutamic acid, two from glutamine), Pro, Ser and Val. Marfey’s analysis of TA1895 (**5**) using l-FDAA as derivatizing reagent established the l-configuration of Val. The configurations of other amino acids were established by comparison of Marfey’s chromatograms to those of TA1938 (**1**). Retention times were found similar, meaning that absolute configurations of amino acids are as in TA1938 (**1**). Marfey’s analysis of TA1909 (**2**), TA1896 (**4**), TA1924 (**5**), TA1924A (**7**) and TA1909A (**8**) using l-FDAA was performed by comparison to Marfey’s chromatograms of TA1938 (**1**) and other peptaibols.

### 2.4. Antibacterial Bioassay

**Figure 3:**
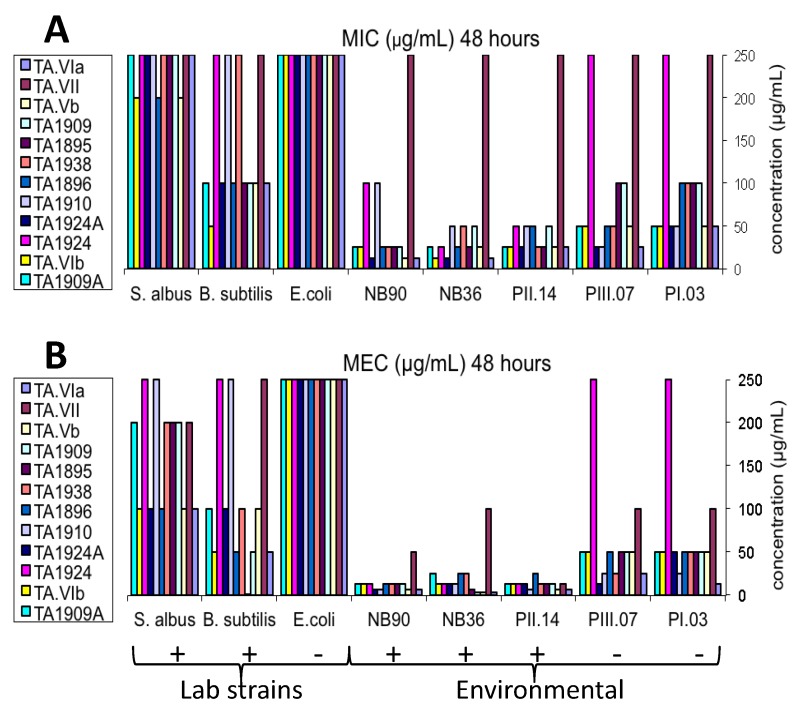
Antibacterial activity of trichorzianines after 48 h (**A**) Minimal inhibitory concentration (MIC) (µg/mL); (**B**) minimal effective concentration (MEC) (µg/mL). + represents Gram-positive bacteria; −, Gram-negative bacteria. Value of 250 µg/mL means no activity (MIC > 200 µg/mL).

The antibacterial activity of the isolated trichorzianines (**1**–**12**) was tested against five environmental bacteria and three laboratory bacterial strains (detailed in the Experimental Section). MIC (minimal inhibitory concentration) was designated as the lowest concentration in which bacterial growth was inhibited to 0%–10% and MEC (minimal effective concentration) as the lowest concentration in which bacterial growth was inhibited to 70%. The results that were obtained after 48 h of incubation are summarized in Figure 3. 

Examination of the trichorzianines activity (Figure 3) shows a general pattern. The tested trichorzianines exhibited stronger activity against environmental bacteria than against the laboratory strains. When compared within each group (environmental/laboratory), Gram-positive bacteria were more sensitive than Gram-negative bacteria (*E*. *coli* was resistant to all the tested compounds at all concentrations). Upon comparison of pairs of compounds differing in a single amino acid, no correlation was found between change in the amino acid sequence and activity. 

## 3. Experimental Section

### 3.1. General Experimental Procedures

MS data were acquired using MALDI-Synapt Waters mass spectrometer using positive ES ionization. UV spectra were recorded on an Agilent 8453 spectrophotometer. Optical rotation values were obtained on a Jasco P-1010 polarimeter. IR spectra were recorded on a Bruker Vector 22 spectrophotometer. ^1^H- and ^13^C-NMR and 2D NMR data were recorded on spectrometers: Bruker ARX 500 (500.13 MHz for ^1^H, 125.8 MHz for ^13^C), Bruker AVANCE 400 (400.13 MHz for ^1^H, 100.62 MHz for ^13^C) and Bruker AVANCE 400 (400.17 MHz for ^1^H, 100.63 MHz for ^13^C), at room temperature, in 5 mm NMR tubes, with tetramethylsilane (TMS, δ 0.0 ppm) as the internal standard. COSY-45, gTOCSY, gROESY, gHSQC and gHMBC spectra were recorded using standard Bruker pulse sequences. HPLC chromatography rations were performed on Jasco (model PU-2080 plus intelligent pump, model LG-2080-04 plus quaternary gradient unit), Merck-Hitachi (model L-6200A intelligent pump, model L-4200 UV-Vis detector) and Agilent 1100 series HPLC system.

### 3.2. Biological Material

The sponge, *Axinella* sp., was collected by SCUBA diving at a depth of 19 m in the Mediterranean Sea, Akhziv, Israel. The sponge sample was sealed underwater in a bag with seawater and brought, cooled, to the laboratory for isolation and culturing of fungi. A pure fungal strain was obtained by inoculating slices of the sponge (initially rinsed with sterile Ca^2+^- and Mg^2+^-free artificial seawater) in potato dextrose agar (PDA, Difco) tubes containing chloramphenicol (0.25 g/L) and subsequent re-inoculations on PDA plates. Fungal genomic DNA was extracted from 15–20 mg of ground lyophilized mycelia grown in Potato Dextrose Broth (PDB, Difco), as described in [18]. The fungus was identified as *Trichoderma atroviride* based on its PCR-amplified ITS region, using primers ITS1 and ITS4 [19] and cycling parameters as in [20], followed by sequencing and comparison to the GenBank database (Trichokey [21], BLAST [22]). The strain was maintained on PDA. To determine the abundance of *T*. *atroviride* in the sponges, 25 new *Axinella* spp. specimens were collected. Pure fungal strains were isolated by dispersing 100 µL of the liquid obtained from a sponge sample ground by pestle and mortar on PDA plates containing chloramphenicol (0.25 g/L) and subsequent re-inoculations on PDA plates. Other details of fungal isolation were as described above. DNA was extracted by a protocol based on [23] using SDS–lysozyme-proteinase K lysis.

### 3.3. Culture Procedure

For the initial screening, a culture of 1 L of the fungus was grown. The culture was incubated for three weeks, at room temperature, in the horizontal position in 850-mL tissue culture flasks containing 200 mL of PDB. For the isolation of the active compounds, 15 L of the fungus were cultured in the same manner.

### 3.4. Isolation Procedure

The medium was filtered through glass wool to separate it from the mycelium and adsorbed on Amberlite XAD7HP, followed by washing of the organic compounds from the resin with acetone (method based on [24]). Briefly, after adding 20 g of Amberlite XAD-7-HP resin for each liter of medium, the medium was stirred for two h on a magnetic stirrer, followed by decanting the liquid. Then, the resin was washed with two small portions of acetone in order to remove water, extracted twice with acetone and washed with distilled water. The acetone was filtered and evaporated. The 15-L medium yielded 8.3 g of the crude extract. During initial separation, the fractionation was guided by ^1^H-NMR and anti-bacterial assays. At the later stages of the separation, while according to NMR and MS spectra, it became clear that there was a mixture of peptaibols, it was determined by MS and MS/MS whether the fraction was pure or required further purification. The crude extract was separated on a Sephadex LH-20 column using MeOH–CHCl_3_ (1:1). Fractions 1–6 from this separation (5.8 g) were further separated on a Sephadex LH-20 column using MeOH–CHCl_3_ (2:1). Fractions 1–6 from this separation (4.2 g) were separated on an open column C_18_-reversed phase column and eluted with solvent of decreasing polarity from water to methanol (10% steps of methanol each fraction). Fractions 8–11 and column wash in methanol (total weight 3.0 g) were separated on a Sephadex LH-20 using MeOH–CHCl_3_ (1:1). Fractions 1–13 (2.7 g) were separated again on a Sephadex LH20, using MeOH–H_2_O (7:3). Fractions 4–8 from this separation (2.36 g) were separated on an HPLC column (Hibar, Lichrospher 60 RP-Select B, 5 µm, 250 × 20 mm, flow rate 5 mL/min, UV detection, eluent 55–50:45–50 acetonitrile/water), to give 14 fractions, four of which (fractions 6 to 9) were found to be active. Fraction 7 (236 mg) was separated by HPLC (Hibar, Lichrospher 60 RP-Select B, 5 µm, 250 × 20 mm, flow rate 5 mL/min, UV detection, eluent 53:47 acetonitrile/aq. TFA (0.1%)) followed by HPLC separation (Cosmosil, 5C-8 MS Waters, 250 × 20 mm, UV detection, eluent with 81.5–83:18.5–17 methanol/aq. TFA (0.1%)) were used to afford TA1909 (*t*_R_ 92 min, 5.2 mg) and TA1895 (*t*_R_ 99 min, 3.7 mg). Fraction 6 (391.5 mg) was separated by HPLC (Hibar, Lichrospher 60 RP-Select B, 5 µm, 250 × 20 mm, flow rate 5 mL/min, UV detection, eluent 53:47 acetonitrile/aq. TFA (0.1%)) to two fractions: one yielded TA1896 (4.8 mg) and the second yielded, again, TA1909 (6.2 mg), as well as TA1895 (2.4 mg), by following HPLC separations. Fraction 8 (291.3 mg) was separated by repeated HPLC separations, resulting in TA1909a (2.2 mg), TA.VIb (5.2 mg), TA.Vb (9.7 mg), TA.VIa (1.8 mg), TA1924a (2.2 mg), TA.VII (1.9 mg) and TA1910 (4.0 mg). HPLC separations of Fraction 9 yielded TA1938 (7.8 mg), TA1924 (2.8 mg), TA.VIa (8.3 mg) and TA.VII (6.7 mg). 

Trichorzianine 1938 (**1**): amorphous white solid; 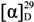 −25 (*c* 0.68, MeOH); UV (MeOH) λ_max_ (log ε) 203 (4.61); IR (KBr) ν_max_ 3320, 2960, 2360, 1661, 1541, 1204 cm^−1^; ^1^H and ^13^C NMR (see Table 2); HR TOF-MS-ES^+^ *m*/*z* 1961.1099 [M + Na]^+^ (calcd for C_91_H_151_N_21_NaO_25_, 1961.1088). Retention time of amino acid (AA) Marfey’s derivatives: l-Ile 44.5 min (d-Ile 47.1 min), l-Leu 45.3 min (d-Leu 47.7 min), l-Pro 35.8 min (d-Pro 36.4 min), l-Glu 32.7 min (d-Glu 33.6 min) and l-Ser 29.7 min (d-Ser 33.2 min). Advanced Marfey: l-Ala (l-Ala-l-FDAA, 3.40 min; l-Ala-d-FDAA, 3.99 min). Iva, not determined; Pheol, (l as in TA1909).

Trichorzianine 1909 (**2**): amorphous white solid; 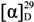 −23 (*c* 0.4, MeOH); UV (MeOH) λ_max_ (log ε) 203 (4.46); IR (ATR probe) ν_max_ 3309, 1647, 1541, 1201 cm^−1^; ^1^H and ^13^C NMR (see Table 3 and Table 4 and Supplementary Table S3); HR TOF-MS-ES^+^ *m*/*z* 1932.0946 [M + Na]^+^ (calcd for C_89_H_148_N_22_NaO_24_, 1932.0935). Retention time of amino acid (AA) Marfey’s derivatives: l-Ile 44.2 min (d-Ile 47.8 min), l-Leu 45.0 min (d-Leu 47.7 min), l-Pro 35.5 min (d-Pro 36.4 min), l-Glu 32.7 min (d-Glu 33.6 min), l-Pheol: as l-Phe 45.6 min (D-Phe 47.7 min), l-Ser 29.4 min (d-Ser 33.2 min) and Ala, (l as in TA1938).

Trichorzianine 1895 (**3**): white powder; 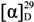 −32 (*c* 0.26, MeOH); UV (MeOH) λ_max_ (log ε) 203 (4.48); IR (ATR probe) ν_max_ 3308, 2936, 1638, 1537, 1202, 1047 cm^−1^; ^1^H and ^13^C NMR (see Table 3 and Table 4 and Supplementary Table S4); HR TOF-MS-ES^+^ *m*/*z* 1918.0864 [M + Na]^+^ (calcd for C_88_H_146_N_22_NaO_24_, 1918.0778). Retention time of amino acid (AA) Marfey’s derivatives: l-Ile 44.3 min (d-Ile 47.8 min), l-Leu 45.0 min (d-Leu 47.7 min), l-Pro 35.6 min (d-Pro 36.4 min), l-Glu 32.7 min (d-Glu 33.6 min), l-Ser 29.5 min (d-Ser 33.2 min) and l-Val 40.9 min (d-Val 43.7 min). Pheol, (l as in TA1909); Ala, (l as in TA1938).

Trichorzianine 1896 (**4**): amorphous, white solid; 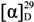 −18 (*c* 0.48, MeOH); UV (MeOH) λ_max_ (log ε) 203 (4.56); IR (ATR probe) ν_max_ 3312, 1653, 1540, 1201 cm^−1^; ^1^H and ^13^C NMR (see Table 3 and Table 4 and Supplementary Table S5); HR TOF-MS-ES^+^ *m*/*z* 1933.0819 [M + Na]^+^ (calcd for C_89_H_147_N_21_NaO_25_, 1933.0775). Retention time of amino acid (AA) Marfey’s derivatives: l-Ile 44.3 min (d-Ile 47.8 min), l-Leu 45.1 min (d-Leu 47.7 min), l-Pro 35.7 min (d-Pro 36.4 min), l-Glu 32.7 min (d-Glu 33.6 min), l-Ser 29.6 min (d-Ser 33.2 min), l-Val 41.0 min (d-Val 43.7 min), Pheol, (l as in TA1909) and Ala, (l as in TA1938).

Trichorzianine 1924 (**5**): amorphous, white solid; 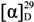 −17 (*c* 0.26, MeOH); UV (MeOH) λ_max_ (log ε) 203 (4.48); IR (KBr) ν_max_ 3335, 2966, 2363, 1663, 1541, 1207 cm^−1^; ^1^H and ^13^C NMR (see Table 3 and Table 4 and Supplementary Table S6); HR TOF-MS-ES^+^ *m*/*z* 1961.1240 [M + Na]^+^ (calcd for C_91_H_151_N_21_NaO_25_, 1961.1088). Retention time of amino acid (AA) Marfey’s derivatives: l-Ile 44.4 min (d-Ile 47.8 min), l-Leu 45.1 min (d-Leu 47.7 min), L-Val 41.0 min (D-Val 43.7 min), l-Pro 35.7 min (d-Pro 36.4 min), l-Glu 32.7 min (d-Glu 33.6 min) and l-Ser 29.3 min (d-Ser 33.2 min). Iva, not determined; Pheol, (l as in TA1909); Ala, (l as in TA1938). 

Trichorzianine 1910 (**6**): amorphous, white solid; 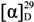 −36 (*c* 0.39, MeOH); UV (MeOH) λ_max_ (log ε) 203 (4.60); IR (ATR probe) ν_max_ 3310, 1654, 1541, 1217 cm^−1^; ^1^H and ^13^C NMR (see Table 3 and Table 4 and Supplementary Table S7); HR TOF-MS-ES^+^ *m*/*z* 1947.0995 [M + Na]^+^ (calcd for C_90_H_149_N_21_NaO_25_, 1947.0931). Retention time of amino acid (AA) Marfey’s derivatives: l-Ile 44.5 min (d-Ile 47.8 min), l-Leu 45.3 min (d-Leu 48.1 min), l-Pro 35.8 min (d-Pro 37.0 min), l-Glu 33.4 min (d-Glu 34.1 min), l-Ser 29.8 min (d-Ser 33.6 min) and l-Val 41.2 min (d-Val 44.1 min). Iva, not determined; Pheol, (l as in TA1909); Ala, (l as in TA1938).

Trichorzianine 1924A (**7**): amorphous, white solid; 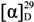 −30 (*c* 0.21, MeOH); UV (MeOH) λ_max_ (log ε) 203 (4.67); IR (ATR probe) ν_max_ 3310, 1655, 1541, 1204 cm^−1^; ^1^H and ^13^C NMR (see Table 3 and Table 4 and Supplementary Table S8); HR TOF-MS-ES^+^ *m*/*z* 1961.1086 [M + Na]^+^ (calcd for C_90_H_149_N_21_NaO_25_, 1961.1088). Retention time of amino acid (AA) Marfey’s derivatives: l-Ile 44.4 min (d-Ile 47.8 min), l-Leu 45.2 min (d-Leu 48.1 min), l-Pro 35.7 min (d-Pro 37.0 min), l-Glu 32.7 min (d-Glu 34.1 min) and l-Ser 29.7 min (d-Ser 33.6 min), Pheol, (l as in TA1909); Ala, (l as in TA1938).

Trichorzianine 1909A (**8**): amorphous, white solid; 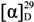 −49 (*c* 0.20, MeOH); UV (MeOH) λ_max_ (log ε) 203 (4.60); IR (ATR probe) ν_max_ 3312, 2292, 1661, 1541, 1204 cm^−1^; ^1^H and ^13^C NMR (see Table 3 and Table 4 and Supplementary Table S9); HR TOF-MS-ES^+^ *m*/*z* 1932.1022 [M + Na]^+^ (calcd for C_89_H_148_N_22_NaO_24_, 1,932.0935). Retention time of amino acid (AA) Marfey’s derivatives: l-Ile 44.5 min (d-Ile 47.8 min), l-Leu 45.2 min (d-Leu 48.1 min), l-Val 41.1 min (d-Val 44.1 min), l-Pro 35.9 min (d-Pro 37.0 min), l-Glu 32.7 min (d-Glu 34.1 min) and l-Ser 29.7 min (d-Ser 33.6 min). Iva, not determined; Pheol, (l as in TA1909); Ala, (l as in TA1938).

### 3.5. Antibacterial Bioassay

Antibacterial activity of the fractions was tested against 3 environmental bacteria: 2 Bacilli from the same sponge from which the fungus was isolated (NB70, NB36) and *Sporosarcina* sp. from the sediment (NB90). Bacteria were cultured in 5 mL liquid media (Marine broth or Lennox broth) on a shaker. According to OD measurement at 620 nm, bacterial cultures were diluted with growth medium to a concentration giving OD absorbance of 0.4 at 620 nm, followed by an additional 1000-fold dilution. Tested fractions were diluted in DMSO to a concentration of 10 mg/mL, followed by further dilutions to 1, 0.5 and 0.25 mg/mL, 10% DMSO in purified water. The test was performed in 96 well plates, in three replicates for each concentration, each replicate on a different plate. The arrangement of the compounds on the plates was random. Into each well, 60 µL of growth media, 40 µL of the compound, and 100 µL of the diluted bacteria were added, to give a final concentrations of 0.2, 0.1 and 0.05 mg/mL, 2% DMSO. Plates were incubated on a shaker at 25 °C. OD was read at the beginning of the experiment (subtracted from later measurements) and after 24 and 48 h. The percentage of growth was calculated relatively to the positive control: a well containing the same bacteria in 2% DMSO without the compounds.

Antibacterial activity of the pure trichorzianines was tested on five environmental bacteria: from the sediment, *Sporosarcina* sp. (NB90), from the same sponge, *Bacillus* sp. (NB36), from *Axinella polypoides* *Microbacterium* sp. (PII.14), Rhodobacteraceae (PI.03), *Shewanella* sp. (PIII.07) and 3 laboratory strains (*S*. *albus*, *B*. *subtilis and E*. *coli*). Bacterial stocks were prepared as described above. Compounds were prepared in DMSO at a concentration of 20 mg/mL and diluted with purified water to 2 mg/mL, 10% DMSO. The test was performed in 96 well plates in liquid medium for bacterial growth (Lennox Broth or Marine Broth) with DMSO at a final concentration of 1% and a series of dilutions of the tested compound, the highest of which was 0.2 mg/mL (for some of the compounds two of the three replicates were prepared at lower concentrations). Into the well containing the highest concentration, 160 µL of medium and 40 µL of the sample (2 mg/mL, 10% DMSO) were added, to give a final concentration of 0.4 mg/mL, 2% DMSO. Into the other wells, 100 µL of the growth medium containing 2% DMSO were inserted, followed by the addition of 100 µL from the previous well. In this manner, a series of 2-fold dilutions was prepared in 2% DMSO, the highest concentration being 0.4 mg/mL. Subsequently, 100 µL of the bacteria were added to each well, diluting their contents to 0.2 mg/mL at the highest concentration, 1% DMSO. OD at 620 nm was measured at the beginning of the experiment (subtracted from later measurements) and after 24 and 48 h of growth. The percentage of growth was calculated relative to the positive control: a well containing the same bacteria in 1% DMSO without the compounds. 

## 4. Conclusions

The *Trichoderma atroviride* strain isolated from Axinellid sponge yielded 12 trichorzianines, eight of which are new and four known. The isolated peptaibols belong to the trichorzianine family. This family was previously isolated from *T*. *harzianum*, as well as *T*. *atroviride*. The profile of the peptaibols described here differs from previous reports on these compounds in *T*. *atroviride*: some of the new compounds include glutamic acid as the seventeenth residue, which contains only glutamine in all known trichorzianines. In contrast, not all of the known compounds were isolated here. For example, none of the isolated compounds contained glutamic acid as the eighteenth residue or tryptophanol as the nineteenth residue. In fact, some fractions contained Trpol, but further purification was impossible, due to the small available quantity of the material.

All isolated trichorzianines exhibited moderate antibacterial activity (MIC 12.5–200 µg/mL) against tested environmental bacteria, except for TA.VII, which showed no activity and TA1924 which did not inhibit Gram-negative bacteria. The absence of activity in tests as seen for TA.VII is not unusual and could indicate either activity on a narrow range of microorganisms or on antibacterial activity that is not expressed in the lab or in liquid medium. Laboratory bacterial strains were much more resistant to the peptaibols: *E*. *coli* exhibited no inhibition, and *S*. *albus* and *B*. *subtilis* were inhibited by some of the peptaibols (MIC 50–200 µg/mL).

## Supplementary Files

Supplementary File 1 Supplementary Materials (PDF, 3895 KB)
