# Supplementary material for: Eight New Peptaibols from Sponge-Associated Trichoderma atroviride
Source: Mar Drugs. 2013 Dec 11;11(12):4937–60. doi: 10.3390/md11124937 (PMC3877895; doi:10.3390/md11124937)
Supplement: Supplementary File 1 — Supplementary Materials (PDF, 3895 KB) [file marinedrugs-11-04937-s001.pdf]

Supplementary Materials

NMR Spectra of the New Compounds

Figure S1. ^{1}H spectrum of TA1938 (**1**).

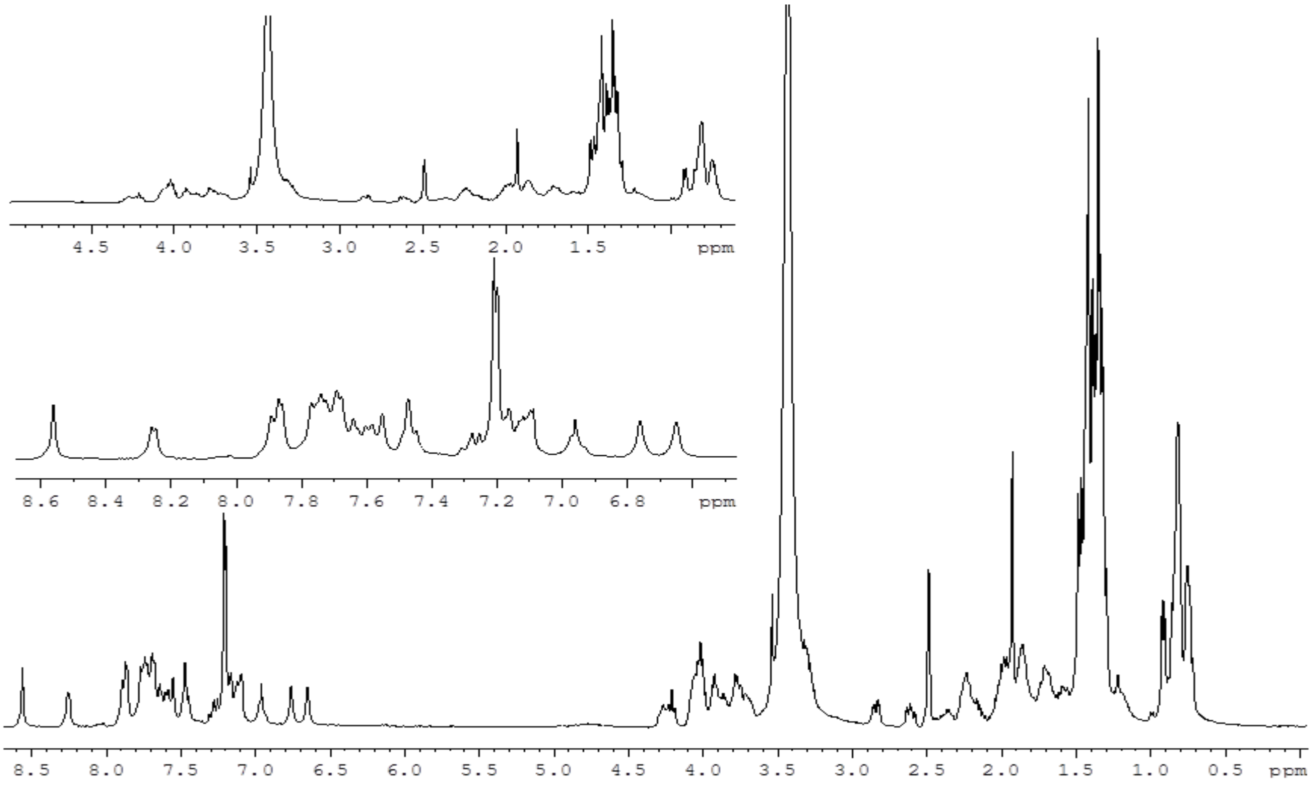

Figure S2. ^{13}C spectrum of TA1938 (**1**).

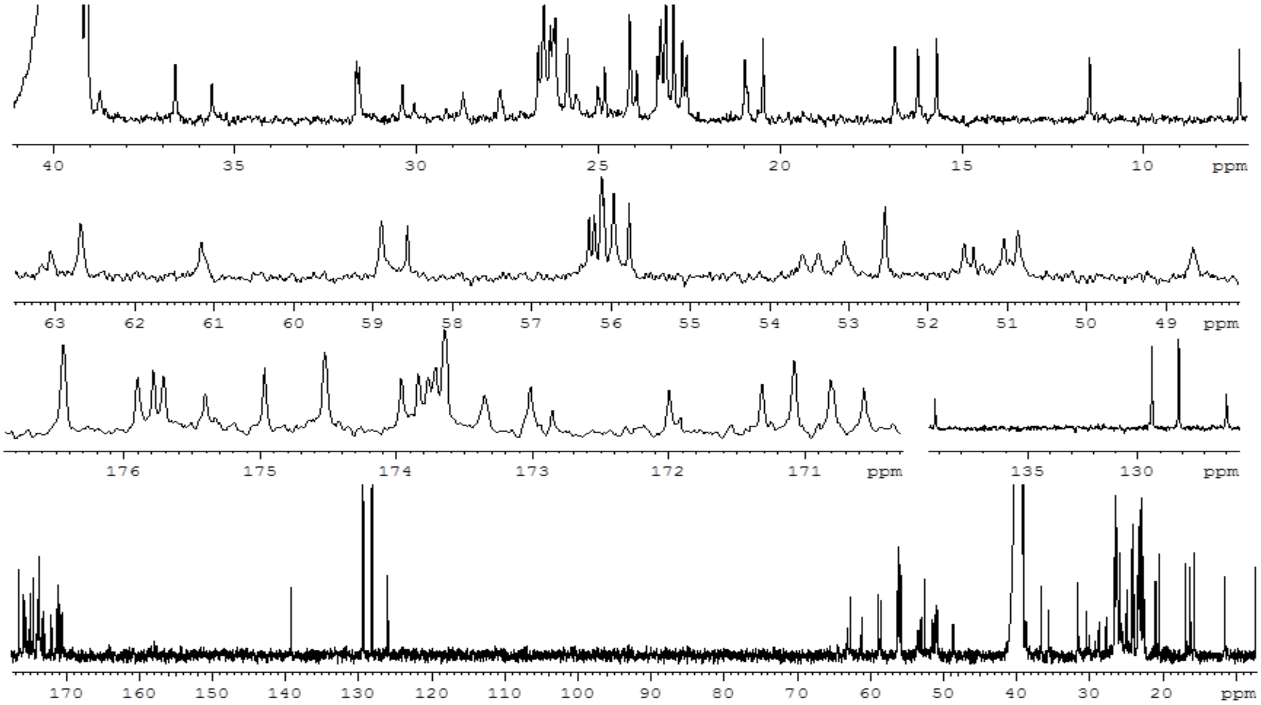

Figure S3. HSQC spectrum of TA1938 (**1**).

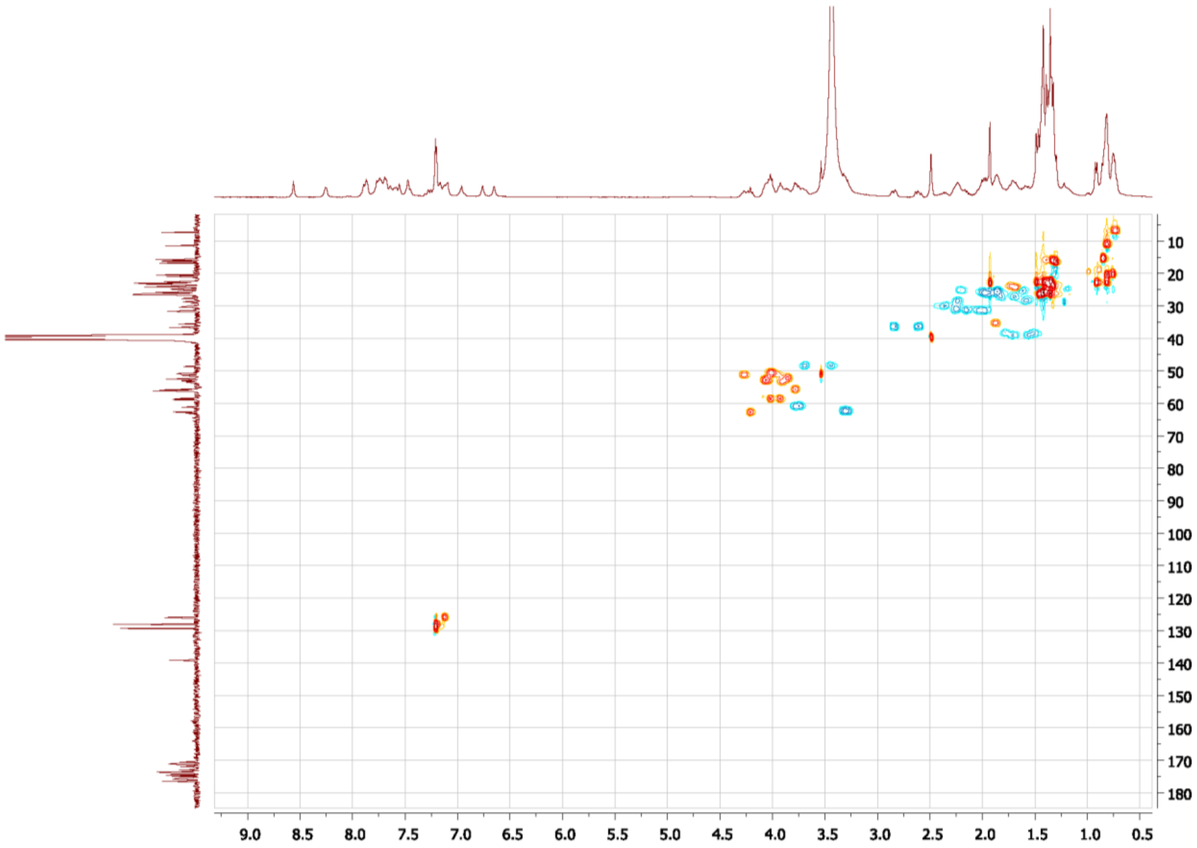

Figure S4. HMBC spectrum of TA1938 (**1**).

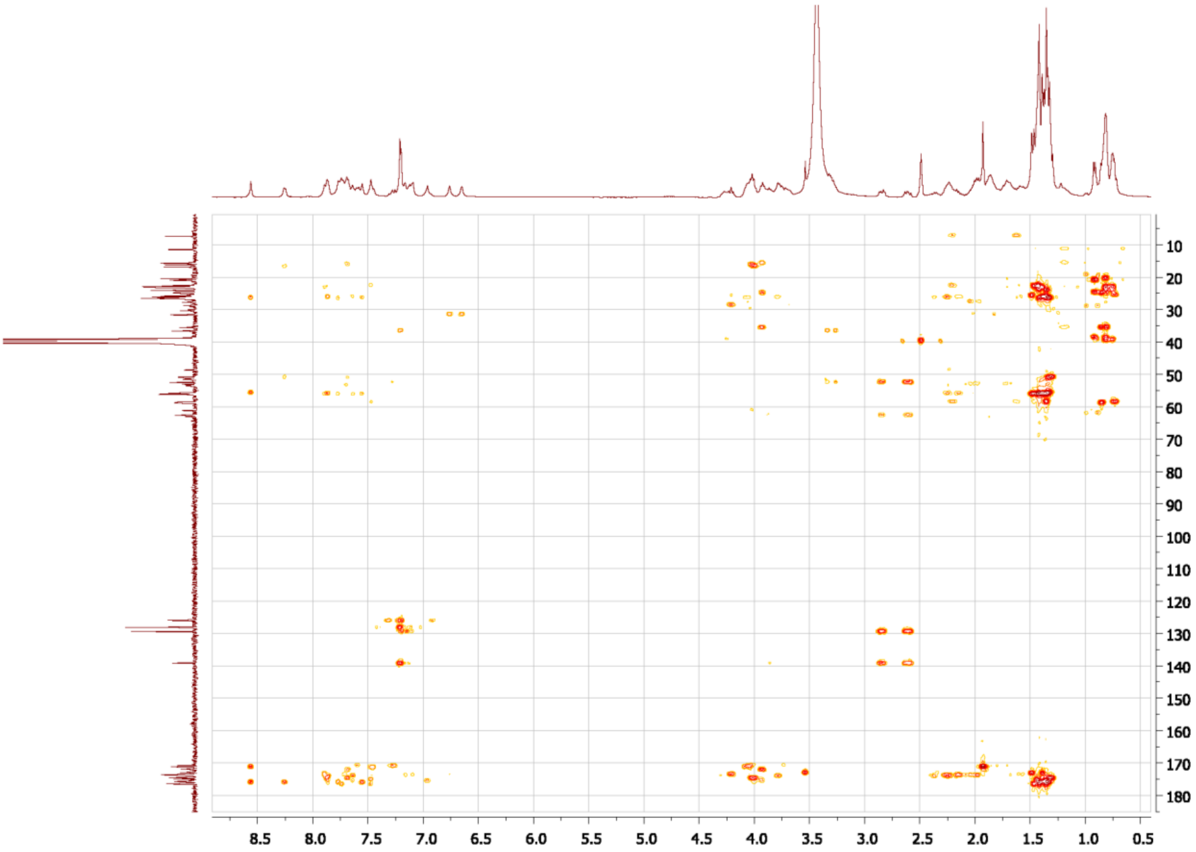

Figure S5. COSY spectrum of TA1938 (**1**).

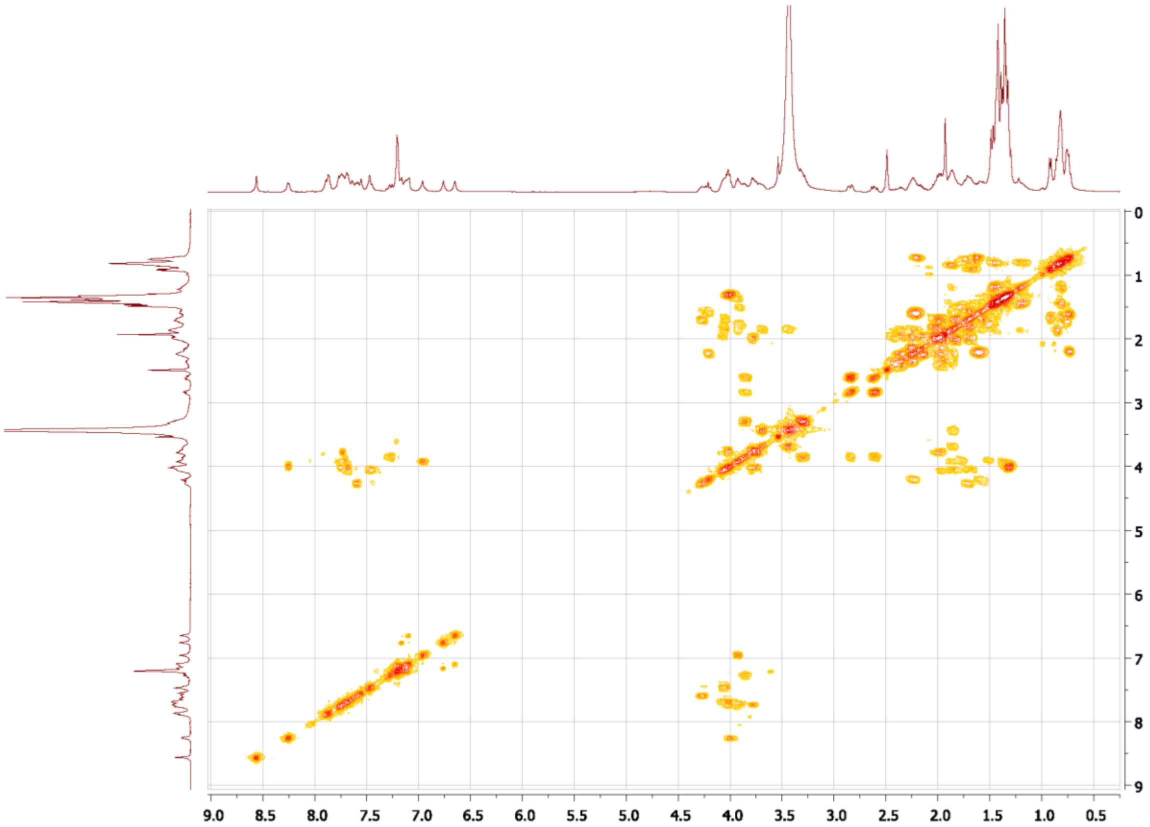

Figure S6. ROESY spectrum of TA1938 (**1**).

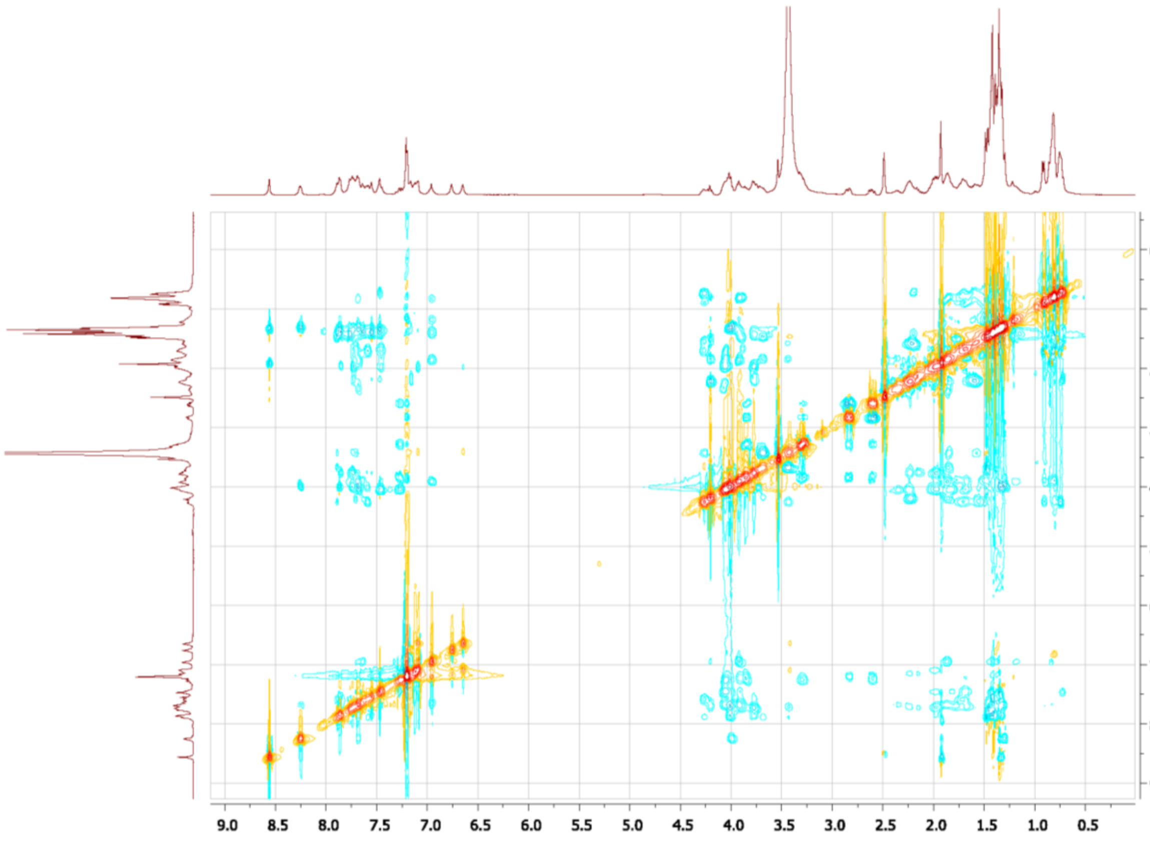

Figure S7. Secondary MS fragmentation of the TA1909 (**2**) ions at *m/z* 1094.6 (**A**) and *m/z* 838.8 (**B**).

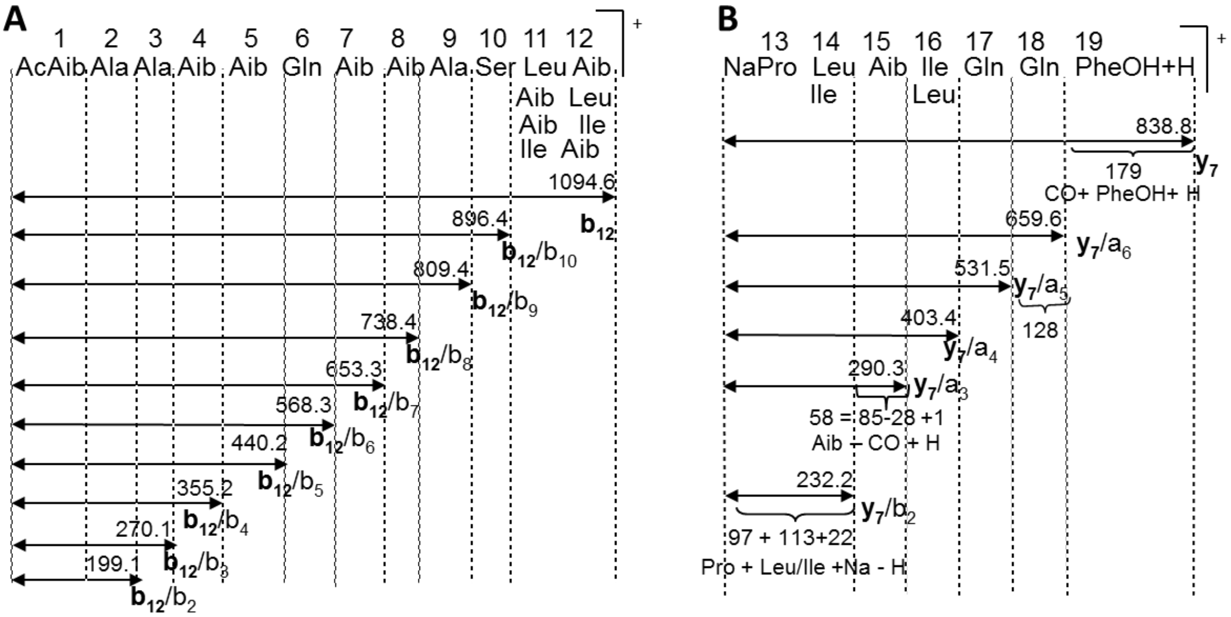

Figure S8. Secondary MS fragmentation of the TA1895 (**3**) ions at *m/z* 1094.6 (**A**) and *m/z* 802.5 (**B**).

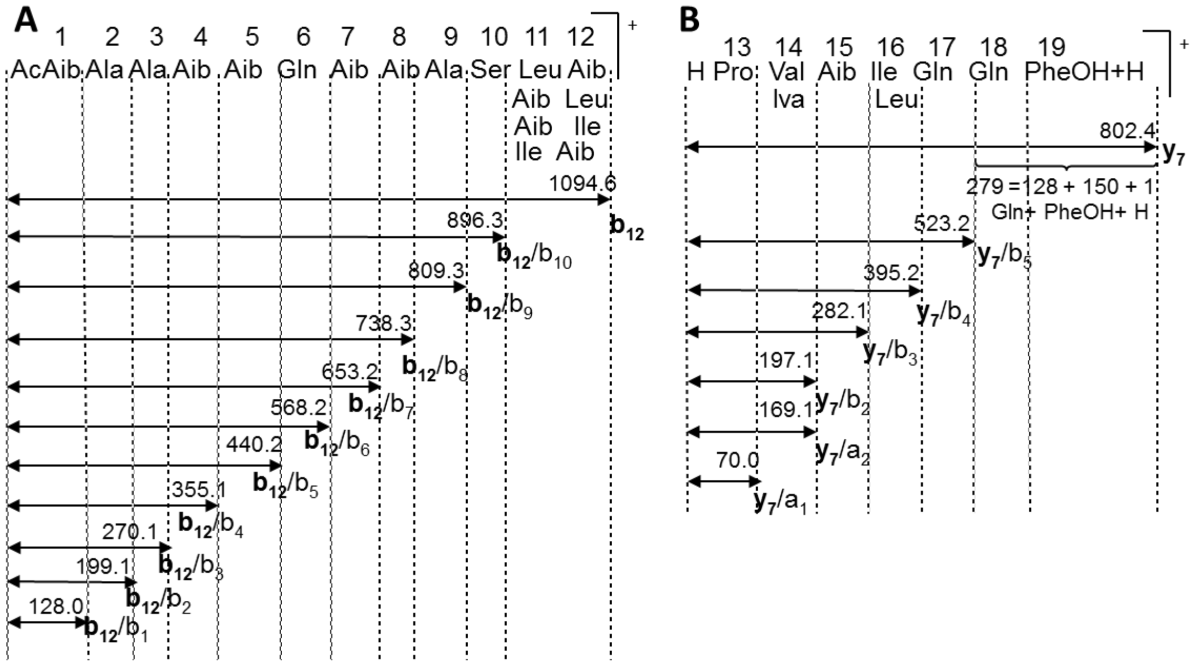

Figure S9. Secondary MS fragmentation of the TA1896 (**4**) ions at *m/z* 1094.6 (**A**) and *m/z* 817.4 (**B**).

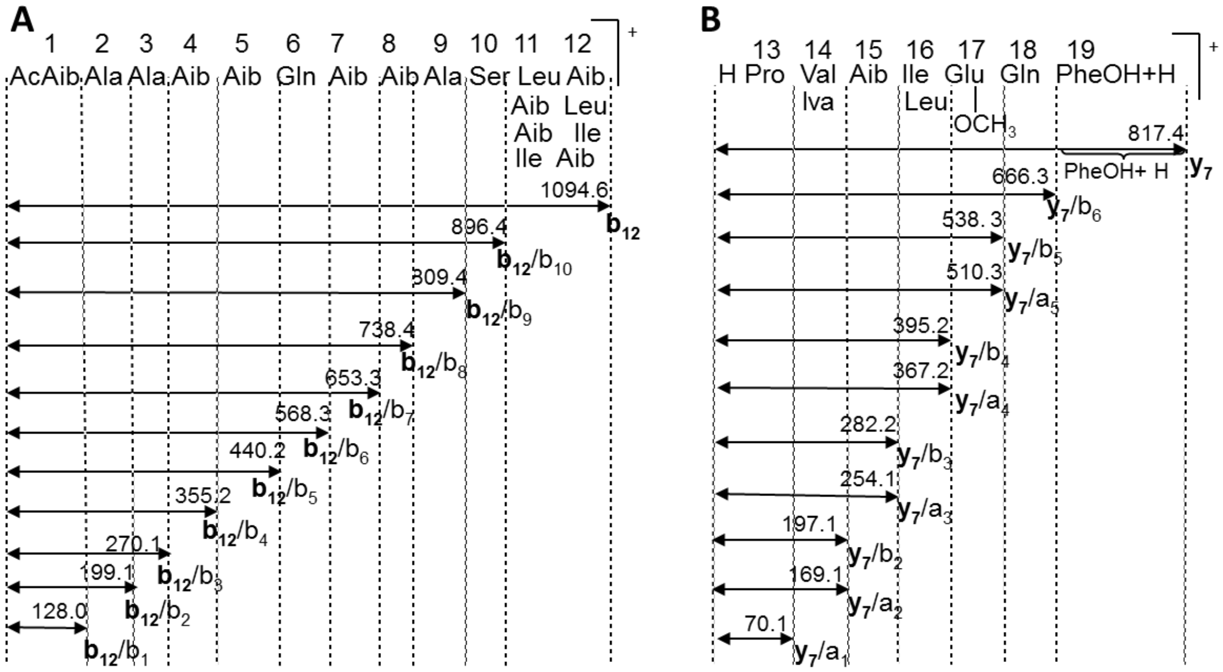

Figure S10. Secondary MS fragmentation of the TA1924 (**5**) ions at *m/z* 1122.7 (**A**) and *m/z* 817.7 (**B**).

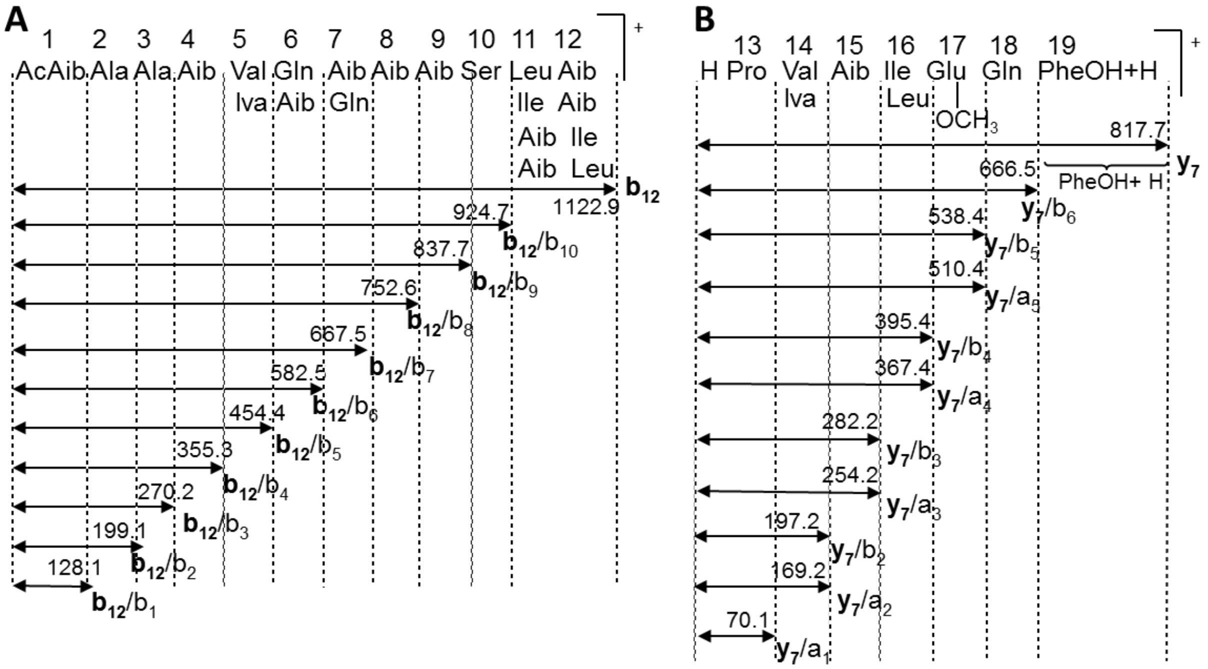

Figure S11. Secondary MS fragmentation of the TA1910 (**6**) ions at *m/z* 1108.5 (**A**) and *m/z* 839.3 (**B**).

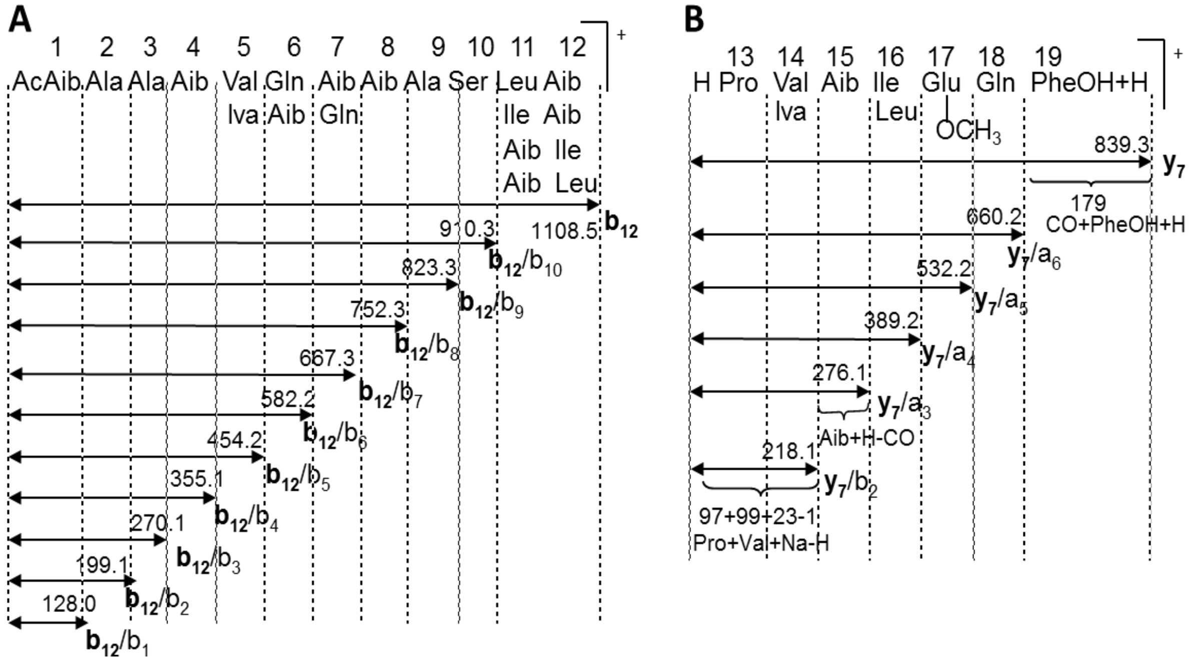

Figure S12. Secondary MS fragmentation of the TA1924a (**7**) ions at *m/z* 1108.7 (**A**) and *m/z* 853.5 (**B**).

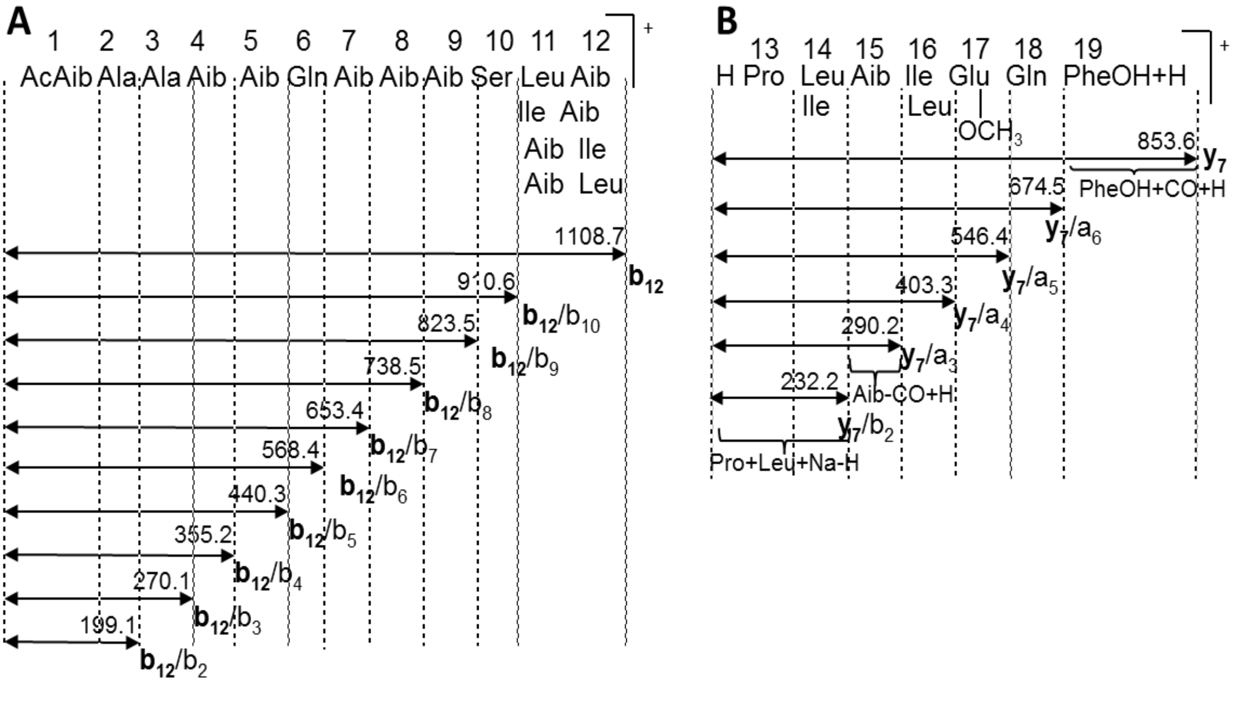

Figure S13. Secondary MS fragmentation of the TA1909a (**8**) ions at *m/z* 1108.5 (**A**) and *m/z* 824.4 (**B**).

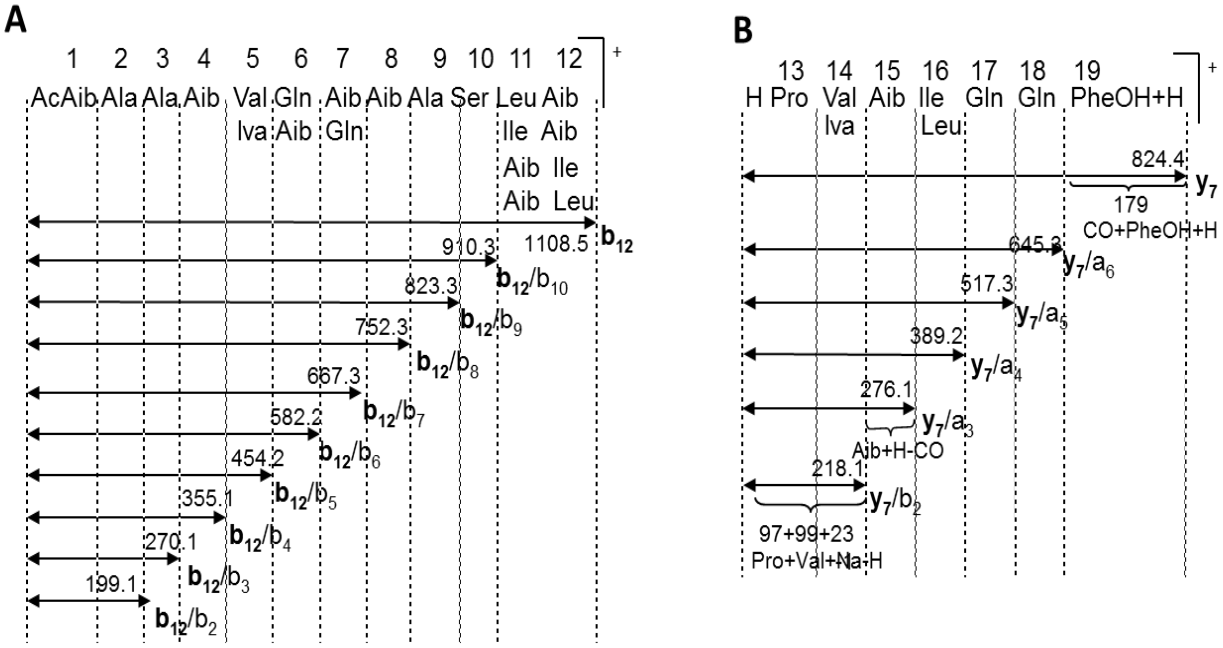

Figure S14. Secondary MS fragmentation of the TA.VIa (**10**) ions at *m/z* 1122.9 (**A**) and *m/z* 816.5 (**B**).

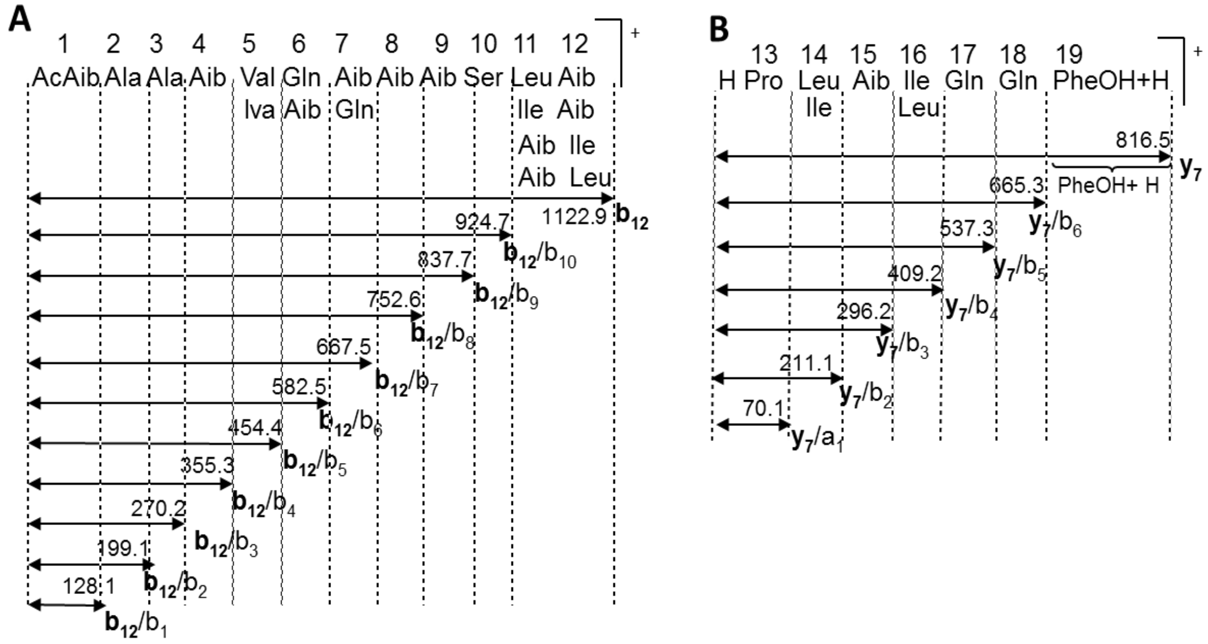

Figure S15. Secondary MS fragmentation of the TA.VII (**11**) ions at *m/z* 1122.8 (**A**) and *m/z* 802.4 (**B**).

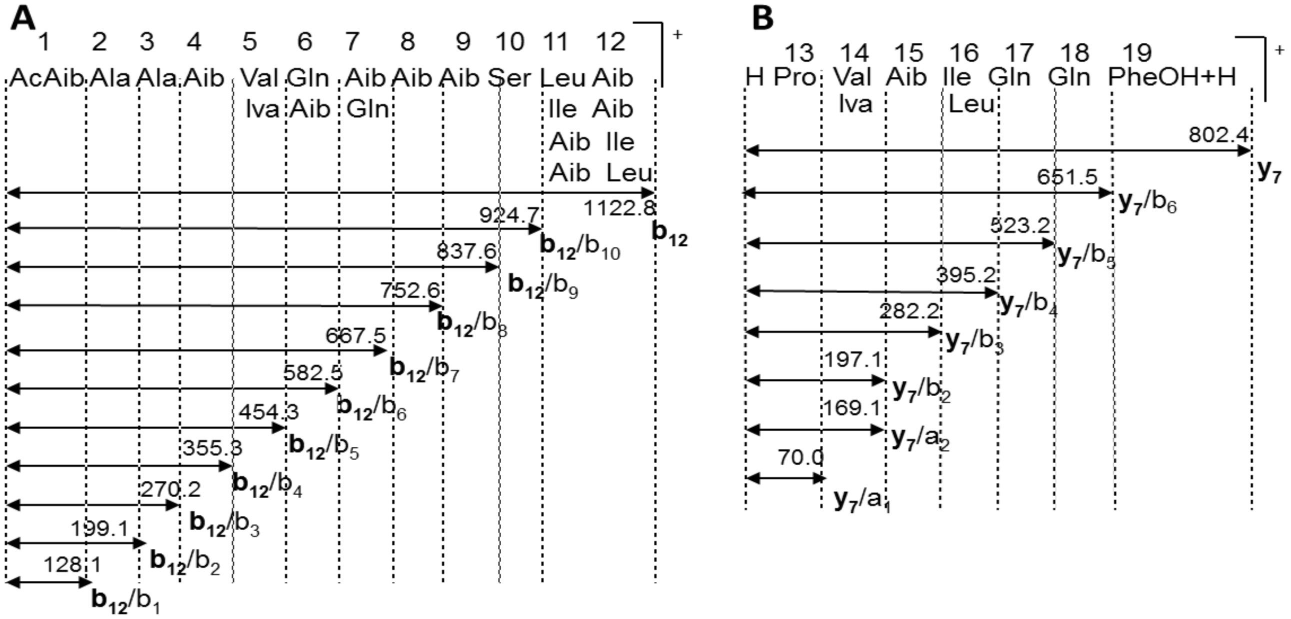

Secondary fragmentation of TA.VIb (**9**): *m/z* 1108.6–see secondary fragmentation of TA1924a (Figure S12A). In addition, peaks are present indicating an impurity of *m/z* 1108.6 with other amino acid sequence–see secondary fragmentation of TA1910 (Figure S11A), *m/z* 824.4–see secondary fragmentation of TA1909a (Figure S13B); Secondary fragmentation of TA.Vb (**12**): *m/z* 1108.8–see secondary fragmentation of TA1924a (Figure S12A), *m/z* 838.6–see secondary fragmentation of TA1909 (Figure S7B).

Figure S16. ^{1}H spectrum of TA1909 (**2**).

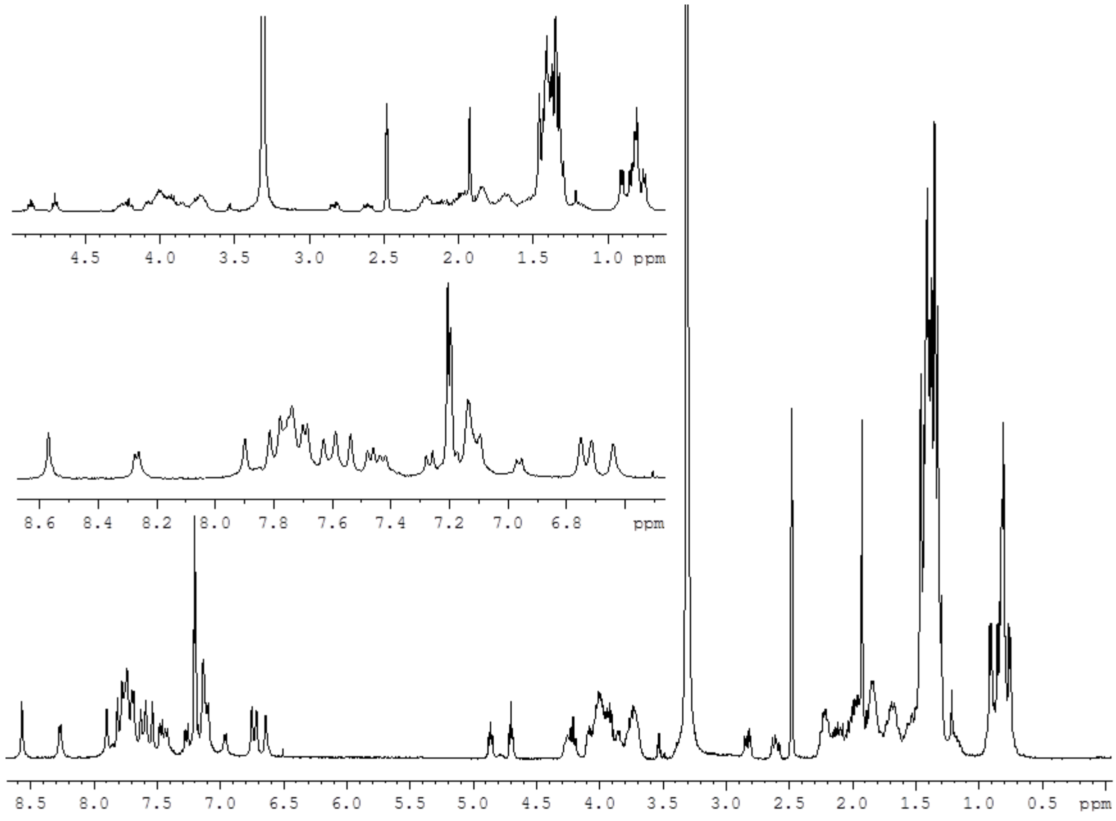

Figure S17. ^{13}C spectrum of TA1909 (**2**).

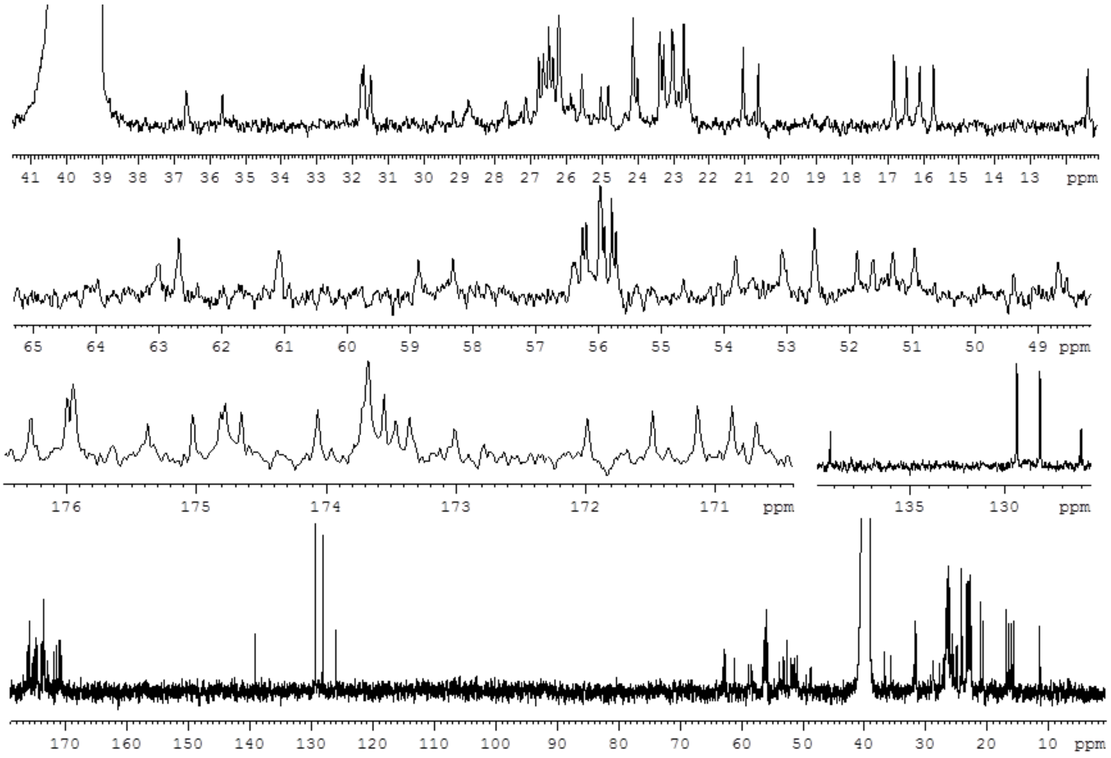

Figure S18. ^{1}H spectrum of TA1895 (**3**).

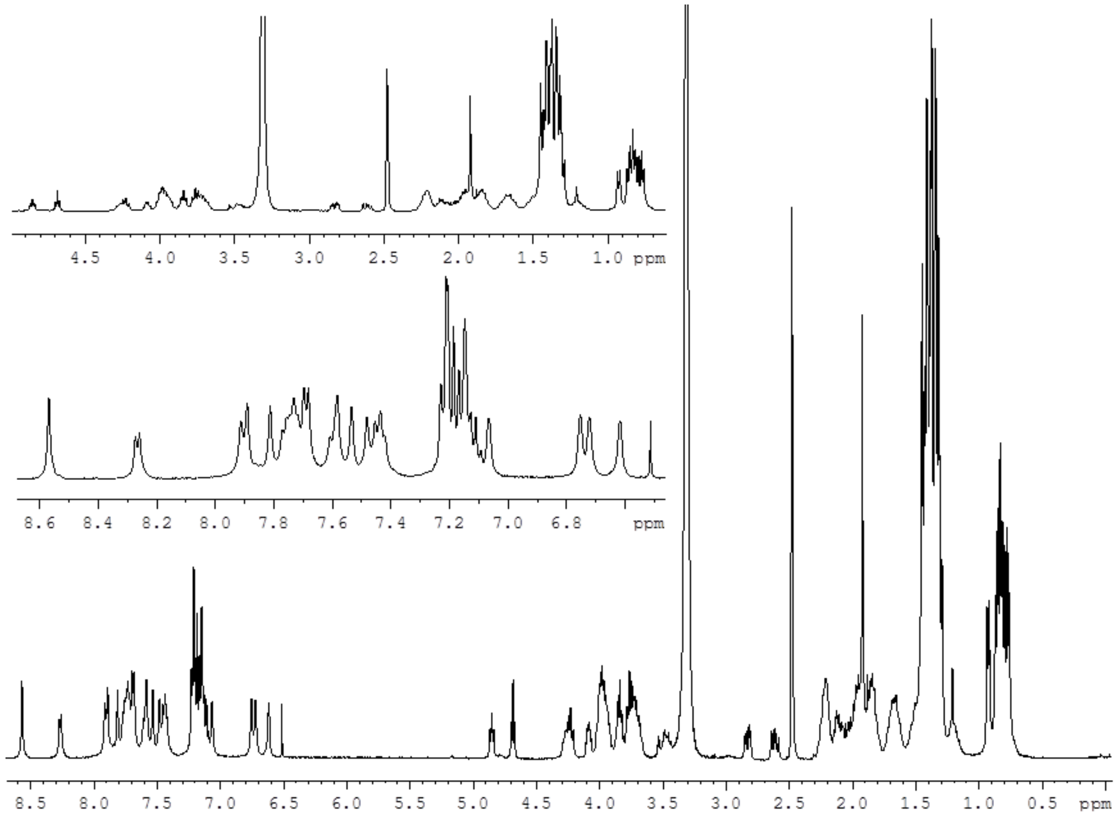

Figure S19. ^{13}C spectrum of TA1895 (**3**).

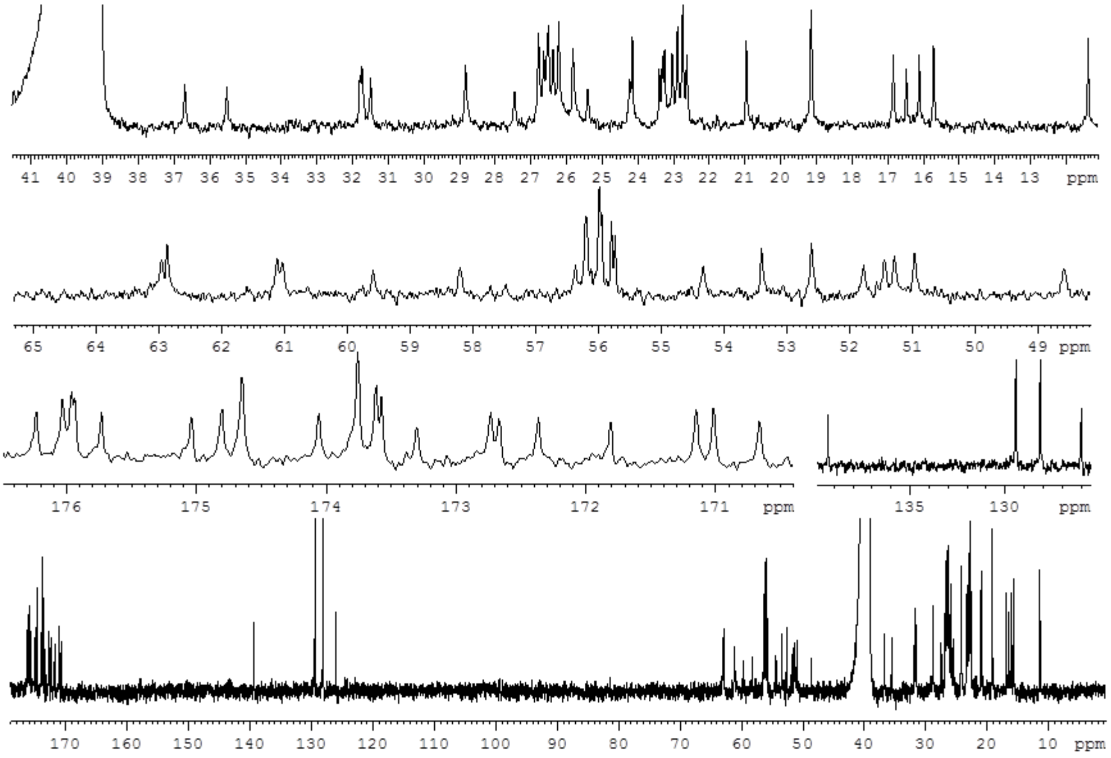

Figure S20. ^{1}H spectrum of TA1896 (**4**).

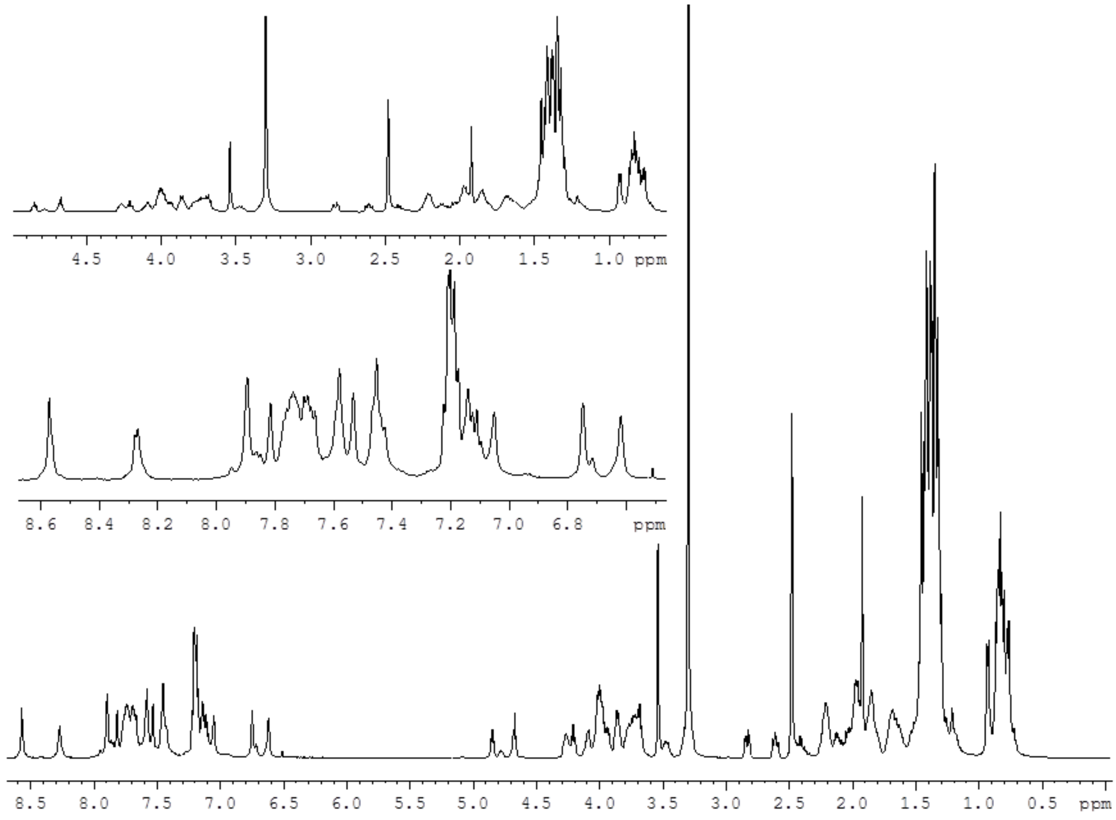

Figure S21. ^{13}C spectrum of TA1896 (**4**).

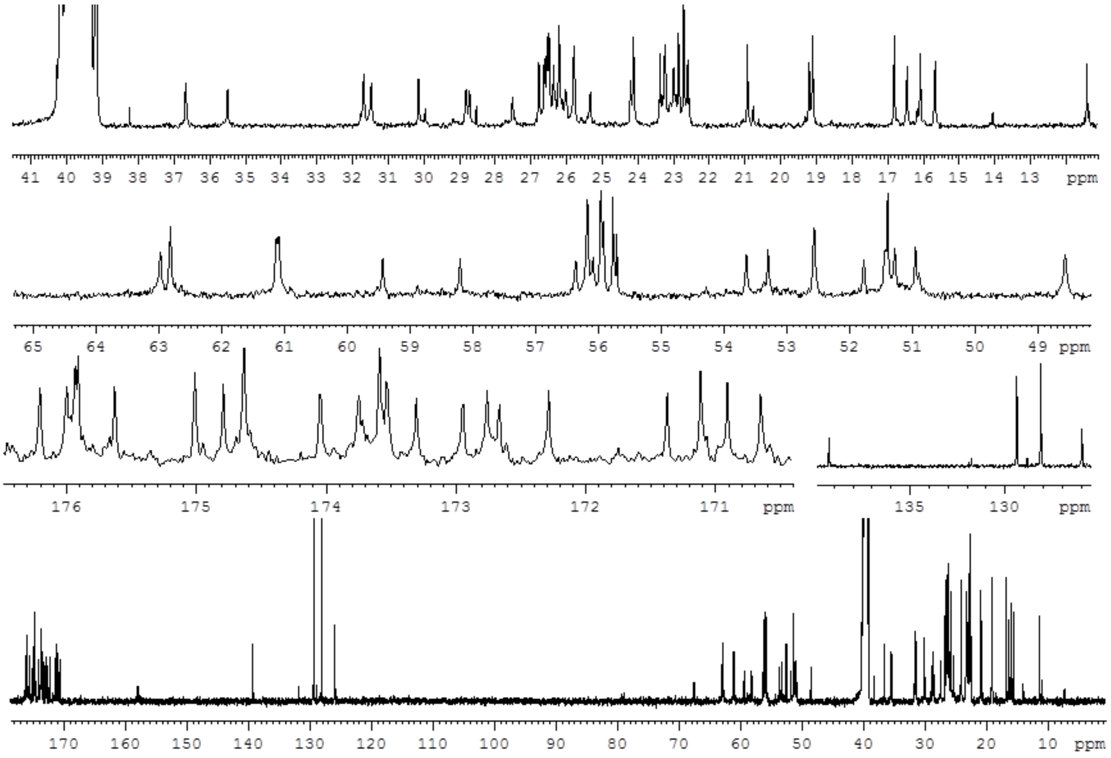

Figure S22. ^{1}H spectrum of TA1924 (**5**).

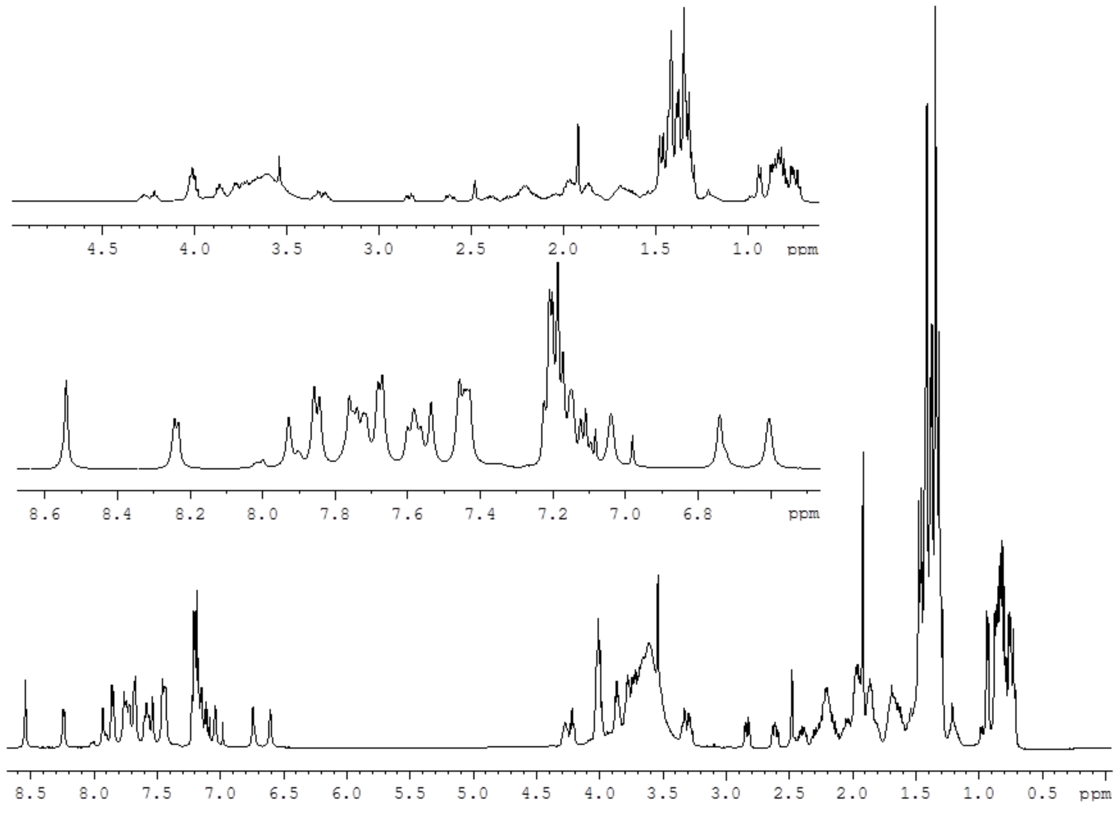

Figure 23. ^{13}C spectrum of TA1924 (**5**).

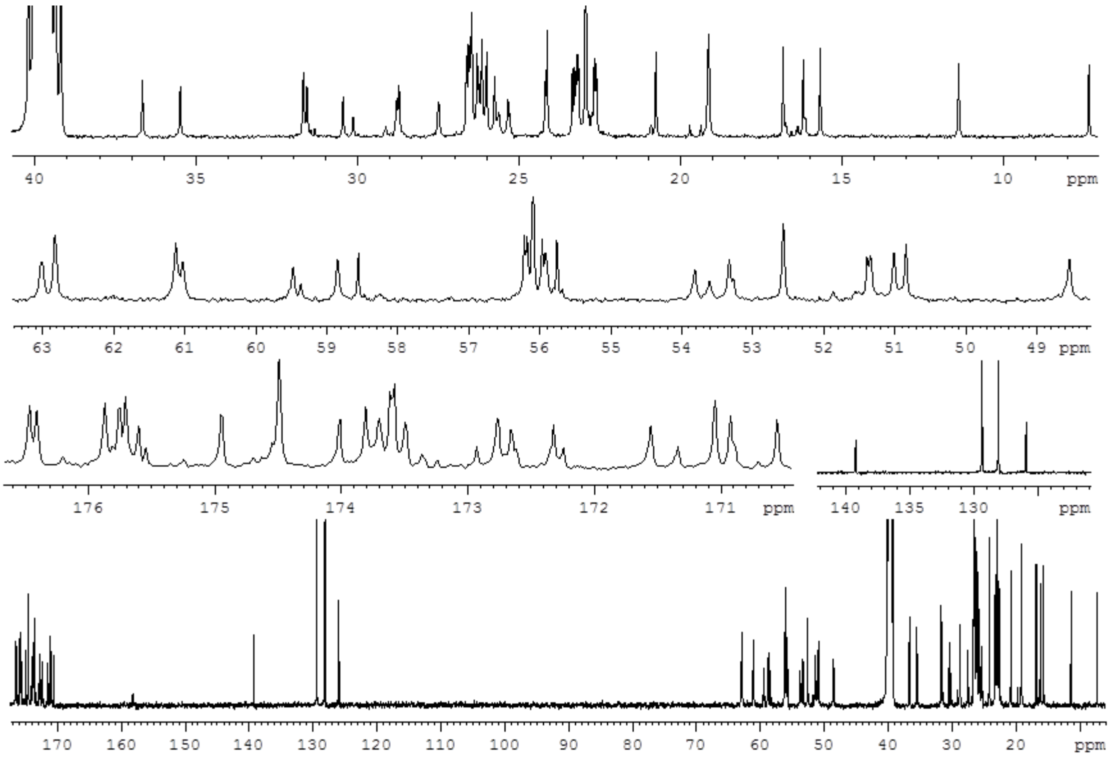

Figure S24. ^{1}H spectrum of TA1910 (**6**).

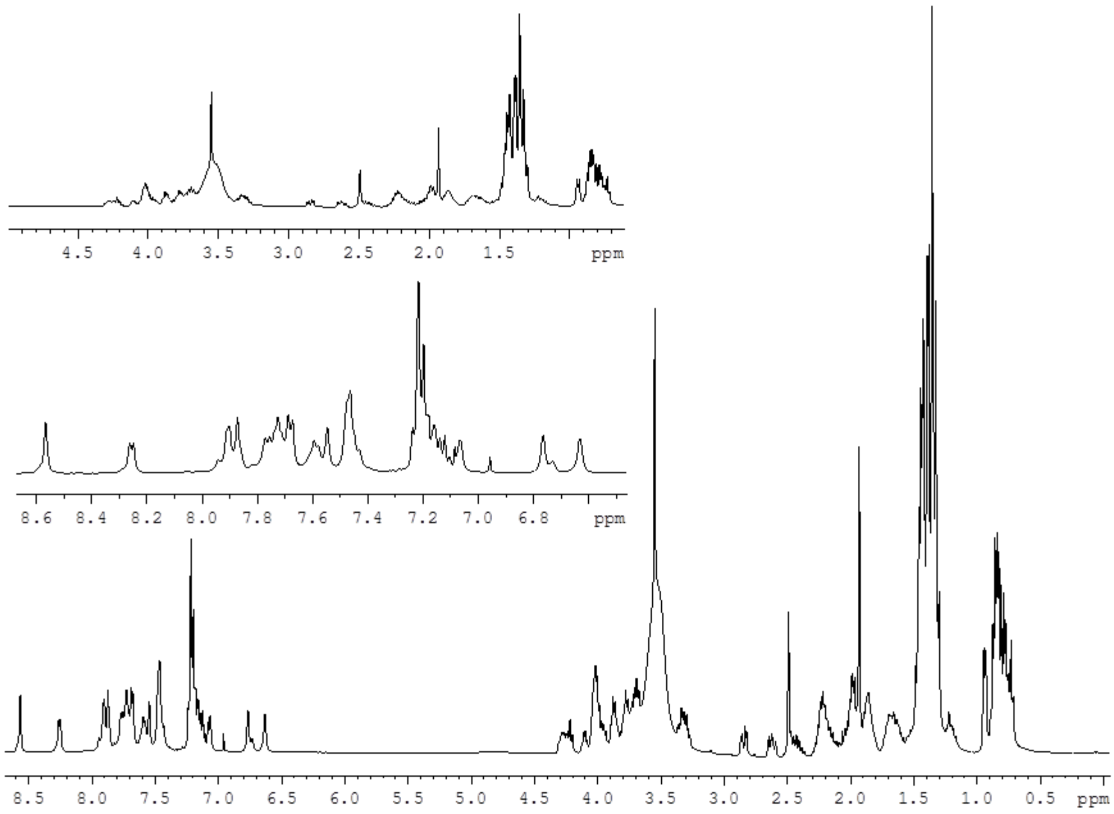

Figure S25. ^{13}C spectrum of TA1910 (**6**).

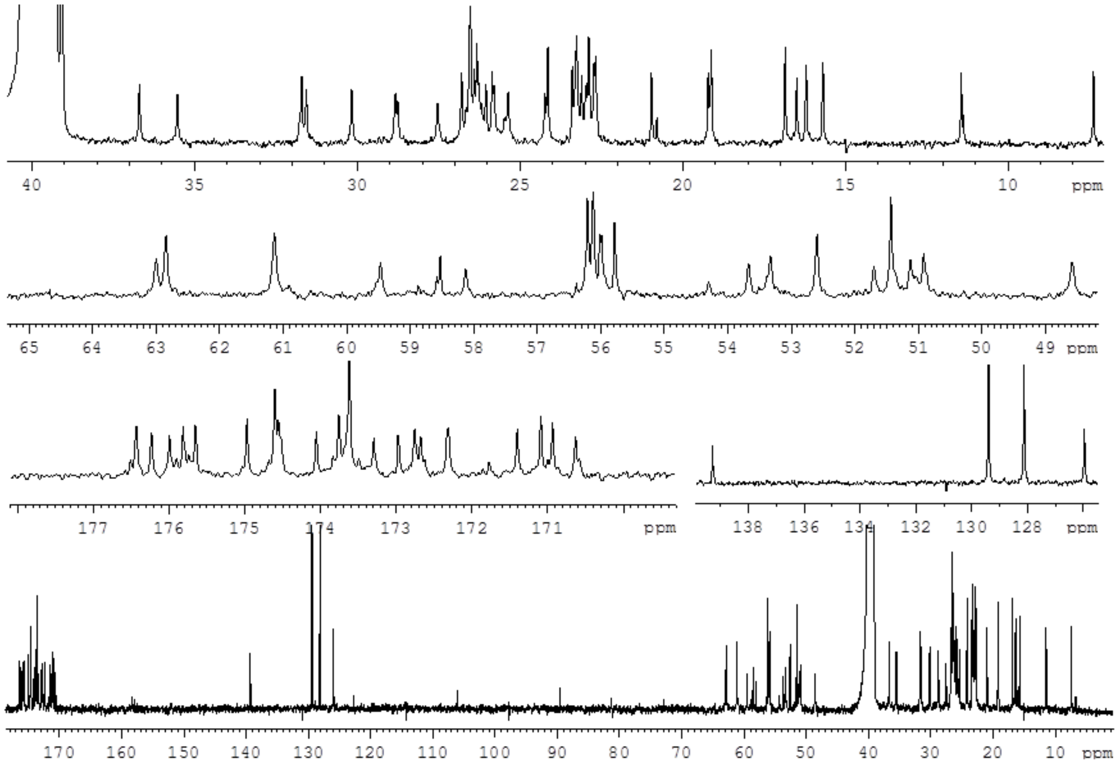

Figure S26. ^{1}H spectrum of TA1924A (**7**).

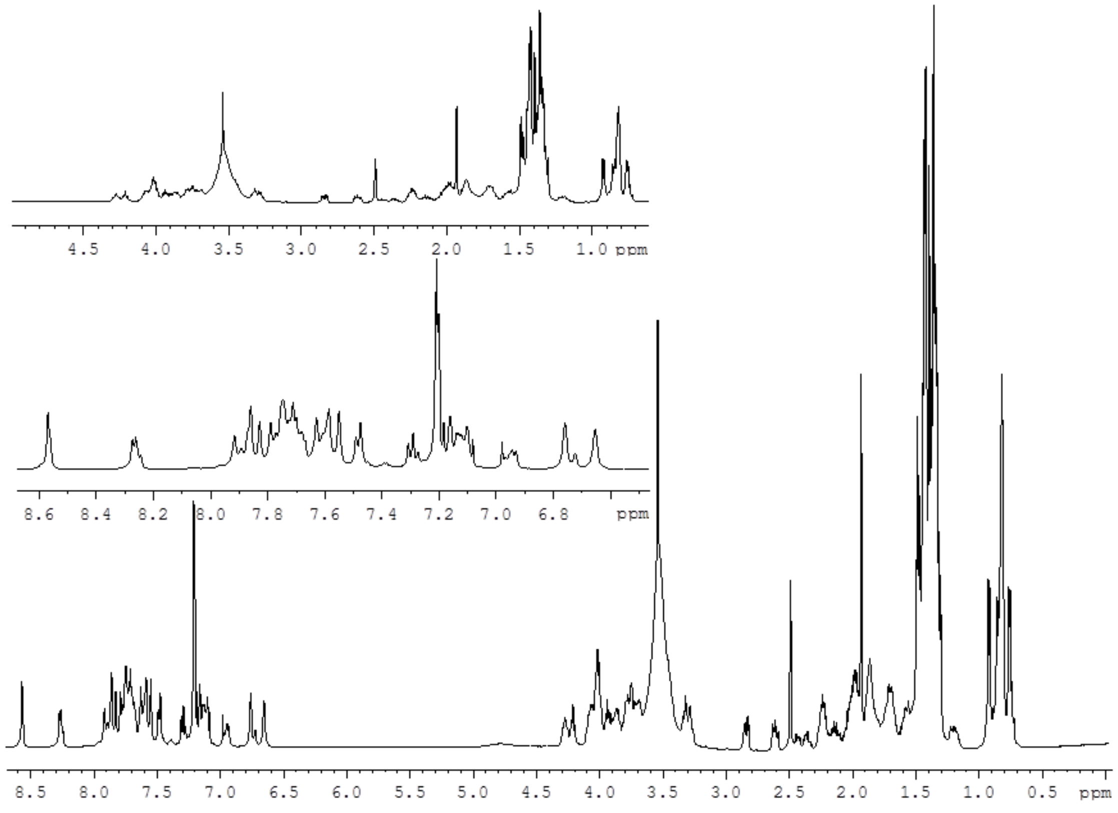

Figure S27. ^{13}C spectrum of TA1924A (**7**).

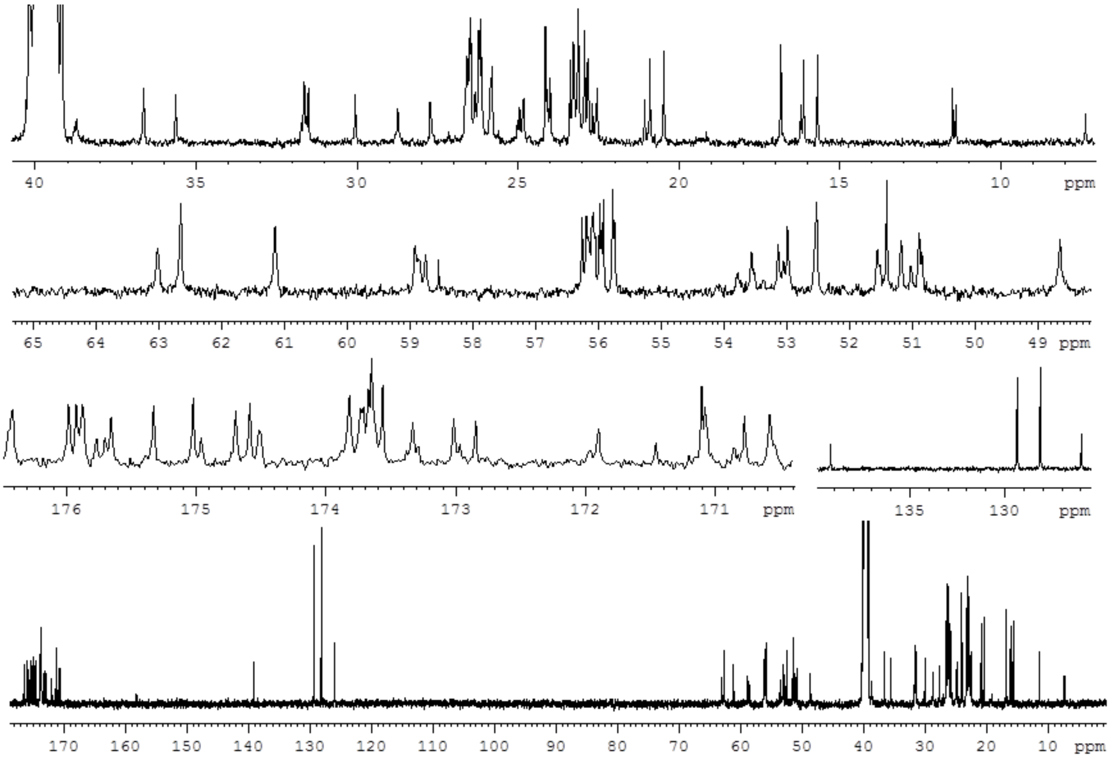

Figure S28. ^{1}H spectrum of TA1909A (**8**).

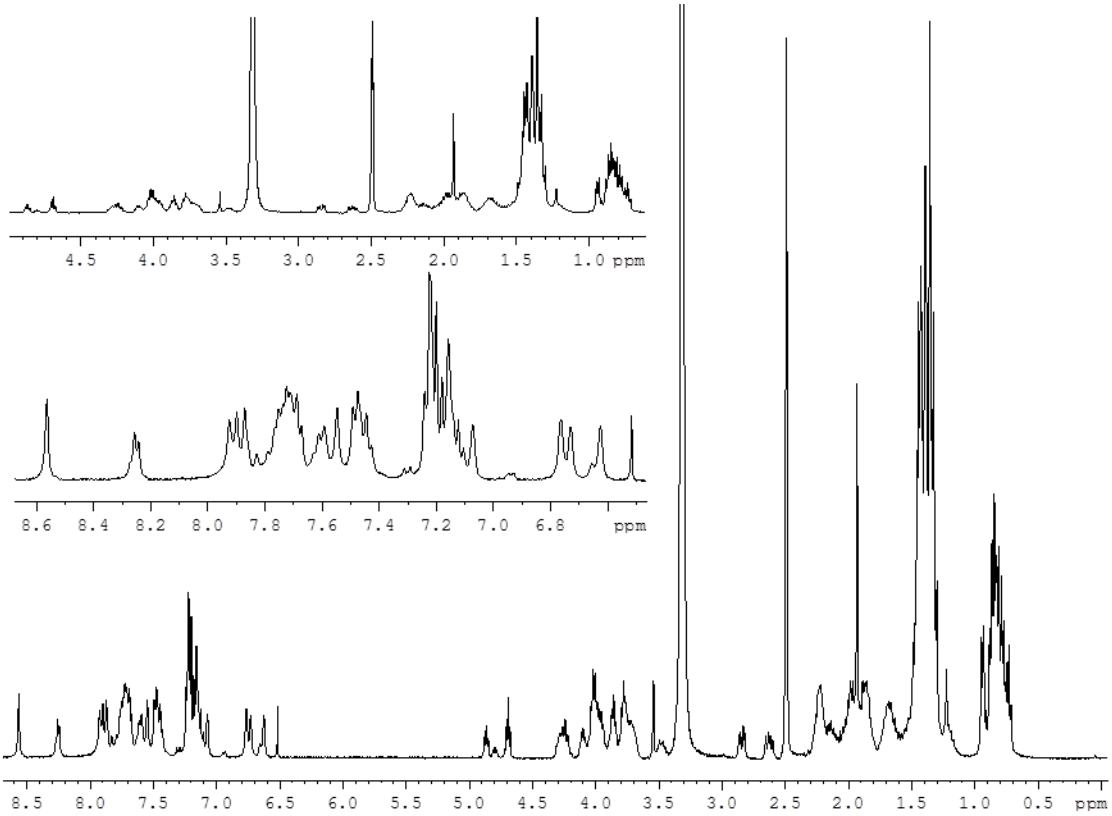

Figure S29. ^{13}C spectrum of TA1909A (**8**).

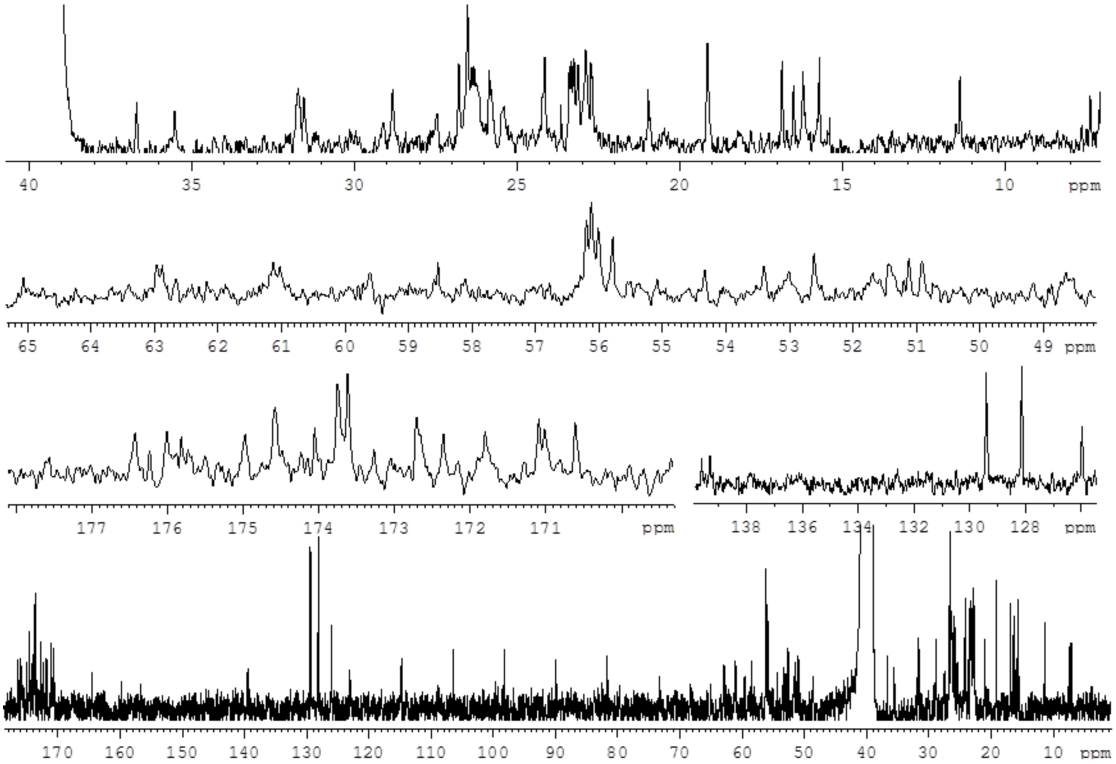

Figure S30. Structure of TA1909 (**2**) presenting key HMBC (⌒) and ROESY (⌒) correlations.

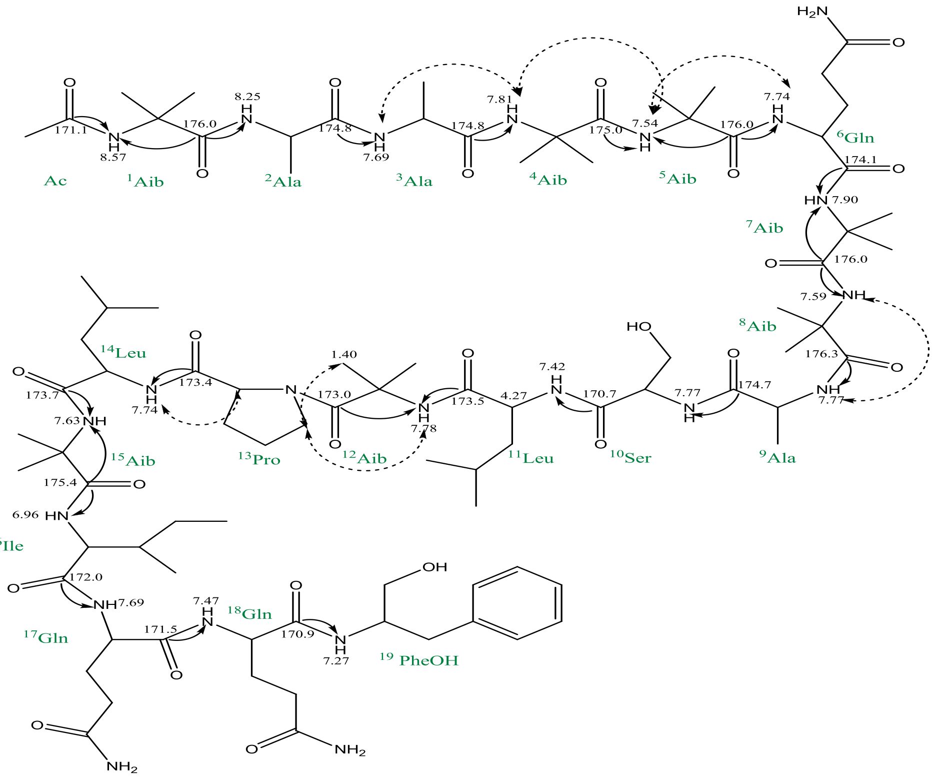

Figure S31. Structure of TA1895 (**3**) presenting key HMBC (⌒) and ROESY (⋰⋱) correlations.

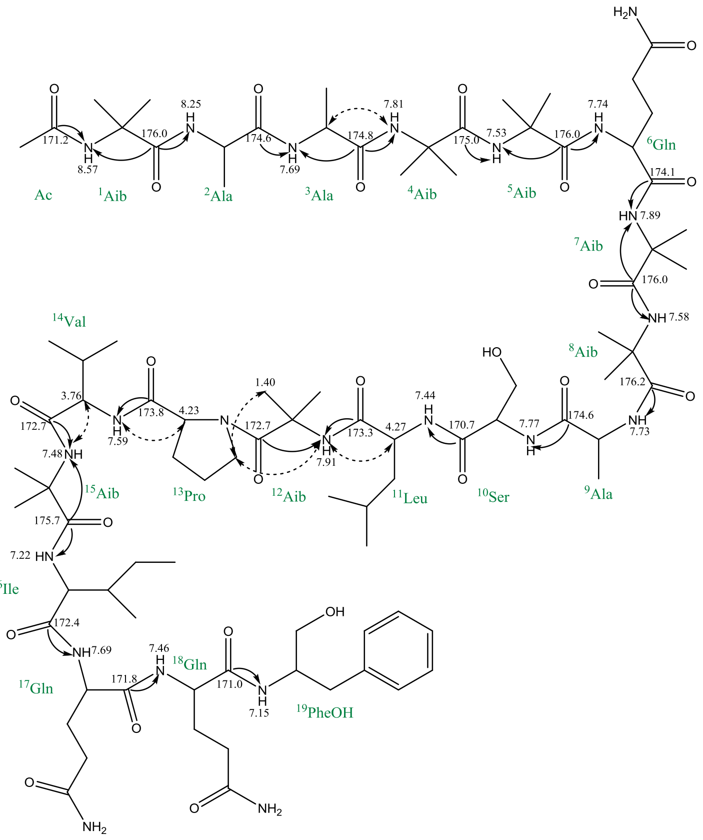

Figure S32. Structure of TA1896 (**4**) presenting key HMBC (⌒) and ROESY (⋰⋱) correlations.

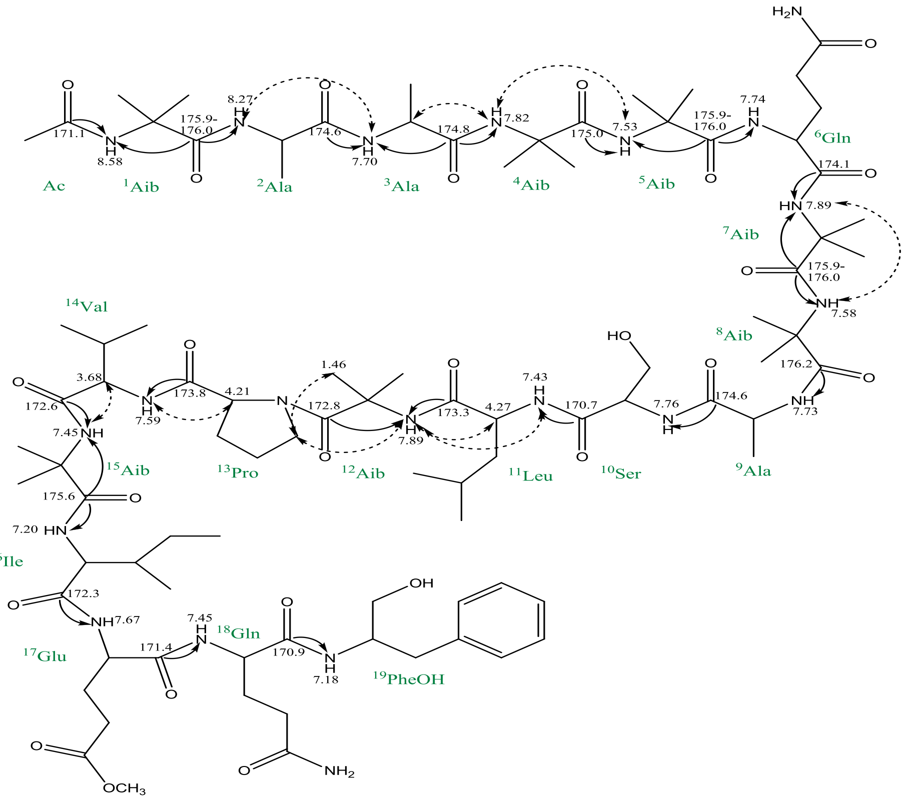

Figure S33. Structure of TA1924 (**5**) presenting key HMBC ( ) and ROESY ( ) correlations.

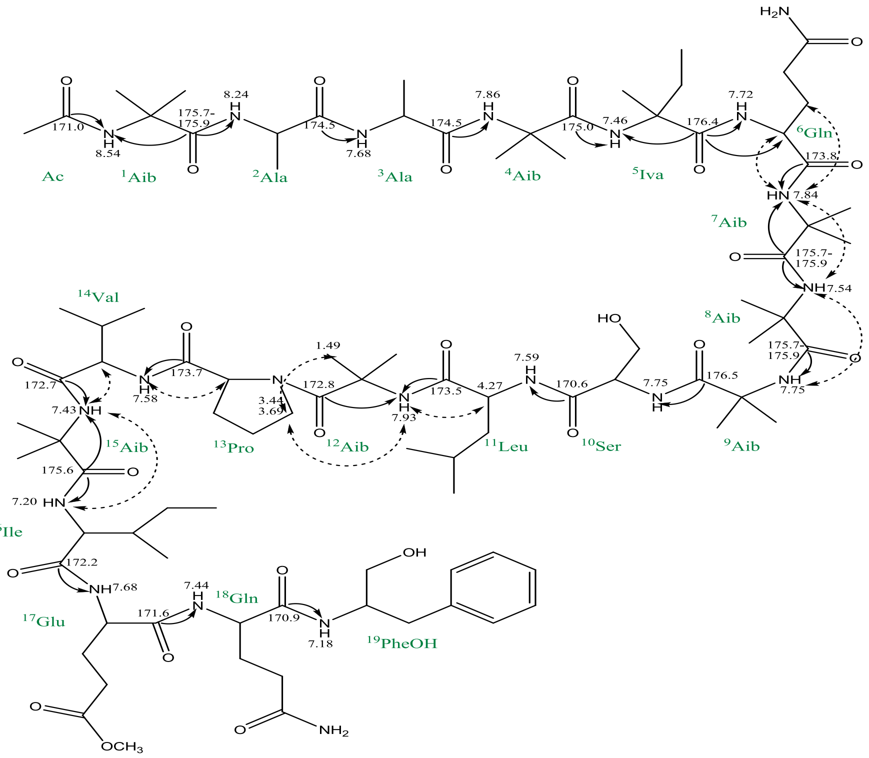

Figure S34. Structure of TA1910 (6) presenting key HMBC ( ) and ROESY ( ) correlations.

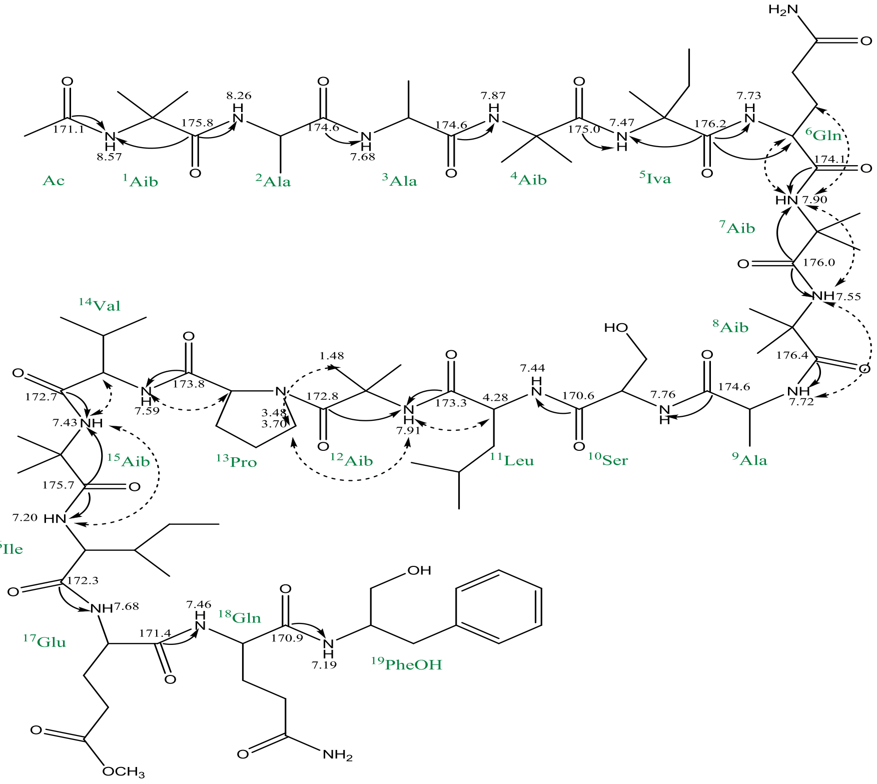

Figure S35. Structure of TA1924a (**7**) presenting key HMBC (⌒→) and ROESY (⌒) correlations.

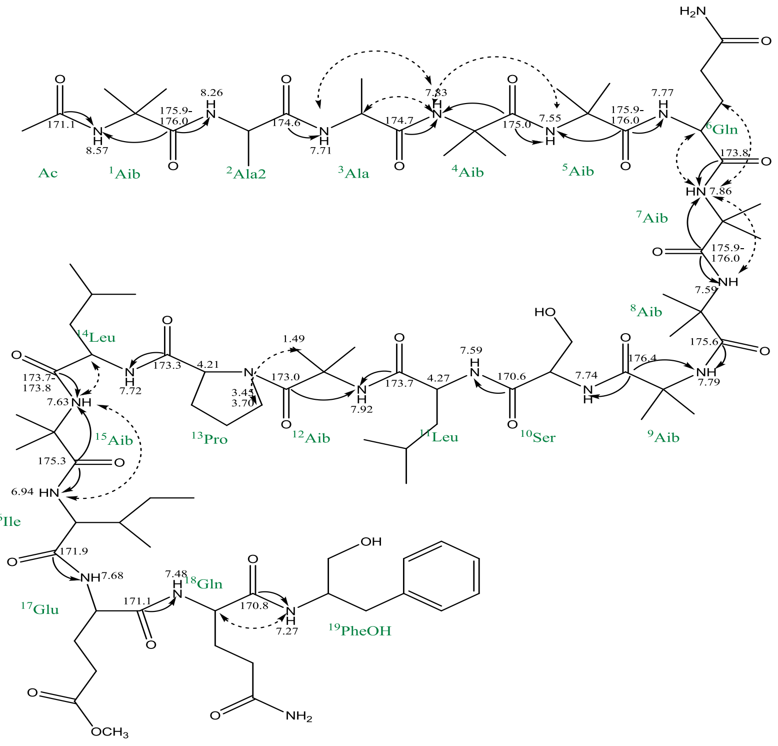

Figure S36. Structure of TA1909a (**8**) presenting key HMBC (⌒→) and ROESY (⌒) correlations.

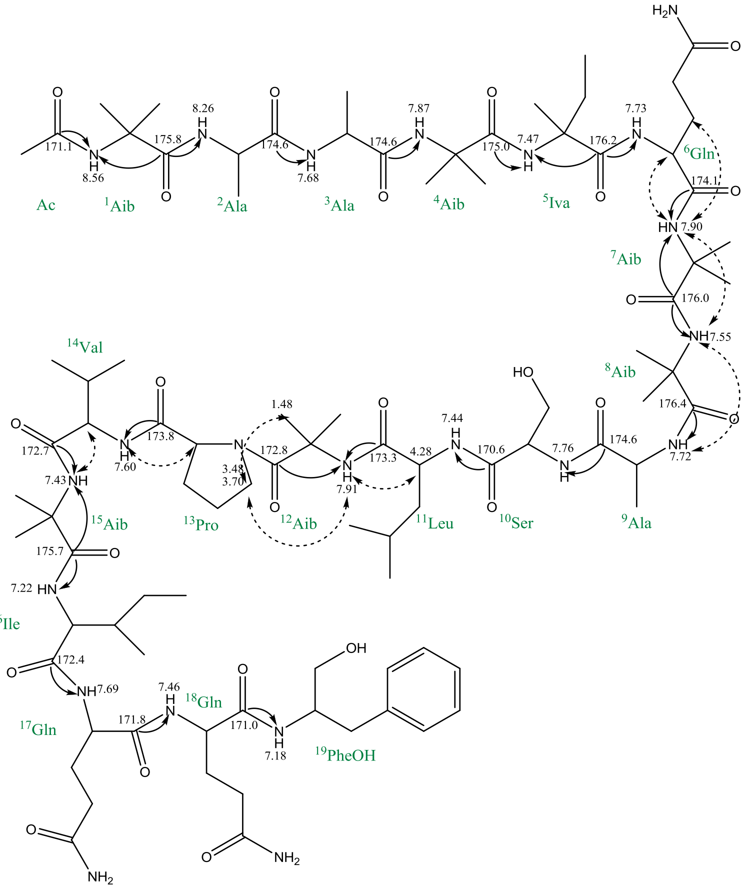

Figure S37. Structure of TA.VIb (**9**) presenting key HMBC (⁀) and ROESY (⋯) correlations.

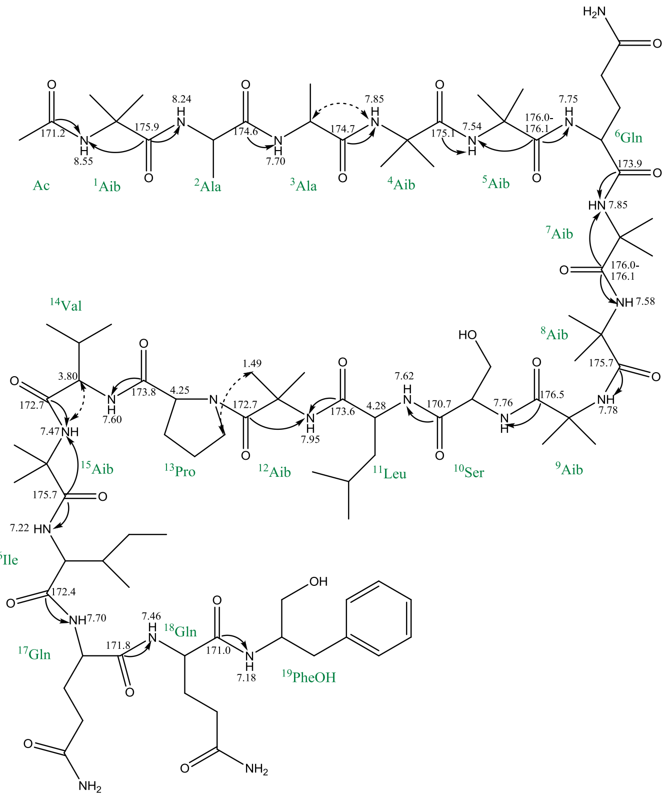

Figure S38. Structure of TA.VIa (**10**) presenting key HMBC (⁀) and ROESY (⋯) correlations.

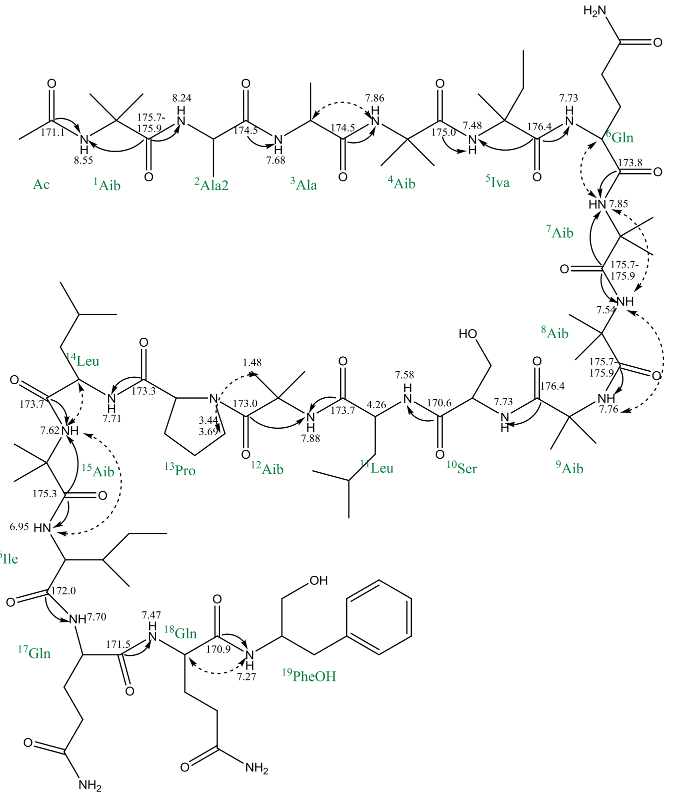

Figure S39. Structure of TA.VII (**11**) presenting key HMBC (⌒) and ROESY (⌒, dashed) correlations.

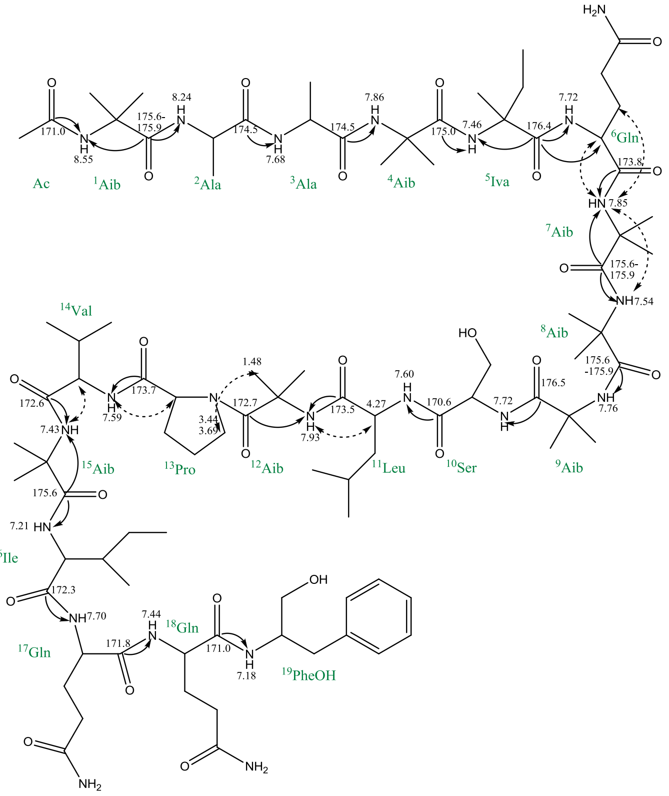

Figure S40. Structure of TA.Vb (**12**) presenting key HMBC (⌒) and ROESY (⌒, dashed) correlations.

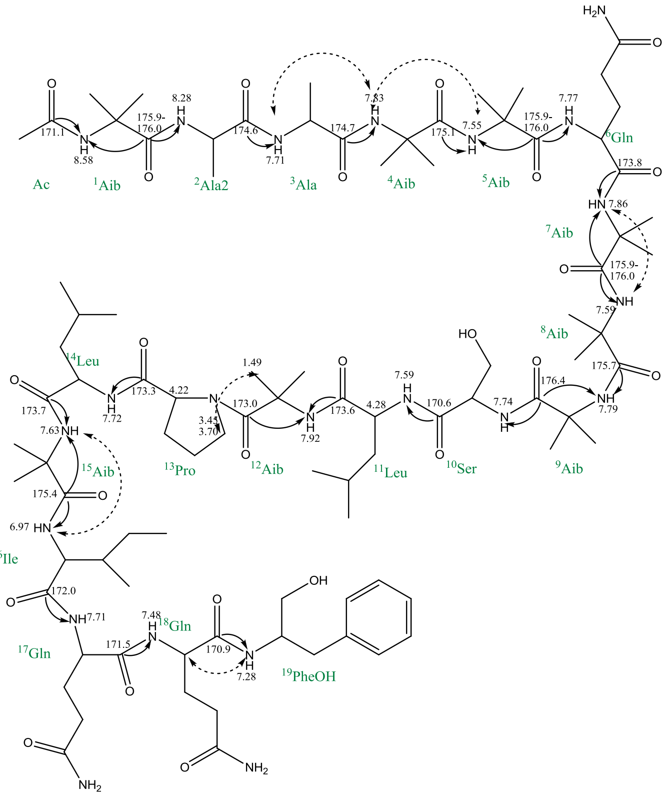

Table S1. Known trichorzianines [1–3]. Positions marked in grey differ between compounds.

Trichorzianines	1	2	3	4	5	6	7	8	9	10	11	12	13	14	15	16	17	18	19	MW (g/mol)
TA IIa	AcAib	Ala	Ala	Aib	Aib	Gln	Aib	Aib	Aib	Ser	Leu	Aib	Pro	Leu	Aib	Ile	Gln	Gln	TrpOH	1962.1
TA IIIa	AcAib	Ala	Ala	Aib	Iva	Gln	Aib	Aib	Aib	Ser	Leu	Aib	Pro	Leu	Aib	Ile	Gln	Gln	TrpOH	1976.2
TA IIIb	AcAib	Ala	Ala	Aib	Aib	Gln	Aib	Aib	Aib	Ser	Leu	Aib	Pro	Val	Aib	Leu	Gln	Gln	TrpOH	1948.1
TA IIIc	AcAib	Ala	Ala	Aib	Aib	Gln	Aib	Aib	Aib	Ser	Leu	Aib	Pro	Val	Aib	Ile	Gln	Gln	TrpOH	1948.1
TA IVb	AcAib	Ala	Ala	Aib	Iva	Gln	Aib	Aib	Aib	Ser	Leu	Aib	Pro	Val	Aib	Ile	Gln	Gln	TrpOH	1962.1
TA Vb	AcAib	Ala	Ala	Aib	Aib	Gln	Aib	Aib	Aib	Ser	Leu	Aib	Pro	Leu	Aib	Ile	Gln	Gln	PheOH	1923.1
TA VIa	AcAib	Ala	Ala	Aib	Iva	Gln	Aib	Aib	Aib	Ser	Leu	Aib	Pro	Leu	Aib	Ile	Gln	Gln	PheOH	1937.1
TA VIb	AcAib	Ala	Ala	Aib	Aib	Gln	Aib	Aib	Aib	Ser	Leu	Aib	Pro	Val	Aib	Ile	Gln	Gln	PheOH	1909.1
TA VII	AcAib	Ala	Ala	Aib	Iva	Gln	Aib	Aib	Aib	Ser	Leu	Aib	Pro	Val	Aib	Ile	Gln	Gln	PheOH	1923.1
TB IIa	AcAib	Ala	Ala	Aib	Aib	Gln	Aib	Aib	Aib	Ser	Leu	Aib	Pro	Leu	Aib	Ile	Gln	Glu	TrpOH	1963.1
TB IIIc	AcAib	Ala	Ala	Aib	Aib	Gln	Aib	Aib	Aib	Ser	Leu	Aib	Pro	Val	Aib	Ile	Gln	Glu	TrpOH	1949.1
TB IVb	AcAib	Ala	Ala	Aib	Iva	Gln	Aib	Aib	Aib	Ser	Leu	Aib	Pro	Val	Aib	Ile	Gln	Glu	TrpOH	1963.1
TB Vb	AcAib	Ala	Ala	Aib	Aib	Gln	Aib	Aib	Aib	Ser	Leu	Aib	Pro	Leu	Aib	Ile	Gln	Glu	PheOH	1924.1
TB VIa	AcAib	Ala	Ala	Aib	Iva	Gln	Aib	Aib	Aib	Ser	Leu	Aib	Pro	Leu	Aib	Ile	Gln	Glu	PheOH	1938.1
TB VIb	AcAib	Ala	Ala	Aib	Aib	Gln	Aib	Aib	Aib	Ser	Leu	Aib	Pro	Val	Aib	Ile	Gln	Glu	PheOH	1910.1

Table S2. NMR data for TA1909 [a] (**2**) in DMSO-d_6.

Position		δ_C, mult. [b]	δ_H, mult., *J* (Hz)	LR C-H correlations [c]	NOE correlations [d]
Ac	1	171.1 s		Ac-2, [1]Aib-NH	
	2	23.1 q	1.93 s		[1]Aib-NH,3,4
[1]Aib	1	176.0 [e] s		[2]Ala-NH, [1]Aib-NH,3,4	
	2	55.7 [f] s		[1]Aib-NH,3,4	
	3	26.5 [g] q	1.35 s	[1]Aib-NH	[1]Aib-NH
	4	24.1 q	1.32 s	[1]Aib-3	[1]Aib-NH
	NH		8.57 s		Ac-2, [1]Aib-3,4
[2]Ala	1	174.8 s		[3]Ala-NH, [2]Ala-2,3	
	2	51.0 d	3.99 m	[2]Ala-NH,3	[2]Ala-NH,3
	3	16.8 q	1.31 d	[2]Ala-NH,2	[2]Ala-NH,2
	NH		8.27 d (5.0)		[3]Ala-NH, [2]Ala-2,3
[3]Ala	1	174.8 s		[4]Aib-NH, [3]Ala-2,3	
	2	51.4 d	3.99 m	[3]Ala-NH,3	[3]Ala-NH,3, [4]Aib-NH
	3	16.1 q	1.33 d	[2]Ala-NH,2	[3]Ala-NH,2
	NH		7.69 d (6.4)		[4]Aib-NH, [2]Ala-NH, [3]Ala-2,3
[4]Aib	1	175.0 s		[5]Aib-NH,3	
	2	56.0 s		[4]Aib-NH,3,4	
	3	26.2q	1.41 [j] s	[4]Aib-NH,4	[4]Aib-NH
	4	23.3 [i] q	1.36 [k] s	[4]Aib-3	[4]Aib-NH
	NH		7.81 s		[4]Aib-2,3, [3]Ala-NH,2, [5]Aib-NH
[5]Aib	1	176.0 [e] s		[6]Gln-NH, [5]Aib-3,4,NH	
	2	55.8 [f] s		[5]Aib-NH,3,4	
	3	26.7 [h] q	1.44 s	[5]Aib-NH,4	[5]Aib-NH
	4	22.6 q	1.39 s	[5]Aib-3	[5]Aib-NH
	NH		7.54 s		[5]Aib-3,4, [4]Aib-NH, [6]Gln-NH
[6]Gln	1	174.1 s		[7]Aib-NH, [6]Gln-2,3	
	2	56.2 d	3.74 m	[6]Gln-NH,3,4a,4b	7Aib-NH, [6]Gln-NH,3,4a,4b
	3	26.2 t	1.98 m	[6]Gln-2,4a,4b	[6]Gln-2,NH, 7Aib-NH
	4a	31.5 [i] t	2.13 m	[6]Gln-3, NH_2b	[6]Gln-2,4b,NH, NH_2 a
	4b		2.23 m		[6]Gln-2,4a
	5	173.6 s		[6]Gln-3,4a,4b,NH_2a,NH_2b	
	NH_2a		7.13 s		[6]Gln-4a, NH_2 b
	NH_2b		6.75 s		[6]Gln-NH_2 a
	NH		7.74 m		[6]Gln-2,3,4a, 5Aib-NH
[7]Aib	1	176.0 [e] s		[8]Aib-NH, [7]Aib-NH,3	
	2	56.0 s		[7]Aib-NH,3,4	
	3	26.2 [g] q	1.41 [j] s		[7]Aib-NH
	4	23.0 q	1.34 [k] s	[7]Aib-NH,3	[7]Aib-NH
	NH		7.90 s		[6]Gln-2,3, [7]Aib-3,4, [8]Aib-NH
[8]Aib	1	176.3 s		[9]Aib-NH, [8]Aib-3,4	
	2	56.0 s		[8]Aib-NH,3,4	
	3	26.8 [h] q	1.46 s	[8]Aib-NH,4	[8]Aib-NH
	4	22.7 q	1.38 s	[8]Aib-3	[8]Aib-NH
	NH		7.59		[8]Aib-3,4, [7]Aib-NH, [9]Ala-NH

Table S2. *Cont.*

^{9}Ala	1	174.7 s		^{10}Ser-NH, ^{9}Ala-2	
	2	51.9 d	3.94 m	^{9}Ala-3	^{9}Ala-NH,3
	3	16.5 q	1.40 d	^{9}Ala-2	^{9}Ala-NH,2
	NH		7.73 m		^{9}Ala-2,3, ^{8}Aib-NH
^{10}Ser	1	170.7 s		^{11}Leu-NH, ^{10}Ser-2,3a	
	2	58.3 d	4.09 m	^{10}Ser-NH,OH	^{10}Ser-NH,3a,3b, ^{11}Leu-NH
	3a	61.1 t	3.72 m	^{10}Ser-NH,2,OH	^{10}Ser-NH,2,OH
	3b		3.77 m		^{10}Ser-NH,2,OH
	OH		4.86 t (6.0)		^{10}Ser-NH,3a,3b
	NH		7.77 m		^{10}Ser-2,3a,3b,OH
^{11}Leu	1	173.5 s		^{12}Aib-NH, ^{11}Leu-2	
	2	51.6 d	4.27 m	^{11}Leu-NH	^{11}Leu-NH,4,5,3a,3b, ^{14}Leu-5
	3a	39.5 t	1.54 m	^{11}Leu-2.5,6	^{11}Leu-2,5,3b
	3b		1.67 m		^{11}Leu-NH,2,3a,5
	4	24.0 d	1.73 m	^{11}Leu-5,6	^{11}Leu-NH,6
	5	20.6 q	0.76 d (6.0)	^{11}Leu-6,3b	^{11}Leu-2,3a,3b
	6	23.0 q	0.81 d	^{11}Leu-5,3b	^{11}Leu-4
	NH		7.43 d (7.9)		^{10}Ser-2, ^{11}Leu-2,3b,4
^{12}Aib	1	173.0 s		^{12}Aib-NH,3,4	
	2	55.9 s		^{12}Aib-NH,3,4	
	3	25.6 q	1.40 s	^{12}Aib-4	^{12}Aib-NH
	4	23.4 [i] q	1.46 s	^{12}Aib-NH,3	^{12}Aib-NH,^{13}Pro-5b
	NH		7.78 s		^{13}Pro-5b, ^{12}Aib-3,4
^{13}Pro	1	173.4 s		^{14}Leu-NH, ^{13}Pro-2,3a	
	2	63.0 d	4.21 t (8.1)	^{13}Pro-4,5b	^{14}Leu-NH,5, ^{13}Pro-4,3b
	3a	28.8 t	1.58 m	^{13}Pro-2,5b	^{13}Pro-3b
	3b		2.22 m		^{13}Pro-3a,2,5b,4
	4	25.9 t	1.84 m	^{13}Pro-2,3a,3b,5b	^{13}Pro-2,3b,5a,5b
	5a	48.7 t	3.38 m	^{13}Pro-3b	^{13}Pro-4,5b
	5b		3.71 m		^{13}Pro-4, 5a, ^{12}Aib-NH,3
^{14}Leu	1	173.7		^{15}Aib-NH, ^{14}Leu-2	
	2	53.1 d	3.94 m	^{14}Leu-4	^{14}Leu-4,3a,3b,6,NH, ^{15}Aib-NH
	3a	38.7 t	1.51 m	^{14}Leu-2,4,5,6	^{14}Leu-2,3b,6
	3b		1.78 m		^{14}Leu-2,3a,5,6,NH
	4	24.8 d	1.67 m	^{14}Leu-5	^{14}Leu-2, NH
	5	23.0 q	0.91 d (6.4)	^{14}Leu-3b,4,6	^{14}Leu-3b, ^{13}Pro-2, ^{11}Leu-2
	6	21.1 q	0.82 d	^{14}Leu-4,5	^{14}Leu-2,3a,3b
	NH		7.74 m		^{14}Leu-2,3b,4, ^{13}Pro-2
^{15}Aib	1	175.4 s		^{16}Ile-NH, ^{15}Aib-NH,3,4	
	2	56.3 s		^{15}Aib-NH,3,4	
	3	26.3 [g] q	1.42 [j] s	^{15}Aib-3,NH	^{15}Aib-NH
	4	23.4 [i] q	1.35 [k] s	^{15}Aib-4	^{15}Aib-NH
	NH		7.63 s		^{15}Aib-3,4, ^{16}Ile-NH, ^{14}Leu-2

Table S2. *Cont.*

^{16}Ile	1	172.0 s		^{17}Gln-NH, ^{16}Ile–2	
	2	58.9 d	3.91 m	^{16}Ile-6,4b	^{16}Ile-NH,5,6,4a,4b,3
	3	35.6 d	1.87 m	^{16}Ile-2,4a,4b,5,6,NH	^{16}Ile-NH, 2,6
	4a	25.0 t	1.20 m	^{16}Ile-2,3,5,6	^{16}Ile-NH,2,4b
	4b		1.47 m		^{16}Ile-NH,2,4a,5
	5	11.4 q	0.81 t	^{16}Ile-4a	^{16}Ile-4b,2
	6	15.7 q	0.85 d (6.8)	^{16}Ile-4a,2	^{16}Ile-NH,2,3
	NH		6.96 d (7.0)		^{16}Ile-2,3,4a,4b,6, ^{15}Aib-NH
^{17}Gln	1	171.5 s		^{18}Gln-NH, ^{17}Gln-2,3a,3b	
	2	53.8 d	4.03 m	^{17}Gln-NH,4a,4b	^{17}Gln-NH,3a,3b,4a,4b
	3a	27.1 t	1.85 m	^{17}Gln-2,4a,4b	^{17}Gln-NH,2
	3b		1.94 m		^{17}Gln-NH,2
	4a	31.7 [i] t	2.08 m	^{17}Gln-NH2a,2,3b	^{17}Gln-NH,2
	4b		2.18 m		^{17}Gln-NH,2,NH_2b
	5	173.7 s		^{17}Gln-4a,4b,NH_2a,NH_2b	
	NH_2a		6.72 (s)		^{17}Gln-NH_2b
	NH_2b		7.13 (s)		^{17}Gln-NH_2a,4b
	NH		7.69 d (6.4)		^{17}Gln-2,3a,3b,4a,4b
^{18}Gln	1	170.9 s		^{19}Pheol-NH, ^{18}Gln-2,3a,3b	
	2	53.1 d	4.04 m	^{18}Gln-NH,3b,4a,4b,NH	^{19}PheOH-NH, ^{18}Gln-NH,3a,3b,4a,4b
	3a	27.7	1.83 m	^{1}8Gln-2,4a,4b	^{18}Gln-NH,2,3b,4a
	3b		1.70 m		^{18}Gln-NH,2,3a,4b
	4a	31.7 [i] t	2.03 m	^{18}Gln-NH_2a,2,3a	^{18}Gln-2,3a
	4b		1.97 m		^{18}Gln-NH,NH_2b,2,3b
	5	173.7		^{18}Gln-NH_2a, NH_2b,3a,3b,4a,4b	
	NH_2a		6.64 s		^{18}Gln-NH_2b
	NH_2b		7.10 s		^{18}Gln-NH_2a,4b
	NH		7.47 d (7.9)		^{18}Gln-2,3a,3b,4b
^{19}Pheol	1a	62.7 t	3.28 m	^{19}Pheol-3a,3b,OH	^{19}Pheol-OH,NH
	1b		3.32 m		^{19}Pheol-OH,NH
	2	52.6 d	3.86 m	^{19}Pheol-1a,1b,3a,3b,NH,OH	^{19}Pheol-OH,NH,3a,3b,5
	3a	36.7 t	2.61 dd (13.7, 8.4)	^{19}Pheol-1a,1b,2,5,NH	^{19}Pheol-NH,2,3b,5
	3b		2.83 dd (13.6, 5.2)		^{19}Pheol-,2,3a,5,OH
	4	139.2 s		^{19}Pheol-2,3a,3b,5	
	5	129.4 d	7.20 m	^{19}Pheol-3a,3b,6,7	^{19}Pheol-2,3a,3b
	6	128.2 d	7.19 m	^{19}Pheol-5,6	
	7	126.0 d	7.11 m	^{19}Pheol-5,6	
	OH		4.70 t (5.7)		^{19}PheOH-1a,1b 2,3b,NH
	NH		7.27 d (8.6)		^{18}Gln-2, ^{19}Pheol-1a,1b,2,3a,OH

[a] Carried out on an AVANCE-400 Bruker instrument; [b] Multiplicity and assignment are from HSQC experiment; [c] Values determined from HMBC experiment; $^{n}J_{CH}$ = 8 Hz, recycle time 1 s; [d] Selected NOE's from a ROESY experiment; [e,f,g,h,i,j,k] These signals may interchange.

Table S3. NMR data for TA1895 [a] (**3**) in DMSO-d_6.

Position		δ_C, mult. [b]	δ_H, mult., *J* (Hz)	LR C-H correlations [c]	NOE correlations [d]
Ac	1	171.2 s		Ac-2, ^{1}Aib-NH	
	2	23.3 q	1.92 s		^{1}Aib-NH,3,4
^{1}Aib	1	176.0 s		^{2}Ala-NH, ^{1}Aib-NH,3,4	
	2	55.8 [f] s		^{1}Aib-NH,3,4	
	3	26.8 q	1.35 s	^{1}Aib-NH	^{1}Aib-NH
	4	24.2 q	1.32 s	^{1}Aib-3	^{1}Aib-NH
	NH		8.57 s		Ac-2, ^{1}Aib-3,4
^{2}Ala	1	174.6 s		^{3}Ala-NH, ^{2}Ala-2,3	
	2	51.0 d	3.98 m	^{2}Ala-NH,3	^{2}Ala-NH,3
	3	16.8 q	1.31 d	^{2}Ala-NH,2	^{2}Ala-NH,2
	NH		8.27 d (5.4)		^{3}Ala-NH, ^{2}Ala-2,3
^{3}Ala	1	174.8 s		^{4}Aib-NH, ^{3}Ala-2,3,NH	
	2	51.3 d	3.99 m	^{3}Ala-NH,3	^{3}Ala-NH,3, ^{4}Aib-NH
	3	16.1 q	1.33 d	^{2}Ala-NH,2	^{3}Ala-NH,2
	NH		7.69 d (5.6)		^{2}Ala-NH, ^{3}Ala-2,3
^{4}Aib	1	175.0 s		^{5}Aib-NH	
	2	56.0 s		^{4}Aib-NH,3,4	
	3	25.8 [j] q	1.39 [k] s	^{4}Aib-NH,4	^{4}Aib-NH
	4	22.6 [h] q	1.38 s	^{4}Aib-3	^{4}Aib-NH
	NH		7.81 s		^{4}Aib-2,3,^{3}Ala-2
^{5}Aib	1	176.0 s		^{6}Gln-NH, ^{5}Aib-3,4,NH	
	2	55.7 [f] s		^{5}Aib-NH,3,4	
	3	26.7 [g] s	1.42 [m] s	^{5}Aib-NH,4	^{5}Aib-NH
	4	22.8 [h] q	1.37 [l] s	^{5}Aib-3	^{5}Aib-NH
	NH		7.53 s		^{5}Aib-3,4
^{6}Gln	1	174.1 s		^{7}Aib-NH, ^{6}Gln-2	
	2	56.4 d	3.73 m	^{6}Gln-NH,3,4a,4b	^{7}Aib-NH, ^{6}Gln-NH,3,4a,4b
	3	26.5 t	1.99 m	^{6}Gln-2,4a,4b	^{6}Gln-2,NH, ^{7}Aib-NH
	4a	31.5 [i] t	2.13 m	^{6}Gln-2,3,NH$_2$b	^{6}Gln-2, NH, NH$_2$ a
	4b		2.23 m		^{6}Gln-NH,2, NH$_2$ a
	5	173.6 [e] s		^{6}Gln-3,4a,4b,NH$_2$a,NH$_2$b	
	NH$_2$a		7.13 s		^{6}Gln-4a,4b,NH$_2$b
	NH$_2$b		6.75 s		^{6}Gln-NH$_2$a
	NH		7.74 m		^{6}Gln-2,3,4a,4b
^{7}Aib	1	176.0 s		^{8}Aib-NH, ^{7}Aib-NH,3,4	
	2	56.0 s		^{7}Aib-NH,3,4	
	3	26.2 [j] q	1.41 [k] s	^{7}Aib-NH,4	^{7}Aib-NH
	4	23.0 [h] q	1.37 [l] s	^{7}Aib-NH	^{7}Aib-NH
	NH		7.89 s		^{6}Gln-2,3, ^{7}Aib-3,4
^{8}Aib	1	176.2 s		^{9}Ala-NH, ^{8}Aib-3,4,NH	
	2	56.0 s		^{8}Aib-NH,3,4	
	3	26.8 q	1.45 s	^{8}Aib-NH,4	^{8}Aib-NH
	4	23.2 [h] q	1.35 [l] s	^{8}Aib-3	^{8}Aib-NH
	NH		7.58 s		^{8}Aib-3,4

Table S3. *Cont.*

^{9}Ala	1	174.6 s		^{10}Ser-NH, ^{9}Ala-2,3	
	2	51.8 d	3.93 m	^{9}Ala-3	^{9}Ala-NH,3
	3	16.5 q	1.40 d	^{9}Ala-2,NH	^{9}Ala-NH,2
	NH		7.73 m		^{9}Ala-2,3
^{10}Ser	1	170.7 s		^{11}Leu-NH, ^{10}Ser-2,3a	
	2	58.2 d	4.09 m	^{10}Ser-OH	^{10}Ser-NH,3a,3b
	3a	61.1 t	3.72 m	^{10}Ser-NH,2,OH	^{10}Ser-NH,2,OH
	3b		3.77 m		^{10}Ser-NH,2,OH
	OH		4.85 t (6.1)		^{10}Ser-NH,3a,3b
	NH		7.77 m		^{10}Ser-2,3a,3b,OH
^{11}Leu	1	173.3 s		^{12}Aib-NH	
	2	51.5 d	4.27 m	^{11}Leu-NH	^{11}Leu-NH,4,5,3a,3b, ^{12}Aib-NH
	3a	39.5 t	1.52 m	^{11}Leu-5,6	^{11}Leu-2,6
	3b		1.65 m		^{11}Leu-NH,6
	4	24.2 d	1.68 m	^{11}Leu-5,6	^{11}Leu-NH,5,6
	5	21.0 q	0.77 d (6.4)	^{11}Leu-6,3b	^{11}Leu-2,4
	6	23.1 q	0.83 d (7.2)	^{11}Leu-5,3b	^{11}Leu-4,3a,3b
	NH		7.44 d		^{11}Leu-2,3b,4
^{12}Aib	1	172.7 s		^{12}Aib-NH,3,4	
	2	56.4 s		^{12}Aib-NH,3,4	
	3	26.3 [g] q	1.38 [m] s	^{12}Aib-NH	^{12}Aib-NH
	4	23.1 q	1.45 s	^{12}Aib-NH	^{12}Aib-NH, ^{13}Pro-5b
	NH		7.91 s		^{13}Pro-5b, ^{11}Leu-2, ^{12}Aib-3,4
^{13}Pro	1	173.8 s		^{14}Leu-NH, ^{13}Pro-2,3a	
	2	63.0 d	4.23 t (7.6)	^{13}Pro-4	^{14}Val-NH, ^{13}Pro-4,3b
	3a	28.8 t	1.64 m	^{13}Pro-2,5b	^{13}Pro-3b
	3b		2.22 m		^{13}Pro-3a,2,4
	4	25.8 t	1.84 m	^{13}Pro-5b	^{13}Pro-2,3b,5a
	5a	48.6 t	3.47 m	^{13}Pro-3a,3b	^{13}Pro-4,5b
	5b		3.69 m		^{13}Pro-5a, ^{12}Aib-NH,4
^{14}Val	1	172.7		^{15}Aib-NH, ^{14}Val-2	
	2	61.0 d	3.76 m	^{14}Val-3,4,5	^{14}Val-4,3,NH, ^{15}Aib-NH
	3	28.8 d	2.22 m	^{14}Val-2,4,5	^{14}Val-2,4,5
	4	19.1 q	0.93 d (6.4)	^{14}Val-5,2,3	^{14}Val-NH,2,3
	5	19.1 q	0.86 d (8.0)	^{14}Val-4,2,3	^{14}Val-2,3
	NH		7.59 m		^{14}Val-2,3,4, ^{13}Pro-2
^{15}Aib	1	175.7 s		^{16}Ile-NH, ^{15}Aib-NH,3,4	
	2	56.4 s		^{15}Aib-NH,3,4	
	3	26.4 [g] q	1.43 s	^{15}Aib-NH,3	^{15}Aib-NH
	4	23.4 [h] q	1.37 [l] s	^{15}Aib-4	^{15}Aib-NH
	NH		7.48 s		^{15}Aib-3,4, ^{14}Val-2

Table S3. *Cont.*

^{16}Ile	1	172.4 s		^{17}Gln-NH, ^{16}Ile-2,3	
	2	59.6 d	3.84 t	^{16}Ile-NH,6	^{16}Ile-NH,6,3, ^{17}Gln-NH
	3	35.5 d	1.88 m	^{16}Ile-2,4a,5,6	^{16}Ile-NH,2,6
	4a	25.4 t	1.20 m	^{16}Ile-2,5,6	^{16}Ile-NH,4b
	4b		1.47 m		^{16}Ile-NH,4a
	5	11.4 q	0.81 t	^{16}Ile-4a	
	6	15.7 q	0.85 d (7.2)	^{16}Ile-4a,2	^{16}Ile-2,3
	NH		7.22 m		^{16}Ile-2,3,4a,4b
^{17}Gln	1	171.8 s		^{18}Gln-NH, ^{17}Gln-2	
	2	54.3 d	3.95 m	^{17}Gln-NH,4a,4b	^{17}Gln-NH,3,4a,4b
	3	26.8 t	1.90 m	^{17}Gln-2,4a,4b	^{17}Gln-NH,2
	4a	31.8 [i] t	2.13 m	^{17}Gln-NH$_2$a,2,3	^{17}Gln-NH,2, NH$_2$b
	4b		2.23 m		^{17}Gln-NH,2, NH$_2$b
	5	173.7 [e] s		^{17}Gln-3,4a,4b,NH$_2$a,NH$_2$b	
	NH$_2$a		6.72 (s)		^{17}Gln-NH$_2$b
	NH$_2$b		7.13 (s)		^{17}Gln-NH$_2$a,4a,4b
	NH		7.69 d (5.6)		^{17}Gln-2,3,4a,4b, ^{16}Ile-2
^{18}Gln	1	171.0 s		^{19}Pheol-NH, ^{18}Gln-2	
	2	53.4 d	4.00 m	^{18}Gln-4a,4b	^{19}Pheol-NH, ^{18}Gln-NH,3a,4a,4b
	3a	27.5 d	1.81 m	^{18}Gln-2,4a,4b	^{18}Gln-2,3b,4a,4b
	3b		1.70 m		^{18}Gln-NH, 3a,4a,4b
	4a	31.7 [i] t	2.04 m	^{18}Gln-NH$_2$a,2	^{18}Gln-2,3a,3b,NH$_2$b,NH
	4b		1.97 m		^{18}Gln-NH,2,3a,3b
	5	173.8 [e] s		^{18}Gln-NH$_2$a,NH$_2$b,4a,4b	
	NH$_2$a		6.62 s		^{18}Gln-NH$_2$b
	NH$_2$b		7.07 s		^{18}Gln-NH$_2$a,4a
	NH		7.44 d		^{18}Gln-2,3b,4a,4b
^{19}Pheol	1a	62.9 t	3.30 m	^{19}Pheol-3a,3b,OH	
	1b		3.32 m		
	2	52.6 d	3.84 t (6.0)	^{19}Pheol-1a,1b,3a,3b,NH,OH	^{19}Pheol-OH,NH,3b,5
	3a	36.7 t	2.61 dd (13.5, 8.4)	^{19}Pheol-1a,1b,2,5	^{19}Pheol-NH,3b,5
	3b		2.83 dd (13.5, 5.1)		^{19}Pheol-NH,2,3a
	4	139.3 s		^{19}Pheol-3a,3b, 6	
	5	129.4 d	7.22 m	^{19}Pheol-3a,3b,5,7	^{19}Pheol-2,3a
	6	128.1 d	7.18 m	^{19}Pheol-6	
	7	126.0 d	7.11 m	^{19}Pheol-5	
	OH		4.69 t (5.9)		^{19}PheOH-2,NH
	NH		7.15 m		^{18}Gln-2, ^{19}Pheol-2,3a,3b,OH

[a] Carried out on an AVANCE-400, Bruker instrument; [b] Multiplicity and assignment are from HSQC experiment; [c] Values determined from HMBC experiment; $^{n}J_{CH}$ = 8 Hz, recycle time 1 s; [d] Selected NOE's from a ROESY experiment; [e,f,g,h,i,j,k,l,m] These signals may interchange.

Table S4. NMR data for TA1896 [a] (**4**) in DMSO-d_6.

Position		δ_C, mult. [b]	δ_H, mult., *J* (Hz)	LR C-H correlations [c]	NOE correlations [d]
Ac	1	171.1s		Ac-2, [1]Aib-NH	
	2	23.2 q	1.92 s		[1]Aib-NH
[1]Aib	1	175.9 [e] s		[2]Ala-NH, [1]Aib-NH,3,4	
	2	55.8 s		[1]Aib-NH,3,4	
	3	26.5 [g] q	1.35 s	[1]Aib-NH	[1]Aib-NH
	4	24.1 q	1.32 s	[1]Aib-3	[1]Aib-NH
	NH		8.58 s		Ac-2, [1]Aib-3,4, [2]Ala-NH
[2]Ala	1	174.6 s		[3]Ala-NH, [2]Ala-2,3	
	2	51.0 d	3.98 m	[2]Ala-NH,3	[2]Ala-NH,3
	3	16.8 q	1.31 d	[2]Ala-NH,2	[2]Ala-NH,2
	NH		8.27 d (5.0)		[3]Ala-NH, [2]Ala-2,3
[3]Ala	1	174.8 s		[4]Aib-NH, [3]Ala-2,3,NH	
	2	51.3 d	3.98 m	[3]Ala-NH,3	[3]Ala-NH,3, [4]Aib-NH
	3	16.1 q	1.33 d	[2]Ala-NH,2	[3]Ala-NH,2
	NH		7.70 d (6.0)		[2]Ala-NH, [3]Ala-2,3
[4]Aib	1	175.0 s		[5]Aib-NH	
	2	55.7 [f] s		[4]Aib-NH,3,4	
	3	26.2 [g] q	1.43 [i] s	[4]Aib-NH,4	[4]Aib-NH
	4	23.4 [h] q	1.38 [j] s	[4]Aib-3	[4]Aib-NH
	NH		7.82 s		[4]Aib-2,3, [3]Ala-2, [5]Aib-NH
[5]Aib	1	175.9 [e] s		[6]Gln-NH, [5]Aib-3,NH	
	2	55.8 [f] s		[5]Aib-NH,3,4	
	3	26.5 [g] q	1.43 [i] s	[5]Aib-4	[5]Aib-NH
	4	22.6 [h] q	1.35 s	[5]Aib-NH,3	[5]Aib-NH
	NH		7.53 s		[5]Aib-3,4, [4]Aib-NH
[6]Gln	1	174.1 s		[7]Aib-NH, [6]Gln-2	
	2	56.4 d	3.74 m	[6]Gln-NH,3,4a,4b	[7]Aib-NH, [6]Gln-NH,3,4a,4b
	3	26.2 t	1.99 m	[6]Gln-2,4a,4b,NH	[6]Gln-2,4a,4b,NH, [7]Aib-NH
	4a	31.5 t	2.13 m	[6]Gln-2,3,NH_2b	[6]Gln-2,3,NH,NH_2a
	4b		2.23 m		[6]Gln-NH,2,3,NH_2a
	5	173.6 s		[6]Gln-3,4a,4b,NH_2a,NH_2b	
	NH_2a		7.17 s		[6]Gln-4a,4b,NH_2b
	NH_2b		6.75 s		[6]Gln-NH_2a
	NH		7.74 m		[6]Gln-2,3,4a,4b
[7]Aib	1	176.0 [e] s		[8]Aib-NH, [7]Aib-NH,3,4	
	2	55.9 [f] s		[7]Aib-NH,3,4	
	3	26.5 [g] q	1.44 s	[7]Aib-NH,4	[7]Aib-NH
	4	22.7 [h] q	1.39 [j] s	[7]Aib-NH	[7]Aib-NH
	NH		7.89 s		[6]Gln-2,3, [7]Aib-3,4, [8]Aib-NH
[8]Aib	1	176.2 s		[9]Ala-NH, [8]Aib-3,4	
	2	56.0 [f] s		[8]Aib-NH,3,4	
	3	26.8 [g] q	1.46 s	[8]Aib-NH,4	[8]Aib-NH
	4	23.3 [h] q	1.39 [j] s	[8]Aib-3	[8]Aib-NH
	NH		7.58 s		[8]Aib-3,4, [7]Aib-NH

Table S4. *Cont.*

^{9}Ala	1	174.6 s		^{10}Ser-NH, ^{9}Ala-2,3	
	2	51.8 d	3.93 m	^{9}Ala-3	^{9}Ala-NH,3
	3	16.5 q	1.40 d	^{9}Ala-2,NH	^{9}Ala-NH,2
	NH		7.73 m		^{9}Ala-2,3
^{10}Ser	1	170.7 s		^{11}Leu-NH, ^{10}Ser-2,3a	
	2	58.2 d	4.09 m	^{10}Ser-OH,3b	^{10}Ser-NH,3a,3b
	3a	61.1 t	3.73 m	^{10}Ser-NH,2,OH	^{10}Ser-NH,2,OH
	3b		3.77 m		^{10}Ser-NH,2,OH
	OH		4.85 t (6.0)		^{10}Ser-NH, 3a,3b
	NH		7.76 m		^{10}Ser-2,3a,3b,OH, ^{11}Leu-NH
^{11}Leu	1	173.3 s		^{12}Aib-NH, ^{11}Leu-2	
	2	51.5 d	4.27 m	^{11}Leu-NH,3b	^{11}Leu-NH,4,5,3a,3b, ^{12}Aib-NH
	3a	39.5 t	1.53 m	^{11}Leu-5,6,4	^{11}Leu-2,5,6
	3b		1.66 m		^{11}Leu-NH,6,5
	4	24.2 d	1.69 m	^{11}Leu-5,6	^{11}Leu-NH,5,6
	5	20.8 q	0.77 d	^{11}Leu-6	^{11}Leu-2,4,3a,3b
	6	22.9 [h] q	0.83 d	^{11}Leu-5	^{11}Leu-4,3a,3b
	NH		7.43 m		^{11}Leu-2,3b,4, ^{12}Aib-NH, ^{10}Ser-NH
^{12}Aib	1	172.8 s		^{12}Aib-NH,3,4	
	2	56.1 [f] s		^{12}Aib-NH,3,4	
	3	26.4 [g] q	1.36 s	^{12}Aib-NH	^{12}Aib-NH, ^{13}Pro-5b
	4	23.2 [h] q	1.47 s	^{12}Aib-NH	^{12}Aib-NH
	NH		7.89 s		^{13}Pro-5a,5b, ^{11}Leu-2,NH, ^{12}Aib-3,4
^{13}Pro	1	173.8 s		^{14}Val-NH, ^{13}Pro-2,3a	
	2	63.0 d	4.21 t (7.5)	^{13}Pro-4,5b,5a	^{14}Val-NH, ^{13}Pro-4,3a,3b,5b
	3a	28.8 t	1.64 m	^{13}Pro-2,4,5b,5a	^{13}Pro-3b,2,5a
	3b		2.22 m		^{13}Pro-3a,2,4
	4	25.8 t	1.84 m	^{13}Pro-5b	^{13}Pro-2,3b,5a,5b
	5a	48.6 t	3.47 m	^{13}Pro-3a,3b,2,4	^{13}Pro-3a,4,5b, ^{12}Aib-NH
	5b		3.69 m		^{13}Pro-5a,2, ^{12}Aib-3,NH
^{14}Val	1	172.6		^{15}Aib-NH, ^{14}Val-2	
	2	61.1 d	3.68 m	^{14}Val-3,4,5,NH	^{14}Val-4,3,NH, ^{15}Aib-NH
	3	28.8 d	2.22 m	^{14}Val-2,4,5	^{14}Val-2,4,5,NH
	4	19.1 [k] q	0.93 d (6.5)	^{14}Val-5,2,3	^{14}Val-NH,2,3
	5	19.2 [k] q	0.86 d	^{14}Val-4,2,3	^{14}Val-2,3
	NH		7.59 m		^{14}Val-2,3,4, ^{13}Pro-2
^{15}Aib	1	175.6 s		^{16}Ile-NH, ^{15}Aib-NH,3,4	
	2	56.2 [f] s		^{15}Aib-NH,3,4	
	3	26.2 [g] q	1.42 [i] s	^{15}Aib-NH,4	^{15}Aib-NH
	4	22.9 [h] q	1.35 s	^{15}Aib-3	^{15}Aib-NH
	NH		7.45 s		^{15}Aib-3,4, ^{14}Val-2

Table S4. *Cont.*

^{16}Ile	1	172.3 s		^{17}Gln-NH, ^{16}Ile-2,3	
	2	59.5 d	3.87 t	^{16}Ile-NH,6,4a	^{16}Ile-NH,5,6,3,4a,4b, ^{17}Glu-NH
	3	35.5 d	1.88 m	^{16}Ile-2,4a,4b,5,6,NH	^{16}Ile-NH,2,6
	4a	25.3 t	1.20 m	^{16}Ile-2,5,6,3	^{16}Ile-NH,4b,2
	4b		1.47 m		^{16}Ile-NH,4a,2,5
	5	11.4 q	0.80 m	^{16}Ile-4a,4b	^{16}Ile-2,4a
	6	15.7 q	0.85 m	^{16}Ile-4a,4b,2	^{16}Ile-2,3,NH
	NH		7.20 m		^{16}Ile-2,3,4a,4b,6
^{17}Glu	1	171.4 s		^{18}Gln-NH, ^{17}Glu-2	
	2	53.7 d	4.01 m	^{17}Glu-NH,4a,4b,3	^{17}Glu-NH,3,4a
	3	26.0 t	1.98 m	^{17}Glu-2,4a,4b,NH	^{17}Glu-NH,2,4a,4b
	4a	30.2 t	2.41 m	^{17}Glu-2,3	^{17}Glu-NH,2,3
	4b		2.47 m		^{17}Glu-3
	5	173.0 s		^{17}Glu-4a,4b,OCH_3	
	NH		7.67 d (6.0)		^{17}Glu-2,3,4a, ^{18}Gln-NH
	OCH_3	51.4 q	3.55 s		
^{18}Gln	1	170.9 s		^{19}Pheol-NH, ^{18}Gln-2,3b,3a	
	2	53.3 d	4.01 m	^{18}Gln-3a,3b,4a,4b, NH	^{19}Pheol-NH, ^{18}Gln-NH,3a,3b,4a,4b
	3a	27.5 d	1.83 m	^{18}Gln-2,4a,4b	^{18}Gln-2,3b,4a,4b
	3b		1.70 m		^{18}Gln-NH,2,3a,4a,4b
	4a	31.7 t	2.05 m	^{18}Gln-NH_2a,2,3a,3b	1^{8}Gln-2,3a,3b,NH2b
	4b		1.97 m		^{18}Gln-NH, 2,3a,3b,NH_2b
	5	173.5 s		^{18}Gln-NH_2a,NH_2b, 4a,4b,3a,3b	
	NH_2a		6.51 s		^{18}Gln-NH_2b
	NH_2b		7.05 s		^{18}Gln-NH_2a,4a,4b
	NH		7.45 d		^{18}Gln-2,3b,4b
^{19}Pheol	1a	62.8 t	3.30 m	^{19}Pheol-2,3a,3b,OH	^{19}Pheol-OH,2,3a,3b
	1b		3.33 m		^{19}Pheol-OH,2,3a,3b
	2	52.6 d	3.86 m	^{19}Pheol-1a,1b,3a,3b,NH, OH	^{19}Pheol-OH,NH,3a,3b,5,1a,1b
	3a	36.7 t	2.61 dd (10.7, 6.7)	^{19}Pheol-1a,1b,2,5	^{19}Pheol-NH,3b,1a,b,2
	3b		2.83 dd (10.8,3.8)		^{19}Pheol-NH,5,2,3a,1a,1b,OH
	4	139.3 s		^{19}Pheol-2,3a,3b,5, 6	
	5	129.4 d	7.21 m	^{19}Pheol-3a,3b,5,7	^{19}Pheol-2,3b
	6	128.1 d	7.19 m	^{19}Pheol-6	
	7	125.9 d	7.11 m	^{19}Pheol-5	
	OH		4.67 t (5.8)		^{19}Pheol-2,3b,OH,1a,1b
	NH		7.18 m		^{19}PheOH-2,NH,3b,1a,1b

[a] Carried out on an AVANCE-500, Bruker instrument; [b] Multiplicity and assignment are from HSQC experiment; [c] Values determined from HMBC experiment; $^{n}J_{CH}$ = 8 Hz, recycle time 1 s; [d] Selected NOE's from a ROESY experiment; [e,f,g,h,i,j] These signals may interchange.

Table S5. NMR data for TA1924 [a] (**5**) in DMSO-d_6.

**Position**		**δ_C, mult.** [b]	**δ_H, mult., *J* (Hz)**	**LR C-H correlations** [c]	**NOE correlations** [d]
Ac	1	171.0 s		Ac-2, ^{1}Aib-NH	
	2	23.0 q	1.92 s		^{1}Aib-NH
^{1}Aib	1	175.6 [e] s		^{2}Ala-NH, ^{1}Aib-NH,3,4	
	2	55.8 s		^{1}Aib-NH,3,4	
	3	26.5 q	1.35 s	^{1}Aib-NH	^{1}Aib-NH
	4	24.1 q	1.32 s	^{1}Aib-3	^{1}Aib-NH
	NH		8.54 s		Ac-2, ^{1}Aib-3,4, ^{2}Ala-NH
^{2}Ala	1	174.5 s		^{3}Ala-NH, ^{2}Ala-2,3	
	2	50.8 d	4.00 m	^{2}Ala-NH,3	^{2}Ala-NH,3
	3	16.8 q	1.30 d	^{2}Ala-NH,2	^{2}Ala-NH,2
	NH		8.24 d (5.0)		^{2}Ala-2,3, ^{1}Aib-NH, ^{3}Ala-NH
^{3}Ala	1	174.5 s		^{4}Aib-NH, ^{3}Ala-2,3	
	2	51.0 d	4.02 m	^{3}Ala-NH,3	^{3}Ala-NH,3, ^{4}Aib-NH
	3	16.2 q	1.33 d (7.5)	^{2}Ala-NH,2	^{3}Ala-NH,2
	NH		7.68 d (5.5)		^{4}Aib-NH, ^{3}Ala-2,3, ^{2}Ala-NH
^{4}Aib	1	175.0 s		^{5}Iva-NH, ^{4}Aib-3	
	2	55.9 [f] s		^{4}Aib-NH,3,4	
	3	26.5q	1.43 s	^{4}Aib-NH,4	^{4}Aib-NH
	4	22.9 q	1.35 s	^{4}Aib-NH,3	^{4}Aib-NH
	NH		7.86 s		^{4}Aib-2,3, ^{3}Ala-NH,2
^{5}Iva	1	176.4 s		^{6}Gln-NH,2, ^{5}Iva-3a,3b,NH	
	2	58.6 s		^{5}Iva-NH,3a,3b,4,5	
	3a	25.6 t	2.20 m	^{5}Iva-4	^{5}Iva-NH,4,3b
	3b		1.62 m		^{5}Iva-4,3a
	4	7.4 q	0.73 t (7.5)	^{5}Iva-3a,3b	^{5}Iva-NH,3a,3b
	5	22.7 [h] q	1.35 [i] s	^{5}Iva-NH,3a,3b	^{5}Iva-NH,
	NH		7.46 s		^{5}Iva-4,5,3a
^{6}Gln	1	173.8 s		^{7}Aib-NH, ^{6}Gln-2	
	2	56.0 d	3.78 m	^{6}Gln-NH,3,4a,4b	^{7}Aib-NH,^{6}Gln-NH,3,4a,4b
	3	26.2 t	1.95 m	^{6}Gln-2,4a,4b,NH	^{6}Gln-2,4a,4b,NH, ^{7}Aib-NH
	4a	31.7 [j] t	2.14 m	^{6}Gln-3,NH_2b	^{6}Gln-NH_2a,2,3,4b,NH
	4b		2.23 m		^{6}Gln-NH_2a,2,3,4a,NH
	5	173.8 s		^{6}Gln-3,4a,4b,NH_2a,NH_2b	
	NH_2a		7.16 s		^{6}Gln-4a,NH_2b,4a,4b
	NH_2b		6.74 s		^{6}Gln-NH_2a
	NH		7.72 m		^{6}Gln-2,3,4a,4b
^{7}Aib	1	175.7 [e] s		^{8}Aib-NH, ^{7}Aib-NH,3,4	
	2	55.9 [f] s		^{7}Aib-NH,3,4	
	3	26.2 q	1.42 [j] s	^{7}Aib-NH	^{7}Aib-NH
	4	22.6 [h] q	1.35 [i] s	^{7}Aib-NH	^{7}Aib-NH
	NH		7.84 s		^{6}Gln-2,3, ^{7}Aib-3,4, ^{8}Aib-NH

Table S5. *Cont.*

^{8}Aib	1	175.9 [e] s		^{9}Aib-NH, ^{8}Aib-3,4	
	2	56.1 [f] s		^{8}Aib-NH,3,4	
	3	26.0 [g] q	1.42 [j] s	^{8}Aib-NH	^{8}Aib-NH
	4	22.8 [h] q	1.38 [i] s	^{8}Aib-NH	^{8}Aib-NH
	NH		7.54 s		^{8}Aib-3,4, ^{7}Aib-NH, ^{9}Aib-NH
^{9}Aib	1	176.5 s		^{10}Ser-NH, ^{9}Aib-3,4	
	2	56.0 [f] s		^{9}Aib-NH,3,4	
	3	26.2 [g] q	1.46 s	^{9}Aib-NH	
	4	23.0 [h] q	1.42 s	^{9}Aib-NH	^{9}Aib-NH
	NH		7.75 s		^{9}Aib-4, ^{8}Aib-NH
^{10}Ser	1	170.6 s		^{11}Leu-NH, ^{10}Ser-2,3a	
	2	58.8 d	4.02 m	^{10}Ser-NH,3b	^{10}Ser-NH,3a,3b, ^{11}Leu-NH
	3a	61.0 t	3.76 m	^{10}Ser-NH,2	^{10}Ser-NH,2
	3b		3.78 m		^{10}Ser-NH,2
	OH				
	NH		7.73 m		^{10}Ser-2,3a,3b
^{11}Leu	1	173.5 s		^{12}Aib-NH, ^{11}Leu-2	
	2	51.4 d	4.28 m	^{11}Leu-NH,4	^{12}Aib-NH, ^{11}Leu-NH,5,3a,3b,4, ^{13}Pro-5a, ^{14}Val-4
	3a	39.8 t	1.55 m	11Leu-2.4,5,6	^{11}Leu-2,5
	3b		1.68 m		^{11}Leu-NH,2,5,6
	4	24.1 d	1.69 m	^{11}Leu-5,6	^{11}Leu-NH,2
	5	20.8 q	0.76 d (6.0)	^{11}Leu-6,3b	^{11}Leu-2,3a,3b
	6	23.4 q	0.83 d	^{11}Leu-5,3a	^{11}Leu-3b
	NH		7.59 d (8.0)		^{10}Ser-2, ^{11}Leu-2,3b,4
^{12}Aib	1	172.8 s		^{12}Aib-NH,3,4	
	2	56.1 [f] s		^{12}Aib-NH,3,4	
	3	26.4 [g] q	1.38 s	^{12}Aib-NH	^{12}Aib-NH
	4	22.6 q	1.48 s	^{12}Aib-NH	^{12}Aib-NH, ^{13}Pro-5b,2
	NH		7.93 s		^{13}Pro-5a,5b, ^{11}Leu-2, ^{12}Aib-3,4
^{13}Pro	1	173.7 s		^{14}Val-NH, ^{13}Pro-2,3a	
	2	63.0 d	4.22 t (7.5)	^{13}Pro-3a,3b,4,5b	^{14}Val-NH, ^{13}Pro-4,3a,3b,5b, ^{12}Aib-3
	3a	28.7 t	1.65 m	^{13}Pro-2,5a,5b	^{13}Pro-2,3b,5a
	3b		2.22 m		^{13}Pro-3a,2,5b,4
	4	26.0 t	1.86 m	^{13}Pro-2,3a,5a,5b	^{13}Pro-2,3b,5a,5b
	5a	48.5 t	3.52 m	^{13}Pro-3a,3b,2,4	^{13}Pro-3a,4,5b, ^{11}Leu-2, ^{14}Val-NH, ^{12}Aib-NH
	5b		3.67 m		^{13}Pro-2,3b,4,5a, ^{12}Aib-NH,3
^{14}Val	1	172.7 s		^{15}Aib-NH, ^{14}Val-2	
	2	61.1 d	3.72 m	^{14}Val-3,4,5,NH	^{14}Val-4,5,3,NH, ^{15}Aib-NH
	3	28.8 d	2.21 m	^{14}Val-2,4,5,NH	^{14}Val-2,4,5,NH
	4	19.1 q	0.93 d (6.5)	^{14}Val-5,2,3	^{14}Val-NH,2,3, ^{11}Leu-2
	5	19.2 q	0.87 d (6.0)	^{14}Val-4,2,3	^{14}Val-2,3
	NH		7.58 m		^{14}Val-2,3,4, ^{13}Pro-2

Table S5. *Cont.*

^{15}Aib	1	175.6 s		^{16}Ile-NH,2, ^{15}Aib-NH,3,4	
	2	55.9 [f] s		^{15}Aib-NH,3,4	
	3	26.6 [g] q	1.42 [j] s	^{15}Aib-NH	^{15}Aib-NH
	4	23.3 [h] q	1.35 [i] s	^{15}Aib-NH	^{15}Aib-NH
	NH		7.43 s		^{15}Aib-3,4, ^{14}Val-2
^{16}Ile	1	172.2 s		^{17}Glu-NH, ^{16}Ile-2	
	2	59.5 d	3.88 m	^{16}Ile-NH,6,4a,4b,3	^{16}Ile-NH,6,4a,4b,3
	3	35.5 d	1.88 m	^{16}Ile-2,4a,4b,5,6,NH	^{16}Ile-NH,2,6
	4a	25.4 t	1.20 m	^{16}Ile-2,3,5,6	^{16}Ile-2,4b,NH
	4b		1.50 m		^{16}Ile-NH,2,4a,5
	5	11.4 q	0.80 t (7.0)	^{16}Ile-4a,4b,3	^{16}Ile-4b
	6	15.7 q	0.84 d	^{16}Ile-4a,4b,3,2	^{16}Ile-NH,2,3
	NH		7.20 m		^{16}Ile-2,3,4a,4b,6
^{17}Glu	1	171.6 s		^{18}Gln-NH, ^{17}Glu-2	
	2	53.8 d	4.01 m	^{17}Glu-NH,4a,4b	^{18}Gln-NH, ^{17}Glu-NH,3,4a,4b
	3	26.3 t	1.95 m	^{17}Glu-2,4a,4b	^{17}Glu-NH,2,4a,4b
	4a	30.5 t	2.40 m	^{17}Glu-2,3	^{17}Glu-NH,2,3,4b
	4b		2.30 m		^{17}Glu-NH,2,3,4a
	5	173.0 s		^{17}Glu-4a,4b,OCH$_3$	
	NH		7.68 d (5.5)		^{17}Glu-2,3,4a,4b, ^{18}Gln-NH
	OCH$_3$	51.4 q	3.55 s		
^{18}Gln	1	170.9 s		^{19}Pheol-NH, ^{18}Gln-2,3a,3b	
	2	53.3 d	4.03 m	^{18}Gln-NH,3a,3b,4a,4b	^{19}Pheol-NH, ^{18}Gln-NH,3a,3b,4b
	3a	27.5	1.81 m	^{18}Gln-2,4a,4b,NH	^{18}Gln-NH,2,3b,4a
	3b		1.70 m		^{18}Gln-2,3a,4a
	4a	31.6 [j] t	2.05 m	^{18}Gln-NH$_2$a,2,3a,3b	^{18}Gln-3a,3b
	4b		1.97 m		^{18}Gln-NH,NH$_2$b,2
	5	173.6 s		^{18}Gln-NH$_2$a,NH$_2$b,3a, 3b,4a,4b	
	NH$_2$a		6.61 s		^{18}Gln-NH$_2$b
	NH$_2$b		7.04 s		^{18}Gln-NH$_2$a,4b
	NH		7.44 d		^{19}Pheol-NH, ^{18}Gln-2,3b,4b, ^{17}Glu-NH,2
^{19}Pheol	1a	62.8 t	3.30 m	^{19}Pheol-2,3a,3b	^{19}Pheol-NH,2,3a,3b
	1b		3.33 m		^{19}Pheol-NH,2,3a,3b
	2	52.6 d	3.86 m	^{19}Pheol-1a,1b,3a,3b,NH	^{19}Pheol-NH,1a,1b,3a,3b,5
	3a	36.7 t	2.61 dd (10.6, 6.9)	^{19}Pheol-1a,1b,2,5	^{19}Pheol-NH,1a,1b,2
	3b		2.84 dd (10.8, 4.0)		^{19}Pheol-1a,1b,2
	4	139.3 s		^{19}Pheol-2,3a,3b,6	
	5	129.4 d	7.21 m	^{19}Pheol-3a,3b,5,7	^{19}Pheol-2,3b,7
	6	128.1 d	7.19 m	^{19}Pheol-6	
	7	126.0 d	7.11 m	^{19}Pheol-5	^{19}Pheol-5
	OH				
	NH		7.18 m		^{18}Gln-NH,2, ^{19}Pheol-1a,1b,2,3a

[a] Carried out on an AVANCE-500, Bruker instrument; [b] Multiplicity and assignment are from HSQC experiment; [c] Values determined from HMBC experiment; $^{n}J_{CH}$ = 8 Hz, recycle time 1 s; [d] Selected NOE's from a ROESY experiment; [e,f,g,h,i,j] These signals may interchange.

Table S6. NMR data for TA1910 [a] (**6**) in DMSO-d_6.

Position		δ_C, mult. [b]	δ_H, mult., *J* (Hz)	LR C-H correlations [c]	NOE correlations [d]
Ac	1	171.1 s		Ac-2, [1]Aib-NH	
	2	23.0 q	1.92 s		[1]Aib-NH
[1]Aib	1	175.8 s		[2]Ala-NH, [1]Aib-NH,3,4	
	2	55.8 s		[1]Aib-NH,3,4	
	3	26.5 q	1.35 s	[1]Aib-NH	[1]Aib-NH
	4	24.1 q	1.33 s	[1]Aib-3	[1]Aib-NH
	NH		8.57 s		Ac-2, [1]Aib-3,4, [2]Ala-NH
[2]Ala	1	174.6 s		[3]Ala-NH, [2]Ala-2,3	
	2	50.9 d	4.01 m	[2]Ala-NH,3	[2]Ala-NH,3
	3	16.9 q	1.31 d	[2]Ala-NH,2	[2]Ala-NH,2
	NH		8.26 d (5.2)		[2]Ala-2,3, [1]Aib-NH, [3]Ala-NH
[3]Ala	1	174.6 s		[4]Aib-NH, [3]Ala-2,3	
	2	51.1 d	4.01 m	[3]Ala-3,NH	[3]Ala-NH,3, [4]Aib-NH
	3	16.2 q	1.33 d (7.5)	[2]Ala-NH,2	[3]Ala-NH,2
	NH		7.68 d (6.0)		[4]Aib-NH, [3]Ala-2,3, [2]Ala-NH
[4]Aib	1	175.0 s		[5]Iva-NH, [4]Aib-3	
	2	56.4 s		[4]Aib-NH,3,4	
	3	26.0 [g] q	1.45 [f] s	[4]Aib-NH,4	[4]Aib-NH
	4	23.1 [e] q	1.35 [g] s	[4]Aib-NH,3	[4]Aib-NH
	NH		7.87 s		[4]Aib-2,3, [3]Ala-NH,2, [5]Iva-NH
[5]Iva	1	176.2 s		[6]Gln-NH,2, [5]Iva-3a,3b,NH	
	2	58.5 s		[5]Iva-NH,3a,3b,4,5	
	3a	25.8 t	2.21 m	[5]Iva-4	[5]Iva-NH,4,3b
	3b		1.62 m		[5]Iva-4,3a,NH
	4	7.4 q	0.73 m	[5]Iva-3a,3b	[5]Iva-NH,3a,3b
	5	22.7 q	1.35 s	[5]Iva-NH,3a,3b	[5]Iva-NH
	NH		7.47 s		[5]Iva-4,5,3a,3b, [6]Gln-NH, [4]Aib-NH
[6]Gln	1	174.1 s		[7]Aib-NH, [6]Gln-2	
	2	56.0 d	3.78 m	[6]Gln-NH,3,4a,4b	[7]Aib-NH,4, [6]Gln-NH,3,4a,4b
	3	26.2 t	1.97 m	[6]Gln-2,4a,4b,NH	[6]Gln-2,4b,NH, 7Aib-NH
	4a	31.6 t	2.14 m	[6]Gln-3,NH_2b	[6]Gln-NH_2a,2,4b,NH
	4b		2.23 m		[6]Gln-NH_2a,2,3,4a,NH
	5	173.6 s		[6]Gln-3,4a,4b,NH_2a,NH_2b	
	NH_2a		7.16 s		[6]Gln-NH_2b,4a,4b
	NH_2b		6.77 s		[6]Gln-NH_2a
	NH		7.73 m		[6]Gln-2,3,4a,4b, [5]Iva-NH
[7]Aib	1	176.0 s		[8]Aib-NH, [7]Aib-NH,3,4	
	2	56.4 [h] s		[7]Aib-NH,3,4	
	3	26.3 [g] q	1.43 [f] s	[7]Aib-NH,4	[7]Aib-NH
	4	22.7 [e] q	1.36 [g] s	[7]Aib-3	[7]Aib-NH, [6]Gln-2
	NH		7.90 s		[6]Gln-2,3, [7]Aib-3,4, [8]Aib-NH

Table S6. *Cont.*

^{8}Aib	1	176.4 s		^{9}Ala-NH, ^{8}Aib-3,4	
	2	56.0 s		^{8}Aib-NH,3,4	
	3	26.8 [g] q	1.45 [f] s	^{8}Aib-NH,4	^{8}Aib-NH
	4	22.9 [e] q	1.35 [g] s	^{8}Aib-3	^{8}Aib-NH
	NH		7.55 s		^{8}Aib-3,4, ^{7}Aib-NH, ^{9}Ala-NH
^{9}Ala	1	174.6 s		^{10}Ser-NH, ^{9}Ala-2,3	
	2	51.7 d	3.96 m	^{9}Ala-NH,3	^{9}Ala-NH
	3	16.5 q	1.40 d	^{9}Ala-NH,3	^{9}Ala-NH
	NH		7.72 s		^{9}Ala-2,3, ^{8}Aib-NH
^{10}Ser	1	170.6 s		^{11}Leu-NH, ^{10}Ser-2,3a,3b	
	2	58.1 d	4.10 m	^{10}Ser-NH	^{10}Ser-NH,3a,3b, ^{11}Leu-NH
	3a	61.1 t	3.73 m	^{10}Ser-NH,2	^{10}Ser-2
	3b		3.76 m		^{10}Ser-NH,2
	OH				
	NH		7.76 d (6.0)		^{10}Ser-2,3b, ^{11}Leu-NH
^{11}Leu	1	173.3 s		^{12}Aib-NH, ^{11}Leu-2	
	2	51.4 d	4.28 m	^{11}Leu-NH	^{12}Aib-NH, ^{11}Leu-NH,5,3a,3b,4, ^{13}Pro-5a, ^{14}Val-4
	3a	39.8 t	1.55 m	^{11}Leu-2,5,6	^{11}Leu-2,5,4
	3b		1.67 m		^{11}Leu-NH,2,5,6
	4	24.2 d	1.69 m	^{11}Leu-5,6	^{11}Leu-NH,2,3a
	5	20.9 q	0.78 d (6.8)	^{11}Leu-6,3b	^{11}Leu-2,3a,3b
	6	22.9 q	0.84 d	^{11}Leu-5,3a	^{11}Leu-3b,2
	NH		7.44 d (8.0)		^{10}Ser-2,NH, ^{11}Leu-2,3b,4
^{12}Aib	1	172.8 s		^{12}Aib-NH,3,4	
	2	56.2 [h] s		^{12}Aib-NH,3,4	
	3	26.4 q	1.39 s	^{12}Aib-NH	^{12}Aib-NH, ^{13}Pro-5b,2
	4	23.0 q	1.46 s	^{12}Aib	^{12}Aib-NH
	NH		7.91 s		^{13}Pro-5a,5b, ^{11}Leu-2, ^{12}Aib-3,4
^{13}Pro	1	173.8 s		^{14}Val-NH, ^{13}Pro-2,3a,5b	
	2	63.0 d	4.22 t (7.6)	^{13}Pro-3a,3b,4,5b	^{14}Val-NH, ^{13}Pro-4,3a,3b,5b,5a
	3a	28.7–28.8 t	1.65 m	^{13}Pro-2,5a,5b,4	^{13}Pro-2,3b, 5a
	3b		2.22 m		^{13}Pro-3a,2,5b,4
	4	25.9 t	1.86 m	^{13}Pro-2,3a,5b	^{13}Pro-2,3b,5a,5b
	5a	48.6 t	3.48 m	^{13}Pro-3a,3b,4	^{13}Pro-2,3a,4,5b, ^{11}Leu-2, ^{14}Val-NH, ^{12}Aib-NH
	5b		3.70 m		^{13}Pro-2,3b,4, 5a, ^{12}Aib-NH,3
^{14}Val	1	172.7 s		^{15}Aib-NH, ^{14}Val-2	
	2	61.1 d	3.68 m	^{14}Val-3,4,5,NH	^{14}Val-4,5,3,NH, ^{15}Aib-NH
	3	28.7–28.8 d	2.21 m	^{14}Val-2,4,5,NH	^{14}Val-2,4,5,NH
	4	19.2 q	0.94 d (6.4)	^{14}Val-5,2,3	^{14}Val-NH,2,3, ^{11}Leu-2
	5	19.1 q	0.89 d (7.2)	^{14}Val-4,2,3	^{14}Val-2,3
	NH		7.59 d (6.8)		^{14}Val-2,3,4, ^{13}Pro-2,5a, ^{15}Aib-NH

Table S6. *Cont.*

^{15}Aib	1	175.7 s		^{16}Ile-NH,2, ^{15}Aib-NH,3,4	
	2	56.1 [h] s		^{15}Aib-NH,3,4	
	3	26.5 [g] q	1.43 [f] s	^{15}Aib-4	^{15}Aib-NH
	4	23.3 [e] q	1.38 [g] s	^{15}Aib-NH,3	^{15}Aib-NH
	NH		7.43 s		^{15}Aib-3,4, ^{14}Val-NH,2, ^{16}Ile-NH
^{16}Ile	1	172.3 s		^{17}Glu-NH, ^{16}Ile-2	
	2	59.5 d	3.88 m	^{16}Ile-NH,6,4a,3	^{16}Ile-NH,6,4a,4b,3, ^{17}Glu-NH
	3	35.5 d	1.88 m	^{16}Ile-2,4a,5,6,NH	^{16}Ile-NH,2,6,4a
	4a	25.4 t	1.20 m	^{16}Ile-2,5,6	^{16}Ile-2,4b,NH,3
	4b		1.50 m		^{16}Ile-NH,2,4a,5
	5	11.4 q	0.81 m	^{16}Ile-4a,4b	^{16}Ile-4b
	6	15.7 q	0.85 m	^{16}Ile-4a,4b,2	^{16}Ile-NH,2,3
	NH		7.21 m		^{16}Ile-2,3,4a,4b,6, ^{17}Glu-NH, ^{15}Aib-NH
^{17}Glu	1	171.4 s		^{18}Gln-NH, ^{17}Glu-2	
	2	53.7 d	4.02 m	^{17}Glu-NH,4a,4b	^{17}Glu-NH,3,4a,4b
	3	26.3 t	1.95 m	^{17}Glu-2,4a,4b	^{17}Glu-NH,2
	4a	30.2 t	2.49 m	^{17}Glu-2,3	^{17}Glu-2,4b
	4b		2.40 m		^{17}Glu-NH,2,4a
	5	173.0 s		^{17}Glu-4a,4b,OCH_3	
	NH		7.68 d (6.0)		^{17}Glu-2,3,4b, ^{18}Gln-NH, ^{16}Ile-NH,2
	OCH_3	51.4 q	3.55 s		
^{18}Gln	1	170.9 s		^{19}Pheol-NH, ^{18}Gln-2	
	2	53.3 d	4.03 m	^{18}Gln-NH,3a,3b,4a,4b	^{19}PheOH-NH, ^{18}Gln-NH,3a,3b,4b
	3a	27.5 s	1.83 m	^{18}Gln-2,4a,4b,NH	^{18}Gln-NH,2,3b,4a
	3b		1.72 m		^{18}Gln-2,3a,4a
	4a	31.7 s	2.05 m	^{18}Gln-NH_2a,2,3a,3b	^{18}Gln-3a,3b,NH
	4b		1.98 m		^{18}Gln-NH, NH_2b,2
	5	173.6		^{18}Gln-NH2a,NH2b,3a,3b,4a,4b	
	NH_2a		6.63 s		^{18}Gln-NH_2b
	NH_2b		7.07 s		^{18}Gln-NH_2a,4b
	NH		7.46 d		^{19}Pheol-NH, ^{18}Gln-2,3b,4a,4b, ^{17}Glu-NH
^{19}Pheol	1a	62.8 t	3.30 m	^{19}Pheol-2,3a,3b	^{19}Pheol-NH,2,3a,3b
	1b		3.33 m		^{19}Pheol-NH,2,3a,3b
	2	52.6 d	3.86 m	^{19}Pheol-1a,1b,3a,3b,NH	^{19}Pheol-NH,1a,1b,3a,3b,5
	3a	36.7 t	2.62 dd (13.5, 8.5)	^{19}Pheol-1a,1b,2,5	^{19}Pheol-NH,1a,1b,2
	3b		2.84 dd (13.6, 5.0)		^{19}Pheol-1a,1b,2,5
	4	139.3 s		^{19}Pheol-2,3a,3b,6	
	5	129.4 d	7.22 m	^{19}Pheol-3a,3b,5,7	^{19}Pheol-2,3b
	6	128.1 d	7.20 m	^{19}Pheol-6	
	7	126.0 d	7.12 m	^{19}Pheol-5	
	OH				
	NH		7.19 m		^{18}Gln-NH,2, ^{19}Pheol-1a,1b,2,3a

[a] Carried out on an AVANCE-400, Bruker instrument; [b] Multiplicity and assignment are from HSQC experiment; [c] Values determined from HMBC experiment; $^{n}J_{CH}$ = 8 Hz, recycle time 1 s; [d] Selected NOE's from a ROESY experiment; [e,f,g,h] These signals may interchange.

Table S7. NMR data for TA1924a [a] (**7**) in DMSO-d_6.

**Position**		**δ_C, mult.** [b]	**δ_H, mult., *J* (Hz)**	**LR C-H correlations** [c]	**NOE correlations** [d]
Ac	1	171.1 s		Ac-2, [1]Aib-NH	
	2	23.1 q	1.93 s		[1]Aib-NH,3,4
[1]Aib	1	175.9 [e] s		[2]Ala-NH, [1]Aib-NH,3,4	
	2	55.7 [f] s		[1]Aib-NH,3,4	
	3	26.5 q	1.36 s	[1]Aib-NH	[1]Aib-NH
	4	24.1 q	1.32 s	[1]Aib-3	[1]Aib-NH
	NH		8.57 s		Ac-2, [1]Aib-3,4
[2]Ala	1	174.6 s	-	[3]Ala-NH, [2]Ala-2,3	
	2	50.9 d	4.01 m	[2]Ala-NH,3	[2]Ala-NH,3
	3	16.8 q	1.31 d	[2]Ala-NH,2	[2]Ala-NH,2
	NH		8.27 d (5.4)		[3]Ala-NH, [2]Ala-2,3
[3]Ala	1	174.7 s	-	[4]Aib-NH, [3]Ala-2,3	
	2	51.2 d	4.01 m	[3]Ala-NH,3	[3]Ala-NH,3, [4]Aib-NH
	3	16.1 q	1.33 d	[2]Ala-NH,2	3Ala-NH,2
	NH		7.71 m		[4]Aib-NH, [2]Ala-NH, [3]Ala-2,3
[4]Aib	1	175.0 s		[5]Aib-NH,[4]Aib-NH,3,4	
	2	56.0 [f] s		[4]Aib-NH,3,4	
	3	26.1 q	1.45 s	[4]Aib-NH,4	[4]Aib-NH
	4	22.5 [g] q	1.38 [i] s	[4]Aib-3	[4]Aib-NH
	NH		7.83 s		[4]Aib-4,3, [3]Ala-NH,2, [5]Aib-NH
[5]Aib	1	176.0 [e] s		[6]Gln-NH, [5]Aib-NH,3,4	
	2	55.8 [f] s		[5]Aib-NH,3a,3b,4,5	
	3	26.6 q	1.44 s	[5]Aib-NH,4	[5]Aib-NH
	4	22.7 [g] q	1.36 [i] s	[5]Aib-3	[5]Aib-NH,
	NH		7.55 s		[5]Aib-3,4, [4]Aib-NH
[6]Gln	1	173.8 s		[7]Aib-NH, [6]Gln-2,3	
	2	56.0 d	3.77 m	[6]Gln-NH,3,4a,4b	[7]Aib-NH, [6]Gln-NH,3,4a,4b
	3	26.2 t	1.97 m	[6]Gln-2,4a,4b,NH	[6]Gln-2, NH, [7]Aib-NH
	4a	31.5 [g] t	2.15 m	[6]Gln-2,3,NH_2b	[6]Gln-2,4b,NH_2a
	4b		2.23 m		[6]Gln-2,4a,NH_2a
	5	173.7 s		[6]Gln-3,4a,4b,NH_2a,NH_2b	
	NH_2a		7.16 s		[6]Gln-4a,4b,NH_2b
	NH_2b		6.76 s		[6]Gln-NH_2 a
	NH		7.77 m		[6]Gln-2,3
[7]Aib	1	176.0 [e] s		[8]Aib-NH, [7]Aib-NH,3,4	
	2	55.9 [f] s		[7]Aib-NH,3,4	
	3	26.2 q	1.45 [j] s	[7]Aib-NH,4	[7]Aib-NH
	4	22.8 [g] q	1.35 [i] s	[7]Aib-3	[7]Aib-NH
	NH		7.86 s		[6]Gln-2,3, [7]Aib-3,4, [8]Aib-NH
[8]Aib	1	175.6 s		[9]Aib-NH, [8]Aib-3,4	
	2	56.0 [f] s		[8]Aib-NH,3,4	
	3	26.6 [h] q	1.43 [j] s	[8]Aib-NH,4	[8]Aib-NH
	4	22.8 [g] q	1.35 [i] s	[8]Aib-3	[8]Aib-NH
	NH		7.59 s		[8]Aib-3,4, [7]Aib-NH

Table S7. *Cont.*

^{9}Aib	1	176.4 s		^{10}Ser-NH, ^{9}Aib-3,4,NH	
	2	56.1 [f] s		^{9}Aib-NH,3,4	
	3	26.5 [h] q	1.48 s	^{9}Aib-4	^{9}Aib-NH
	4	22.9 [g] q	1.43 s	^{9}Aib-NH,3	^{9}Aib-NH
	NH		7.79 s		^{9}Aib-3,4
^{10}Ser	1	170.6 s		^{11}Leu-NH, ^{10}Ser-2,3a	-
	2	58.9 d	4.03 m	^{10}Ser-NH	^{10}Ser-NH,3a,3b, ^{11}Leu-NH
	3a	61.2 t	3.75 m	^{10}Ser-NH,2	^{10}Ser-NH,2
	3b		3.79 m		^{10}Ser-NH,2
	NH		7.74 m		^{10}Ser-2,3a,3b
^{11}Leu	1	173.7 s	-	^{12}Aib-NH, ^{11}Leu-2	
	2	51.6 d	4.27 m	^{11}Leu-NH,4	^{11}Leu-NH,5,3a,3b, ^{13}Pro-5a
	3a	39.2 t	1.56 m	^{11}Leu-2.5,6	^{11}Leu-2,5,6,3b
	3b		1.70 m		^{11}Leu-NH,2,3a,6
	4	23.9 d	1.73 m	^{11}Leu-5,6	^{11}Leu-NH,5
	5	20.5 q	0.76 d	^{11}Leu-6,3b	^{11}Leu-2,3a,4
	6	23.0 q	0.82 d	^{11}Leu-5,3b	^{11}Leu-3a,3b
	NH		7.59 d		^{10}Ser-2, ^{11}Leu-2,3b,4
^{12}Aib	1	173.0 s		^{12}Aib-NH,3,4	
	2	56.3 [f] s		^{12}Aib-NH,3,4	
	3	25.8 q	1.37 s	^{12}Aib-3	^{12}Aib-NH
	4	23.1 q	1.49 s	^{12}Aib-NH,4	^{12}Aib-NH, ^{13}Pro-5b
	NH		7.92 s		^{12}Aib-3,4
^{13}Pro	1	173.3 s	-	^{14}Leu-NH, ^{13}Pro-2,3a	-
	2	63.0 d	4.21 t (8.0)	^{13}Pro-3a,4,5b	^{14}Leu-5, ^{13}Pro-4,3b
	3a	28.7 t	1.60 m	^{13}Pro-2,5b,4	^{13}Pro-3b, 5a
	3b		2.24 m		^{13}Pro-3a,2, 4
	4	26.1 t	1.86 m	^{13}Pro-2,5b,5a,3a	^{13}Pro-2,3b,5a,5b
	5a	48.7 t	3.45 m	^{13}Pro-3b	^{13}Pro-2,3a,4, ^{11}Leu-2, ^{14}Leu-NH
	5b		3.70 m		^{13}Pro-4, ^{12}Aib-3
^{14}Leu	1	173.8 s	-	^{15}Aib-NH, ^{14}Leu-2	-
	2	53.6 d	3.90 m	^{14}Leu-NH	^{14}Leu-4,3a,3b,5,6,NH, ^{15}Aib-NH
	3a	38.7 t	1.52 m	^{14}Leu-4,5,6	^{14}Leu-2,3b,5,6
	3b		1.78 m		^{14}Leu-2,3a,5,6,NH,4
	4	24.8 d	1.68 m	^{14}Leu-5,6	^{14}Leu-2,5,6,NH,3b
	5	23.0 q	0.92 d (6.0)	^{14}Leu-6,4,3a	^{14}Leu-2,4,3a,3b, ^{13}Pro-2
	6	20.9 q	0.82 d	^{14}Leu-5,4	^{14}Leu-2,3a,3b,4
	NH		7.72 m		^{14}Leu-2, 3b,4, ^{13}Pro-5a
^{15}Aib	1	175.3 s		^{16}Ile-NH, ^{15}Aib-NH,3,4	
	2	56.2 [f] s		^{15}Aib-NH,3,4	
	3	26.1 [h] q	1.42 [j] s	^{15}Aib-NH,4	^{15}Aib-NH
	4	23.4 [g] q	1.36 [i] s	^{15}Aib-3	^{15}Aib-NH
	NH		7.63 s		^{15}Aib-3,4, ^{16}Ile-NH,2, ^{14}Leu-2

Table S7. *Cont.*

^{16}Ile	1	171.9 s	-	^{17}Glu-NH, ^{16}Ile-2	-
	2	58.9 d	3.93 m	^{16}Ile-NH, ^{16}Ile-6,4b,3	^{15}Aib-NH, ^{16}Ile-NH,6,4b,3, ^{17}Glu-NH
	3	35.6 d	1.88 m	^{16}Ile-2,4a,4b,5,6,NH	^{16}Ile-NH,2,5,6
	4a	25.0 t	1.20 m	^{16}Ile-2,5,6	^{16}Ile-NH,4b
	4b		1.47 m		^{16}Ile-NH,2,4a,5
	5	11.5 q	0.82 t	^{16}Ile-4a,4b	^{16}Ile-4b,3
	6	15.7 q	0.86 d	^{16}Ile-4a,4b,2	^{16}Ile-2,3
	NH		6.94 d		^{16}Ile-2,3,4a,4b, ^{15}Aib-NH
^{17}Glu	1	171.1 s		^{18}Gln-NH, ^{17}Glu-2,3a,3b	
	2	53.1 d	4.06 m	^{17}Glu-NH,4a,4b,3b	^{18}Gln-NH, ^{17}Glu-NH,3a,3b,4a
	3a	26.5 t	1.87 m	^{17}Glu-2,4a,4b	^{17}Glu-NH,2,4b
	3b		1.97 m		^{17}Glu-NH,2.4a
	4a	30.1 t	2.37 m	^{17}Glu-2	^{17}Glu-NH,2,3b
	4b		2.44 m		^{17}Glu-NH,3a
	5	172.9 s		^{17}Glu-4a,4b,OCH_3	
	NH		7.68 m		^{17}Glu-2,3a, ^{16}Ile-2
	OCH_3	51.4 q	3.54 s		
^{18}Gln	1	170.8 s		^{19}Pheol-NH, ^{18}Gln-2,3a,3b	
	2	53.0 d	4.06 m	^{18}Gln-NH,3a,3b,4a,4b	^{19}Pheol-NH, ^{18}Gln-NH,3a,3b,4b
	3a	27.7 t	1.83 m	^{18}Gln-2,4a,4b,NH	^{18}Gln-NH,2,3b,4a
	3b		1.70 m		^{18}Gln-NH,2,3a,4a
	4a	31.6 ^{g} t	2.05 m	^{18}Gln-NH_2a,2,3a,3b	^{18}Gln-3a,3b
	4b		1.97 m		^{18}Gln-NH,NH_2b,2
	5	173.6 s		^{18}Gln-NH_2a,NH_2b,3a,3b, 4a,4b	
	NH_2a		6.65 s		^{18}Gln-NH_2b
	NH_2b		7.10 s		^{18}Gln-NH_2a,4b
	NH		7.48 d (8.0)		^{19}Pheol-NH, ^{18}Gln-2,3a,3b,4b
^{19}Pheol	1a	62.7 t	3.28 m	^{19}Pheol-2,3a,3b	^{19}Pheol-NH,2,3a,3b,5
	1b		3.32 m		^{19}Pheol-NH,2,3a,3b
	2	52.5 d	3.87 m	^{19}Pheol-1a,1b,3a,3b,NH	^{19}Pheol-NH,1a,1b,3a,3b,5
	3a	36.6 t	2.61 dd (13.5, 8.3)	^{19}Pheol-1a,1b,2,5	^{19}Pheol-NH,1a,1b,2,5
	3b		2.84 dd (13.6, 5.3)		^{19}Pheol-1a,1b,2,5
	4	139.2 s		^{19}Pheol-2,3a,3b,5,7	
	5	129.4 d	7.21 m	^{19}Pheol-3a,3b,5,7	^{19}Pheol-1a,2,3a,3b,7
	6	128.1 d	7.19 m	^{19}Pheol-5,7	
	7	126.0 d	7.13 m	^{19}Pheol-5,6	^{19}Pheol-5
	OH				
	NH		7.30 d (8)		^{18}Gln-NH,2, ^{19}Pheol-1a,1b,2,3a

[a] Carried out on an AVANCE-500, Bruker instrument; [b] Multiplicity and assignment are from HSQC experiment; [c] Values determined from HMBC experiment; $^{n}J_{CH}$ = 8 Hz, recycle time 1 s; [d] Selected NOE's from a ROESY experiment; [e,f,g,h,i,j] These signals may interchange.

Table S8. NMR data for TA1909a [a] (**8**) in DMSO-d_6.

Position		δ_C, mult. [b]	δ_H, mult., *J* (Hz)	LR C-H correlations [c]	NOE correlations [d]
Ac	1	171.1 s		Ac-2, 1Aib-NH	
	2	23.1 q	1.93 s		1Aib-NH
1Aib	1	175.8 s		2Ala-NH, 1Aib-NH,3,4	
	2	55.8 [h] s		1Aib-NH,3,4	
	3	26.3 q	1.36 s	1Aib-NH	1Aib-NH
	4	23.7 q	1.33 s	1Aib-3	1Aib-NH
	NH		8.56 s		Ac-2, 1Aib-3,4
2Ala	1	174.6 s		3Ala-NH, 2Ala-2,3	
	2	50.9 d	4.01 m	2Ala-NH,3	2Ala-NH,3
	3	16.9 q	1.31 d	2Ala-NH,2	2Ala-NH,2
	NH		8.25 d (6.0)		2Ala-2,3, 3Ala-NH
3Ala	1	174.6 s		4Aib-NH, 3Ala-2,3	
	2	51.1 d	4.01 m	3Ala-3	3Ala-NH,3, 4Aib-NH
	3	16.2 q	1.33 d (7.5)	2Ala-NH,2	3Ala-NH,2
	NH		7.68 d (6.0)		4Aib-NH, 3Ala-2,3, 2Ala-NH
4Aib	1	175.0 s		5Iva-NH, 4Aib-3,NH	
	2	56.2 [h] s		4Aib-NH,3,4	
	3	26.3 q	1.46 s	4Aib-NH,4	4Aib-NH
	4	23.3 [e] q	1.35 [f] s	4Aib-3	4Aib-NH
	NH		7.87 s		4Aib-2,3, 3Ala-NH,2, 5Iva-NH
5Iva	1	176.2 s		6Gln-NH, 5Iva-NH	
	2	58.5 s		5Iva-NH,3a,3b,4,5	
	3a	25.9 t	2.21 m	5Iva-4	5Iva-NH,4,3b
	3b		1.62 m		5Iva-3a
	4	7.4 q	0.73 t (7.4)		5Iva-NH,3a
	5	22.6 q	1.35 s	5Iva-3a	5Iva-NH
	NH		7.47 s		5Iva-4,5,3a, 6Gln-NH, 4Aib-NH
6Gln	1	174.1 s		7Aib-NH, 6Gln-2	
	2	56.0 [h] d	3.78 m	6Gln-4a,4b	7Aib-NH,4,6Gln-NH,3,4a,4b
	3	26.2 t	1.97 m	6Gln-2,4a,4b	6Gln-2,NH, 7Aib-NH
	4a	31.7 t	2.14 m	6Gln-NH_2b	6Gln-NH_2 a, 2,4b,NH
	4b		2.23 m		6Gln-NH_2 a 2,4a,NH
	5	173.6 [e] s		6Gln-3,4a,4b,NH_2a	
	NH_2a		7.16 s		6Gln-NH_2b,4a,4b
	NH_2b		6.76 s		6Gln-NH_2a
	NH		7.73 m		6Gln-2,3,4a,4b, 5Iva-NH
7Aib	1	176.0 s		8Aib-NH, 7Aib-3,4	
	2	56.2 [h] s		7Aib-NH,3,4	
	3	26.2 [g] q	1.43 [j] s	7Aib-NH,4	7Aib-NH
	4	22.9 [e] q	1.36 [f] s	7Aib-3	7Aib-NH, 6Gln-2
	NH		7.90 s		6Gln-2,3, 7Aib-3,4, 8Aib-NH
8Aib	1	176.4 s		9Ala-NH, 8Aib-3,4,NH	
	2	56.1 [h] s		8Aib-NH,3,4	
	3	26.4 [g] q	1.43 [j] s	8Aib-4	8Aib-NH
	4	23.2 [e] q	1.39 [f] s	8Aib-3	8Aib-NH
	NH		7.55 s		8Aib-3,4, 7Aib-NH, 9Ala-NH

Table S8. *Cont.*

^{9}Ala	1	174.6 s		^{10}Ser-NH, ^{9}Ala-2,3	
	2	51.7 d	3.96 m	^{9}Ala-3	9Ala-NH
	3	16.5 q	1.40 d	^{9}Ala-NH,3	^{9}Ala-NH
	NH		7.72 s		^{9}Ala-2,3, Aib-NH
^{10}Ser	1	170.6 s		^{10}Ser-2,3a,3b, ^{11}Leu-NH	
	2	58.1 d	4.11 m		^{10}Ser-NH,OH, ^{11}Leu-NH
	3a	61.0 [i] t	3.72 m	^{10}Ser-2	^{10}Ser-NH,OH
	3b		3.76		^{10}Ser-NH,OH
	OH		4.87 t (6.2)		^{10}Ser-NH,2,3a,3b
	NH		7.76 d		^{10}Ser-2,3b,OH, ^{11}Leu-NH
^{11}Leu	1	173.3 s		^{12}Aib-NH, ^{11}Leu-2	
	2	51.4 d	4.28 m		^{12}Aib-NH, ^{11}Leu-NH,5,3a,3b,4, ^{13}Pro-5a
	3a	39.8 t	1.55 m	^{11}Leu-5,6	^{11}Leu-2,6,4
	3b		1.67 m		^{11}Leu-NH,2,6
	4	24.0 d	1.69 m	^{11}Leu-5,6	^{11}Leu-NH,2,3a
	5	21.0 q	0.78 d (6.8)	^{11}Leu-6	^{11}Leu-2
	6	22.9 q	0.84 m	^{11}Leu-5,3a	^{11}Leu-3a,3b,2
	NH		7.44 m		^{10}Ser-2,NH, ^{11}Leu-2,3b,4, ^{12}Aib-NH
^{12}Aib	1	172.7 s		^{12}Aib-NH,3,4	
	2	56.1 [h] s		^{12}Aib-3,4	
	3	25.5 q	1.40 s		^{12}Aib-NH
	4	22.9 q	1.49 s		^{12}Aib-NH, ^{13}Pro-5b,2
	NH		7.93 s		^{13}Pro-5b, ^{11}Leu-2,NH, ^{12}Aib-3,4
^{13}Pro	1	173.8 s		^{14}Val-NH, ^{13}Pro-2,3a	
	2	63.0 d	4.24 t (7.6)	^{13}Pro-4	^{14}Val-NH, ^{13}Pro-4, 3b
	3a	29.1 t	1.65 m	^{13}Pro-2,4	^{13}Pro-3b
	3b		2.22 m		^{13}Pro-3a,2,5b
	4	26.0 t	1.86 m	^{13}Pro-5b	^{13}Pro-2,5a,5b
	5a	48.6 t	3.48 m		^{13}Pro-4,5b, ^{11}Leu-2, ^{14}Val-NH
	5b		3.70 m		^{13}Pro-3b,4,5a, ^{12}Aib-NH,3
^{14}Val	1	172.7 s		^{15}Aib-NH, ^{14}Val-2	
	2	61.1 [i] d	3.77 m	^{14}Val-3,4,5	^{14}Val-4,5,3,NH, ^{15}Aib-NH
	3	28.8 d	2.21 m	^{14}Val-2,4,5	^{14}Val-2,4,5,NH
	4	19.2 q	0.94 d (6.4)	^{14}Val-5,2,3	^{14}Val-NH,2,3
	5	19.2 q	0.88 d	^{14}Val-4,2,3	^{14}Val-2,3
	NH		7.60 d (7.2)		^{14}Val-2,3,4, ^{13}Pro-2,5a, ^{15}Aib-NH
^{15}Aib	1	175.7 s		^{16}Ile-NH,2, ^{15}Aib-NH,3,4	
	2	56.1 [h] s		^{15}Aib-3,4	
	3	26.3 [g] q	1.45 [j] s	^{15}Aib-4	^{15}Aib-NH
	4	23.2 [e] q	1.39 [f] s	^{15}Aib-3	^{15}Aib-NH
	NH		7.43 s		^{15}Aib-3,4, ^{14}Val-NH,2

Table S8. *Cont.*

^{16}Ile	1	172.4 s		^{17}Gln-NH, ^{16}Ile-2	
	2	59.6 d	3.87 m	^{16}Ile-6	^{16}Ile-NH,6,4b,3, ^{17}Gln-NH
	3	35.5 d	1.88 m	^{16}Ile-2,4a,5,6	^{16}Ile-NH,2,6
	4a	25.4 t	1.20 m	^{16}Ile-2,5,6	^{16}Ile-4b,NH
	4b		1.50 m		^{16}Ile-NH,2,4a,5
	5	11.4 q	0.81 t	^{16}Ile-4a	^{16}Ile-4b
	6	15.7 q	0.86 d	^{16}Ile-2	^{16}Ile-NH,2,3
	NH		7.22 m		^{16}Ile-2,3,4a,4b,6
^{17}Gln	1	171.8 s		^{18}Gln-NH, ^{17}Gln-2	
	2	54.3 d	3.97 m	^{17}Gln-4a,4b	^{17}Gln-NH,3,4a,4b
	3	26.8 t	1.90 m	^{17}Gln-2,4a,4b	^{17}Gln-NH,2
	4a	31.7 t	2.13 m	^{17}Gln-NH_2a	^{17}Gln-NH,2,NH_2b
	4b		2.23 m		^{17}Gln-NH,2,NH_2b
	5	173.7 [e] s		^{17}Gln-4a,4b,NH_2b	
	NH_2a		6.72 s		^{17}Gln-NH_2b
	NH_2b		7.13 s		^{17}Gln-NH_2a,4a,4b
	NH		7.69 d (5.6)		^{17}Gln-2,3,4a,4b, ^{16}Ile-2, ^{18}Gln-NH
^{18}Gln	1	171.0 s		^{19}Pheol-NH,^{18}Gln-2	
	2	53.4 d	4.03 m	^{18}Gln-NH,4a,4b	^{19}Pheol-NH, ^{18}Gln-NH,3a,3b,4b
	3a	27.4 t	1.85 m	^{18}Gln-4a,4b	^{18}Gln-NH,2,3b,4a
	3b		1.72 m		^{18}Gln-2,3a,4a
	4a	31.5 s	2.05 m	1^{8}Gln-NH_2a	^{18}Gln-3a,3b,NH
	4b		1.98 m		^{18}Gln-NH,NH_2b,2
	5	173.8 [e] s		^{18}Gln-NH_2b,3b,4a,4b	
	NH_2a		6.63 s		^{18}Gln-NH_2b
	NH_2b		7.07 s		^{18}Gln-NH_2a,4b
	NH		7.46 d		^{18}Gln-2,3b,4a,4b, ^{17}Gln-NH
^{19}Pheol	1a	62.9 t	3.30 m	^{19}Pheol-3a,3b	^{19}Pheol-OH
	1b		3.33 m		^{19}Pheol-OH
	2	52.6 d	3.86 m	^{19}Pheol-1a,1b,OH	^{19}Pheol-NH,3a,3b,5,OH
	3a	36.7 t	2.63 dd (13.5, 8.2)	^{19}Pheol-1a,1b,5	^{19}Pheol-NH,2
	3b		2.84 dd (13.5, 5.2)		^{19}Pheol-2,5
	4	139.3 s		^{19}Pheol-2,3a,3b,6	
	5	129.4 d	7.22 m	^{19}Pheol-3a,3b,5,7	^{19}Pheol-2,3b
	6	128.1 d	7.20 m	^{19}Pheol-6	
	7	126.0 d	7.12 m	^{19}Pheol-5	
	OH		4.69 t (6.2)		^{19}Pheol-1a,1b,2,NH
	NH		7.18 m		^{18}Gln-2, ^{19}Pheol-2,3a,OH

[a] Carried out on an AVANCE-400, Bruker instrument; [b] Multiplicity and assignment are from HSQC experiment; [c] Values determined from HMBC experiment; $^{n}J_{CH}$=8 Hz, recycle time 1 s; [d] Selected NOE's from a ROESY experiment; [e,f,g,h,i,j] These signals may interchange.

Table S9. NMR data for TA.VIb [a] (**9**) in DMSO-d_6.

Position		δ_C, mult. [b]	δ_H, mult., *J* (Hz)	LR C-H correlations [c]	NOE correlations [d]
Ac	1	171.2 s		Ac-2, ^{1}Aib-NH	
	2	23.0 q	1.93 s		^{1}Aib-NH
^{1}Aib	1	175.9 s		^{2}Ala-NH, ^{1}Aib-NH,3,4	
	2	55.8 s		^{1}Aib-NH,3,4	
	3	26.1 q	1.36 s	^{1}Aib-NH	^{1}Aib-NH
	4	24.2 q	1.33 s	^{1}Aib-3	^{1}Aib-NH
	NH		8.55 s		Ac-2, ^{1}Aib-3,4
^{2}Ala	1	174.6 s		^{3}Ala-NH, ^{2}Ala-2,3	
	2	51.0 d	3.99 m	^{2}Ala-NH,3	^{2}Ala-NH,3
	3	16.9 q	1.31 d	^{2}Ala-NH,2	^{2}Ala-NH,2
	NH		8.25 d (5.1)		^{2}Ala-2,3, ^{3}Ala-NH
^{3}Ala	1	174.7 s		^{4}Aib-NH, ^{3}Ala-2,3	
	2	51.2 d	4.01 m	^{3}Ala-NH,3	^{3}Ala-NH, 3,^{4}Aib-NH
	3	16.2 q	1.33 d (7.5)	^{2}Ala-NH,2	^{3}Ala-NH,2
	NH		7.70 d (6.0)		^{4}Aib-NH, ^{3}Ala-2,3, ^{2}Ala-NH
^{4}Aib	1	175.1 s		^{5}Aib-NH, ^{4}Aib-3	
	2	56.0 [j] s		^{4}Aib-NH,3,4	
	3	26.2 [h] q	1.43 [f] s	^{4}Aib-NH,4	^{4}Aib-NH
	4	23.4 [i] q	1.36 [k] s	^{4}Aib-NH,3	^{4}Aib-NH
	NH		7.85 s		^{4}Aib-2,3, ^{3}Ala-NH,2
^{5}Aib	1	176.0 [e] s		^{6}Gln-NH, ^{5}Aib-3,4,NH	
	2	55.9 [j] s		^{5}Aib-NH,3,4	
	3	26.6 [h] q	1.44 s	^{5}Aib-NH,4	^{5}Aib-NH
	4	22.7 q	1.39 s	^{5}Aib-3	^{5}Aib-NH
	NH		7.54 s		^{5}Aib-3,4
^{6}Gln	1	173.9 s		^{7}Aib-NH, ^{6}Gln-2	
	2	56.0 d	3.78 m	^{6}Gln-NH,3,4a,4b	^{7}Aib-NH, ^{6}Gln-NH,3,4a,4b
	3	26.2 t	1.98 m	^{6}Gln-2,4a,4b,NH	^{6}Gln-2,4a,4b,NH, ^{7}Aib-NH
	4a	31.6 [g] t	2.14 m	^{6}Gln-2,3,NH_2b	^{6}Gln-NH_2a,2,3,4b,NH
	4b		2.23 m		^{6}Gln-NH_2a,2,3,4a
	5	173.7 s		^{6}Gln-3,4a,4b,NH_2a,NH_2b	
	NH_2a		7.14 s		^{6}Gln-4a,NH_2b,4a,4b
	NH_2b		6.74 s		^{6}Gln-NH_2a
	NH		7.75 m		^{6}Gln-2,3,4a
^{7}Aib	1	176.1 [e] s		^{8}Aib-NH, ^{7}Aib-NH,3,4	
	2	56.1 [j] s		^{7}Aib-NH,3,4	
	3	26.1 [h] q	1.42 [f] s	^{7}Aib-NH,4	^{7}Aib-NH
	4	23.0 [i] q	1.38 [k] s	^{7}Aib-NH	^{7}Aib-NH
	NH		7.85 s		^{6}Gln-2,3, ^{7}Aib-3,4

Table S9. *Cont.*

^{8}Aib	1	175.7 s		^{9}Aib-NH, ^{8}Aib-3,4,NH	
	2	56.1 [j] s		^{8}Aib-NH,3,4	
	3	26.6 [h] q	1.43 [f] s	^{8}Aib-NH,4	^{8}Aib-NH
	4	23.2 [i] q	1.35 [k] s	^{8}Aib-3	^{8}Aib-NH
	NH		7.58 s		^{8}Aib-3,4
^{9}Aib	1	176.5 s		^{10}Ser-NH, ^{9}Aib-3,4	
	2	56.2 [j] s		^{9}Aib-NH,3,4	
	3	26.7 [h] q	1.47 s	^{9}Aib-NH,4	^{9}Aib-NH
	4	23.3 [i] q	1.44 s	^{9}Aib-3	^{9}Aib-NH
	NH		7.78 s		^{9}Aib-3,4
^{10}Ser	1	170.7 s		^{11}Leu-NH, ^{10}Ser-2,3a	
	2	59.0 d	4.03 m	^{10}Ser-NH	^{10}Ser-NH,3a,3b
	3a	61.2 t	3.74 m	^{10}Ser-NH,2	^{10}Ser-NH,2
	3b		3.75 m		^{10}Ser-NH,2
	NH		7.76 m		^{10}Ser-2,3a,3b
^{11}Leu	1	173.6 s		^{12}Aib-NH, ^{11}Leu-2	
	2	51.4 d	4.28 m	^{11}Leu-3b	^{11}Leu-NH,5,3a,3b,4, ^{14}Val-4
	3a	39.8 t	1.54 m	^{11}Leu-2,5,6	^{11}Leu-2,5
	3b		1.70 m		^{11}Leu-NH,2,5,6
	4	24.2 d	1.70 m	^{11}Leu-5,6	^{11}Leu-NH,2,5,6
	5	20.8 q	0.77 d	^{11}Leu-6,4	^{11}Leu-2,3a,3b
	6	22.9 q	0.83 d	^{11}Leu-5,3a	^{11}Leu-3b
	NH		7.62 m		^{11}Leu-2,3b,4, ^{12}Aib-NH
^{12}Aib	1	172.8 s		^{12}Aib-NH,3,4	
	2	56.3 s		^{12}Aib-NH,3,4	
	3	25.5 q	1.40 s	^{12}Aib-NH,4	^{12}Aib-NH,^{13}Pro-5b
	4	22.9 q	1.49 s	^{12}Aib-3	^{12}Aib-NH
	NH		7.95 s		^{12}Aib-3,4, ^{11}Leu-NH
^{13}Pro	1	173.8 s		^{13}Pro-2,3a, ^{14}Val-NH	
	2	63.1 d	4.25 t (7.8)	^{13}Pro-3a,4	^{13}Pro-4,3a,3b,
	3a	28.9 t	1.67 m	^{13}Pro-2,4,5b	^{13}Pro-2,3b,4
	3b		2.22 m		^{13}Pro-3a,2,4,5b
	4	25.8 t	1.87 m	^{13}Pro-2,3a,5a,5b	^{13}Pro-2,3a,3b,5a,5b
	5a	48.6 t	3.53 m	^{13}Pro-3a,3b,4	^{13}Pro-4,5b
	5b		3.69 m		^{13}Pro-3b,4,5a, ^{12}Aib-3
^{14}Val	1	172.7 s		^{15}Aib-NH, ^{14}Val-2	
	2	61.0 d	3.80 m	^{14}Val-3,4,5	^{14}Val-4,5,3,NH, ^{15}Aib-NH
	3	28.9 d	2.21 m	^{14}Val-2,4,5	^{14}Val-2,4,5,NH
	4	19.2 q	0.95 d (6.4)	^{14}Val-5,2,3	^{14}Val-NH,2,3, ^{11}Leu-2
	5	19.2 q	0.89 d (6.8)	^{14}Val-4,2,3	^{14}Val-2,3
	NH		7.60 d		^{14}Val-2,3,4

Table S9. *Cont.*

^{15}Aib	1	175.7 s		^{16}Ile-NH,2, ^{15}Aib-NH,3,4	
	2	55.8 s		^{15}Aib-NH,3,4	
	3	26.3 [h] q	1.43 [f] s	^{15}Aib-NH,4	^{15}Aib-NH
	4	23.0 [i] q	1.36 [k] s	^{15}Aib-3	^{15}Aib-NH
	NH		7.47 s		^{15}Aib-3,4, ^{14}Val-2
^{16}Ile	1	172.4 s		^{17}Gln-NH, ^{16}Ile-2	
	2	59.6 d	3.87 m	^{16}Ile-NH,6,4a,4b	^{16}Ile-NH,5,6,4a,4b,3, ^{17}Gln-NH
	3	35.6 d	1.88 m	^{16}Ile-2,4a,5,6,NH	^{16}Ile-NH,2,5,6
	4a	25.4 t	1.20 m	^{16}Ile-2,3,5,6	^{16}Ile-2,4b,NH
	4b		1.50 m		^{16}Ile-4a,5,2
	5	11.4 q	0.81 t (8.0)	^{16}Ile-4a,4b	^{16}Ile-4b,2,3
	6	15.8 q	0.85 d	^{16}Ile-4a,4b, 2	^{16}Ile-3,NH,2
	NH		7.22 m		^{16}Ile-2,3,4a, 6
^{17}Gln	1	171.8 s		^{18}Gln-NH, ^{17}Gln-2	
	2	54.3 d	3.98 m	^{17}Gln-NH,4a,4b	^{17}Gln-NH,3,4a,4b,NH_2b
	3	26.9 t	1.93 m	^{17}Gln-2,4a,4b,NH	^{17}Gln-NH,2
	4a	31.8 [g] t	2.13 m	^{17}Gln-NH2a,2,3	^{17}Gln-NH,2,NH_2b
	4b		2.23 m		^{17}Gln-2,NH_2b
	5	173.7 s		^{17}Gln-4a,4b,NH_2a,NH_2b	
	NH_2a		6.71 s		^{17}Gln-NH_2b
	NH_2b		7.14 s		^{17}Gln-NH_2a,4a,4b,2
	NH		7.70 d (6.0)		^{17}Gln-2,3,4a, ^{16}Ile-2
^{18}Gln	1	171.0 s		^{19}Pheol-NH, ^{18}Gln-2	
	2	53.4 d	4.02 m	^{18}Gln-NH,3a,4a,4b	^{19}Pheol-NH, ^{18}Gln-NH,3a, 3b,4b
	3a	27.5	1.83 m	^{18}Gln-2,4a,4b,NH	^{18}Gln-2,3b,4a
	3b		1.70 m		^{18}Gln-2,3a,4a,NH
	4a	31.7 [g] t	2.05 m	^{18}Gln-NH_2a,2,3a,3b	^{18}Gln-3a,3b
	4b		1.98 m		^{18}Gln-NH,NH_2b,2
	5	173.7 s		^{18}Gln-NH_2a,NH_2b,4a,4b	
	NH_2a		6.61 s		^{18}Gln-NH_2b
	NH_2b		7.06 s		^{18}Gln-NH_2a,4b
	NH		7.46 d		^{18}Gln-2,3b,4b
^{19}Pheol	1a	62.9 t	3.31 m	^{19}Pheol-2,3a,3b	^{19}Pheol-NH
	1b		3.34 m		^{19}Pheol-NH
	2	52.7 d	3.87 m	^{19}Pheol-1a,1b,3a,3b,NH	^{19}Pheol-NH,3a,3b,5
	3a	36.8 t	2.63 dd (13.4, 8.4)	^{19}Pheol-1a,1b,5	^{19}Pheol-NH,2
	3b		2.83 dd (13.5, 4.9)		^{19}Pheol-2
	4	139.4 s		^{19}Pheol-2,3a,3b,6	
	5	129.5 d	7.22 m	^{19}Pheol-3a,3b,5,7	^{19}Pheol-2,7
	6	128.2 d	7.20 m	^{19}Pheol-6	
	7	126.0 d	7.13 m	^{19}Pheol-5	^{19}Pheol-5
	OH				
	NH		7.18 m		^{18}Gln-2, ^{19}Pheol-1a,1b,2,3a

[a] Carried out on an AVANCE-400, Bruker instrument; [b] Multiplicity and assignment are from HSQC experiment; [c] Values determined from HMBC experiment; $^{n}J_{CH}$ = 8 Hz, recycle time 1 s; [d] Selected NOE's from a ROESY experiment; [e,f,g,h,i,j,k] These signals may interchange.

Table S10. NMR data for TA.VIa [a] (**10**) in DMSO-d_6.

Position		δ$_C$, mult. [b]	δ$_H$, mult., *J* (Hz)	LR C-H correlations [c]	NOE correlations [d]
Ac	1	171.1 s		Ac-2, [1]Aib-NH,4	
	2	23.1 q	1.92 s		[1]Aib-NH,3,4
[1]Aib	1	175.7 [e] s		[2]Ala-NH, [1]Aib-NH,3,4	
	2	55.8 s		[1]Aib-NH,3,4	
	3	26.2 [h] q	1.35 s	[1]Aib-NH	1Aib-NH
	4	24.2 q	1.32 s	[1]Aib-3	[1]Aib-NH
	NH		8.55 s		Ac-2, [1]Aib-3,4
[2]Ala	1	174.5 s	-	[3]Ala-NH, [2]Ala-2,3	
	2	50.9 d	4.01 m	[2]Ala-NH,3	[2]Ala-NH,3
	3	16.8 q	1.31 d	[2]Ala-NH,2	[2]Ala-NH,2
	NH		8.24 d (5.5)		[3]Ala-NH, [2]Ala-2,3
[3]Ala	1	174.5 s	-	[4]Aib-NH, [3]Ala-2,3	
	2	51.0 d	4.01 m	[3]Ala-NH,3	[3]Ala-NH,3, [4]Aib-NH
	3	16.2 q	1.33 d	[2]Ala-NH,2	[3]Ala-NH,2
	NH		7.68 d		[2]Ala-NH, [3]Ala-2,3
[4]Aib	1	175.0 s		[5]Iva-NH, [4]Aib-3	
	2	56.0 [g] s		[4]Aib-NH,3,4	
	3	26.2 [h] q	1.41 [i] s	[4]Aib-NH,4	[4]Aib-NH
	4	23.3 [j] q	1.35 [k] s	[4]Aib-NH,3	[4]Aib-NH
	NH		7.86 s		[4]Aib-2,3, [3]Ala-2, [5]Iva-NH
[5]Iva	1	176.4 s		[6]Gln-NH, [5]Iva-3,NH	
	2	58.6 s		[5]Iva-NH,3a,3b,4,5	
	3a	25.7 t	2.20 m	[5]Iva-4	[5]Iva-4,3b
	3b		1.62 m		[5]Iva-4,3a,5
	4	7.4 q	0.73 t	[5]Iva-3a,3b	[5]Iva-NH,3a,3b
	5	22.5 q	1.35 s	[5]Iva-NH,3a,3b	[5]Iva-NH,3b
	NH		7.48 s		[5]Iva-4,5, [4]Aib-NH
[6]Gln	1	173.8 s		[7]Aib-NH, [6]Gln-2	
	2	56.0 d	3.78 m	[6]Gln-NH,3,4a,4b	[7]Aib-NH, [6]Gln-NH,3,4a,4b
	3	26.3 t	1.97 m	[6]Gln-2,4a,4b,NH	[6]Gln-2,4a,4b,NH
	4a	31.6 [f] t	2.15 m	[6]Gln-2, NH_2 b	[6]Gln-2,3,4b,NH_2a
	4b		2.25 m		[6]Gln-2,3,4a
	5	173.7 s		[6]Gln-3,4a,4b,NH_2a,NH_2b	
	NH_2a		7.15 s		[6]Gln-4a,NH_2b
	NH_2b		6.75 s		[6]Gln-NH_2a
	NH		7.73 m		[6]Gln-2,3
[7]Aib	1	175.8 [e] s		[8]Aib-NH,[7]Aib-NH,3,4	
	2	56.1 [g] s		[7]Aib-NH,3,4	
	3	26.5 [h] q	1.41 [i] s	[7]Aib-NH,4	[7]Aib-NH
	4	23.3 [j] q	1.35 [k] s	[7]Aib-3	[7]Aib-NH
	NH		7.85 s		[6]Gln-2, [7]Aib-3,4,[8]Aib-NH
[8]Aib	1	175.9 [e] s		[9]Aib-NH, [8]Aib-3,4	
	2	56.1 [g] s		[8]Aib-NH,3,4	
	3	26.5 [h] q	1.42 [i] s	[8]Aib-NH,4	[8]Aib-NH
	4	22.7 [j] q	1.37 [k] s	[8]Aib-3	[8]Aib-NH
	NH		7.54 s		[8]Aib-3,4, [7]Aib-NH, [9]Aib-NH

Table S10. *Cont.*

^{9}Aib	1	176.4 s		^{10}Ser-NH, ^{9}Aib-3,4	
	2	56.1 [g] s		^{9}Aib-NH,3,4	
	3	26.4 [h] q	1.46 s	^{9}Aib-NH,4	
	4	23.1 [j] q	1.42 s	^{9}Aib-3	^{9}Aib-NH
	NH		7.76 s		^{9}Aib-3,4, ^{8}Aib-NH
^{10}Ser	1	170.6 s		^{11}Leu-NH, ^{10}Ser-2,3a,3b	-
	2	58.9 d	4.01 m	^{10}Ser-NH	^{10}Ser-NH
	3a	61.2 t	3.74 m	^{10}Ser-NH,2	
	3b		3.78 m		^{10}Ser-NH
	OH		-	-	
	NH		7.73 m		^{10}Ser-2,3b
^{11}Leu	1	173.7 s	-	^{12}Aib-NH, ^{11}Leu-2	
	2	51.5 d	4.26 m	^{11}Leu-NH,3b	^{11}Leu-NH,5,3a,3b, ^{13}Pro-5a
	3a	39.2 t	1.56 m	^{11}Leu-2,5,6	^{11}Leu-2,3b,5
	3b		1.70 m		^{11}Leu-NH,2,3a,5
	4	24.0 d	1.73 m	^{11}Leu-5,6	^{11}Leu-NH,6
	5	20.5 q	0.75 d	^{11}Leu-6,3b	^{11}Leu-2,3b,3a
	6	22.9 q	0.81 d	^{11}Leu-5,3b	^{11}Leu-4
	NH		7.58 d (7.5)		^{11}Leu-2,3b,4, ^{12}Aib-NH
^{12}Aib	1	172.9 s		^{12}Aib-NH,3,4	
	2	56.2 [g] s		^{12}Aib-NH,3,4	
	3	25.9 q	1.39 s	^{12}Aib-4	^{12}Aib-NH
	4	22.9 [j] q	1.48 s	^{12}Aib-NH,3	^{12}Aib-NH, ^{13}Pro-5b
	NH		7.88 s		^{11}Leu-NH, ^{12}Aib-3,4
^{13}Pro	1	173.3 s	-	^{14}Leu-NH, ^{13}Pro-2,3a	-
	2	63.0 d	4.21 t (8.0)	^{13}Pro-3a,4	^{13}Pro-4,3b
	3a	28.7 t	1.59 m	^{13}Pro-4,2,5b,5a	^{13}Pro-3b
	3b		2.23 m		^{13}Pro-3a,2,4
	4	25.9 t	1.86 m	^{13}Pro-2,5a,5b	^{13}Pro-2,3b,5a,5b
	5a	48.7 t	3.44 m	^{13}Pro-3b,3a	^{13}Pro-2,4,5b,^{11}Leu-2
	5b		3.68 m		^{13}Pro-4,5a, ^{12}Aib-3
^{14}Leu	1	173.7 s	-	^{15}Aib-NH, ^{14}Leu-2	-
	2	53.5 d	3.93 m	^{14}Leu-NH	^{14}Leu-4,3a,6,NH, ^{15}Aib-NH
	3a	38.8 t	1.51 m	^{14}Leu-2,4,5,6	^{14}Leu-2,3b,6
	3b		1.78 m		^{14}Leu-3a,6,NH
	4	24.8 d	1.68 m	^{14}Leu-5,6,2	^{14}Leu-2,5,NH
	5	22.9 q	0.91 d (6.0)	^{14}Leu-6,4	^{14}Leu-4
	6	21.1 q	0.82 d	^{14}Leu-5,3b,4	^{14}Leu-2,3a,3b
	NH		7.71 m		^{14}Leu-2,3b,4
^{15}Aib	1	175.3 s		^{16}Ile-2,NH, ^{15}Aib-NH,3,4	
	2	56.3 s		^{15}Aib-NH,3,4	
	3	26.5 [h] q	1.43 [i] s	^{15}Aib-4	^{15}Aib-NH
	4	23.2 [j] q	1.33[k] s	^{15}Aib-NH,3	^{15}Aib-NH
	NH		7.62 s		^{15}Aib-3,4, ^{16}Ile-NH, ^{14}Leu-2

Table S10. *Cont.*

^{16}Ile	1	172.0 s	-	^{17}Gln-NH, ^{16}Ile-2,3	-
	2	58.9 d	3.91 t (6.8)	^{16}Ile-NH,6,3,4a,4b	^{16}Ile-NH,5,6,4a,4b,3, ^{17}Gln-NH
	3	35.7 d	1.87 m	^{16}Ile-2,4a,4b,5,6,NH	^{16}Ile-NH,2,6
	4a	25.1 t	1.20 m	^{16}Ile-2,5,6	^{16}Ile-NH,4b,2
	4b		1.47 m		^{16}Ile-NH,2,4a
	5	11.4 q	0.81 t	^{16}Ile-4a,4b	^{16}Ile-2
	6	15.7 q	0.84 d (6.5)	^{16}Ile-4a,4b,2	^{16}Ile-2,3
	NH		6.95 d (6.5)		^{16}Ile-2,3,4a,4b, ^{15}Aib-NH
^{17}Gln	1	171.5 s		^{18}Gln-NH, ^{17}Gln-2	
	2	53.8 d	4.03 m	^{17}Gln-NH,4a,4b	^{17}Gln-NH,3,4a,4b
	3	27.2 t	1.84 m	^{17}Gln-2,4a,4b,NH	^{17}Gln-NH,2
	4a	31.7 ^{f} t	2.08 m	^{17}Gln-NH$_2$a,2,3	^{17}Gln-NH,2,NH$_2$b
	4b		2.18 m		^{17}Gln-2,NH$_2$b
	5	173.6 s		^{17}Gln-3,4a,4b,NH$_2$a,NH$_2$b	
	NH$_2$a		6.71 s		^{17}Gln-NH$_2$b
	NH$_2$b		7.15 s		^{17}Gln-NH$_2$a,4a,4b
	NH		7.70 m		^{17}Gln-2,3,4a, ^{16}Ile-2
^{18}Gln	1	170.9 s		^{19}Pheol-NH, ^{18}Gln-2,3a,3b	
	2	53.1 d	4.05 m	^{18}Gln-NH,3a,4a,4b	^{19}Pheol-NH, ^{18}Gln-NH,3a,3b,4b
	3a	27.7 t	1.81 m	^{18}Gln-2,4a,4b,NH	^{18}Gln-2,3b,4a
	3b		1.70 m		^{18}Gln-NH,2,3a,4b
	4a	31.8 ^{f} t	2.04 m	^{18}Gln-NH$_2$a,2,3a,3b	^{18}Gln-3a
	4b		1.95 m		^{18}Gln-NH, NH$_2$b,2,3b
	5	173.6 s		^{18}Gln-NH2 a, NH$_2$b,3a,3b,4a,4b	
	NH$_2$a		6.64 s		^{18}Gln-NH$_2$b
	NH$_2$b		7.10 s		^{18}Gln-NH$_2$a,4b
	NH		7.47 d		^{18}Gln-2,3b,4b
^{19}Pheol	1a	62.7 t	3.29 m	^{19}Pheol-2,3a,3b	^{19}Pheol-NH,2,5
	1b		3.32 m		^{19}Pheol-NH,2
	2	52.6 d	3.86 m	^{19}Pheol-1a,1b,3a,3b,NH	^{19}Pheol-NH,1a,1b,3a,3b,5
	3a	36.7 t	2.61 dd (13.8, 8.3)	^{19}Pheol-1a,1b,2,5	^{19}Pheol-NH, 2
	3b		2.83 dd (13.5, 5.5)		^{19}Pheol-2
	4	139.2 s		^{19}Pheol-2,3a,3b,5,7	
	5	129.4 d	7.20 m	^{19}Pheol-3a,3b,6,7	^{19}Pheol-1a,2,7
	6	128.2 d	7.19 m	^{19}Pheol-5,7	
	7	126.0 d	7.12 m	^{19}Pheol-5	^{19}Pheol-5
	OH				
	NH		7.27 d (8.5)		^{18}Gln-2, ^{19}Pheol-1a,1b,2,3a

[a] Carried out on an AVANCE-400, Bruker instrument; [b] Multiplicity and assignment are from HSQC experiment; [c] Values determined from HMBC experiment; $^{n}J_{CH}$=8 Hz, recycle time 1 s; [d] Selected NOE's from a ROESY experiment; [e,f,g,h,i,j,k] These signals may interchange.

Table S11. NMR data for TA.VII [a] (**11**) in DMSO-d_6.

Position		δ_C, mult. [b]	δ_H, mult., *J* (Hz)	LR C-H correlations [c]	NOE correlations [d]
Ac	1	171.0 s		Ac-2, 1Aib-NH	
	2	23.0 q	1.92 s		1Aib-NH
1Aib	1	175.9 [e] s		2Ala-NH, 1Aib-NH,3,4	
	2	55.8 s		1Aib-NH,3,4	
	3	26.5 [h] q	1.34 s	1Aib-NH	1Aib-NH
	4	24.2 q	1.32 s	1Aib-3	1Aib-NH
	NH		8.55 s		Ac-2, 1Aib-3,4
2Ala	1	174.5 s		3Ala-NH, 2Ala-2,3	
	2	50.9 d	3.99 m	2Ala-NH,3	2Ala-NH,3
	3	16.8 q	1.30 d	2Ala-NH,2	2Ala-NH,2
	NH		8.24 d (5.6)		2Ala-2,3, 3Ala-NH
3Ala	1	174.5 s		4Aib-NH, 3Ala-2,3	
	2	51.0 d	4.01 m	3Ala-NH,3	3Ala-NH,3, 4Aib-NH
	3	16.2 q	1.33 d (7.5)	2Ala-NH,2	3Ala-NH,2
	NH		7.68 d		4Aib-NH, 3Ala-2,3, 2Ala-NH
4Aib	1	175.0 s		5Iva-NH, 4Aib-3	
	2	56.0 [i] s		4Aib-NH,3,4	
	3	26.5 [h] q	1.43 [j] s	4Aib-NH,4	4Aib-NH
	4	23.0 [g] q	1.34 [k] s	4Aib-NH,3	4Aib-NH
	NH		7.86 s		4Aib-2,3, 3Ala-NH,2
5Iva	1	176.4 s		6Gln-NH,2, 5Iva-3a,NH	
	2	58.6 s		5Iva-NH,3a,3b,4,5	
	3a	25.6 t	2.20 m	5Iva-4	5Iva-NH,4,3b
	3b		1.62 m		5Iva-4,3a
	4	7.4 q	0.73 t (7.2)	5Iva-3a,3b	5Iva-NH,3a,3b
	5	22.7 q	1.35 s	5Iva-NH,3a,3b	5Iva-NH
	NH		7.46 s		5Iva-4,5,3a
6Gln	1	173.8 s		7Aib-NH, 6Gln-2	
	2	56.0 d	3.77 m	6Gln-NH,3,4a,4b	7Aib-NH, 6Gln-NH,3,4a,4b
	3	26.2 t	1.97 m	6Gln-2,4a,4b,NH	6Gln-2,4a,4b,NH, 7Aib-NH
	4a	31.6 [f] t	2.14 m	6Gln-2,3,NH_2b	6Gln-NH_2a,2,3,4b
	4b		2.23 m		6Gln-NH_2a,2,3,4a
	5	173.6 s		6Gln-3,4a,4b,NH_2a,NH_2b	
	NH_2a		7.16 s		6Gln-4a,NH_2b,4a,4b
	NH_2b		6.75 s		6Gln-NH_2a
	NH		7.72 m		6Gln-2,3
7Aib	1	175.8 [e] s		8Aib-NH, 7Aib-NH,3,4	
	2	56.1 [i] s		7Aib-NH,3,4	
	3	26.5 [h] q	1.42 [j] s	7Aib-NH,4	7Aib-NH
	4	23.3 [g] q	1.34 [k] s	7Aib-NH,3	7Aib-NH
	NH		7.85 s		6Gln-2,3, 7Aib-3,4
8Aib	1	175.7 [e] s		9Aib-NH, 8Aib-3,4	
	2	55.9 [i] s		8Aib-NH,3,4	
	3	26.3 [h] q	1.41 [j] s	8Aib-NH,4	8Aib-NH
	4	22.9 [g] q	1.37 [k] s	8Aib-3	8Aib-NH
	NH		7.54 s		8Aib-3,4

Table S11. *Cont.*

^{9}Aib	1	176.5 s		^{10}Ser-NH, ^{9}Aib-3,4	
	2	56.1 [i] s		^{9}Aib-NH,3,4	
	3	26.6 q	1.45 s	^{9}Aib-NH,4	^{9}Aib-NH
	4	22.9 [g] q	1.41 s	^{9}Aib-3	^{9}Aib-NH
	NH		7.76 s		^{9}Aib-3,4
^{10}Ser	1	170.6 s		^{11}Leu-NH, ^{10}Ser-2,3a	
	2	58.9 d	4.02 m	^{10}Ser-NH	^{10}Ser-NH,3a,3b
	3a	61.1 t	3.72 m	^{10}Ser-NH,2	^{10}Ser-NH,2
	3b		3.78 m		^{10}Ser-NH,2
	NH		7.72 m		^{10}Ser-2,3a,3b
^{11}Leu	1	173.5 s		^{12}Aib-NH, ^{11}Leu-2	
	2	51.3 d	4.27 m	^{11}Leu-NH,3b	^{12}Aib-NH, ^{11}Leu-NH,5,3a,3b,4, ^{14}Val-4
	3a	39.8 t	1.53 m	^{11}Leu-2,5,6	^{11}Leu-2,5
	3b		1.68 m		^{11}Leu-NH,2,5,6
	4	24.2 d	1.68 m	^{11}Leu-5,6	^{11}Leu-NH,2,5,6
	5	20.8 q	0.75 d	^{11}Leu-6,4	^{11}Leu-2,3a,3b
	6	23.2 q	0.82 d	^{11}Leu-5,3a	^{11}Leu-3b
	NH		7.60 m		^{11}Leu-2,3b,4
^{12}Aib	1	172.7 s		^{12}Aib-NH,3,4	
	2	56.2 [i] s		^{12}Aib-NH,3,4	
	3	26.0 [h] q	1.38 s		^{12}Aib-NH
	4	23.0 [g] q	1.48 s	^{12}Aib-NH,3	^{12}Aib-NH, ^{13}Pro-5b,2
	NH		7.93 s		^{11}Leu-2, ^{12}Aib-3,4
^{13}Pro	1	173.7 s		^{14}Val-NH, ^{13}Pro-2,3a	
	2	63.0 d	4.24 t (7.6)	^{13}Pro-3a,3b,4,5b	^{14}Val-NH, ^{13}Pro-4, 3a,3b, ^{12}Aib-3
	3a	28.8 t	1.65 m	^{13}Pro-2,4	^{13}Pro-2,3b, 5a,4
	3b		2.22 m		^{13}Pro-3a,2,4
	4	26.0 t	1.86 m	^{13}Pro-2,3a,5a,5b	^{13}Pro-2,3a,3b,5a,5b
	5a	48.4 t	3.52 m	^{13}Pro-3a,3b,2,4	^{13}Pro-3a,4,5b
	5b		3.67 m		^{13}Pro-4,5a, ^{12}Aib-3
^{14}Val	1	172.6 s		^{15}Aib-NH, ^{14}Val-2	
	2	60.9 d	3.78 m	^{14}Val-3,4,5,NH	^{14}Val,5,3,NH, ^{15}Aib-NH
	3	28.8 d	2.21 m	^{14}Val-2,4,5,NH	^{14}Val-2,4,5,NH
	4	19.1 q	0.93 d (6.4)	^{14}Val-5,2,3	^{14}Val-NH,3, 11Leu-2
	5	19.1 q	0.87 d (6.8)	^{14}Val-4,2,3	^{14}Val-2,3
	NH		7.59 d		^{14}Val-2,3,4, ^{13}Pro-2
^{15}Aib	1	175.6 s		^{16}Ile-NH,2, ^{1}5Aib-NH,3,4	
	2	56.2 s		^{15}Aib-NH,3,4	
	3	26.1 [h] q	1.41 [j] s		^{15}Aib-NH
	4	23.2 [g] q	1. 38 [k] s	^{15}Aib-NH,3	^{15}Aib-NH
	NH		7.43 s		^{15}Aib-3,4, ^{14}Val-2

Table S11. *Cont.*

^{16}Ile	1	172.3 s		^{17}Gln-NH, ^{16}Ile-2	
	2	59.5 d	3.85 m	^{16}Ile-NH,6,4a,4b,3	^{16}Ile-NH,5,4a,3, ^{17}Gln-NH
	3	35.5 d	1.87 m	^{16}Ile-2,4a,4b,5,6,NH	^{16}Ile-NH,2,6
	4a	25.4 t	1.20 m	^{16}Ile-2,3,5,6	^{16}Ile-2,4b,NH
	4b		1.50 m		^{16}Ile-4a,5
	5	11.4 q	0.80 t (7.2)	^{16}Ile-4a,4b	^{16}Ile-4b,2,NH
	6	15.7 q	0.84 d	^{16}Ile-4a,4b,2	^{16}Ile-3
	NH		7.21 m		^{16}Ile-2,3,4a,5
^{17}Gln	1	171.8 s		^{18}Gln-NH, ^{17}Gln-2	
	2	54.3 d	3.97 m	^{17}Gln-NH,4a,4b,3	^{17}Gln-NH,3,4a,4b,NH$_2$b
	3	26.8 t	1.93 m	^{17}Gln-2,4a,4b	^{17}Gln-NH,2
	4a	31.7 [f] t	2.13 m	^{17}Gln-NH$_2$a,2,3	^{17}Gln-NH,2,NH$_2$b
	4b		2.23 m		^{17}Gln-2,NH$_2$b
	5	173.6 s		^{17}Gln-3,4a,4b,NH$_2$a,NH$_2$b	
	NH$_2$a		6.72 s		^{17}Gln-NH$_2$b
	NH$_2$b		7.16 s		^{17}Gln-NH$_2$a,4a,4b,2
	NH		7.70 m		^{17}Gln-2,3,4a, ^{16}Ile-2
^{18}Gln	1	171.0 s		^{19}Pheol-NH, ^{18}Gln-2,3a,3b	
	2	53.4 d	4.00 m	^{18}Gln-NH,3a,3b,4a,4b	^{19}Pheol-NH, ^{18}Gln-NH,3a,3b,4b
	3a	27.5	1.81 m	^{18}Gln-2,4a,4b,NH	^{18}Gln-2,3b,4a
	3b		1.70 m		^{18}Gln-2,3a,4a,NH
	4a	31.8 [f] t	2.05 m	^{18}Gln-NH$_2$a,2,3a,3b	^{18}Gln-3a,3b
	4b		1.97 m		^{18}Gln-NH,NH$_2$b,2
	5	173.6 s		^{18}Gln-NH$_2$a,NH$_2$b,3a,3b,4a,4b	
	NH$_2$a		6.61 s		^{18}Gln-NH$_2$b
	NH$_2$b		7.06 s		^{18}Gln-NH$_2$a,4b
	NH		7.44 d		^{19}Pheol-NH,^{18}Gln-2,3b,4b
^{19}Pheol	1a	62.8 t	3.30 m	^{19}Pheol-2,3a,3b	^{19}Pheol-NH
	1b		3.33 m		^{19}Pheol-NH
	2	52.6 d	3.85 m	^{19}Pheol-1a,1b,3a,3b,NH	^{19}Pheol-NH,3a,3b,5
	3a	36.7 t	2.62 dd (13.4, 12.0)	^{19}Pheol-1a,1b, 5	^{19}Pheol-NH,2
	3b		2.83 dd (13.4, 5.0)		^{19}Pheol-2
	4	139.3 s		^{19}Pheol-2,3a,3b,6	
	5	129.4 d	7.22 m	^{19}Pheol-3a,3b,5,7	^{19}Pheol-2,3b,7
	6	128.1 d	7.18 m	^{19}Pheol-6	
	7	126.0 d	7.11 m	^{19}Pheol-5	^{19}Pheol-5
	NH		7.18 m		^{18}Gln-NH,2, ^{19}Pheol-1a,1b,2,3a

[a] Carried out on an AVANCE-400 Bruker instrument; [b] Multiplicity and assignment are from HSQC experiment; [c] Values determined from HMBC experiment; $^{n}J_{CH}$ = 8 Hz, recycle time 1 s; [d] Selected NOE's from a ROESY experiment; [e,f,g,h,i,j,k] These signals may interchange.

Table S12. NMR data for TA.Vb [a] (**12**) in DMSO-d_6.

Position		δ_C, mult. [b]	δ_H, mult., *J* (Hz)	LR C-H correlations [c]	NOE correlations [d]
Ac	1	171.1 s		Ac-2, [1]Aib-NH	
	2	23.1 q	1.93 s		[1]Aib-NH,3,4
[1]Aib	1	175.9 [e] s		[2]Ala-NH, [1]Aib-NH,3,4	
	2	55.8 [g] s		[1]Aib-NH,3,4	
	3	26.5 [h] q	1.35 s	[1]Aib-NH	[1]Aib-NH
	4	24.2 [i] q	1.32 s	[1]Aib-3	[1]Aib-NH
	NH		8.58 s		Ac-2, [1]Aib-3,4
[2]Ala	1	174.6 s	-	[3]Ala-NH, 2Ala-2,3	
	2	50.9 d	4.00 m	[2]Ala-NH,3	[2]Ala-NH,3
	3	16.8 q	1.31 d	[2]Ala-NH,2	[2]Ala-NH,2
	NH		8.28 d (5.2)		[3]Ala-NH, [2]Ala-2,3
[3]Ala	1	174.7 s	-	[4]Aib-NH, [3]Ala-2,3	
	2	51.2 d	4.00 m	[3]Ala-NH,3	[3]Ala-NH
	3	16.1 q	1.33 d	[2]Ala-NH,2	[3]Ala-NH,2
	NH		7.71 m		[4]Aib-NH, [2]Ala-NH, [3]Ala-2,3
[4]Aib	1	175.1 s		[5]Aib-NH, [4]Aib-3,4	
	2	55.8 [g] s		[4]Aib-NH,3,4	
	3	26.5 [h]q	1.42 [j] s	[4]Aib-NH,4	[4]Aib-NH
	4	23.2 [i]q	1.39 [k] s	[4]Aib-NH,3	[4]Aib-NH
	NH		7.83 s		[4]Aib-4,3, [3]Ala-NH, [5]Aib-NH
[5]Aib	1	176.0 [e] s		[6]Gln-NH, [5]Aib-NH,3,4	
	2	56.0 [g] s		[5]Aib-NH,3,4	
	3	23.0 [h] q	1.44 s	[5]Aib-4,NH	[5]Aib-NH
	4	26.5 [i] q	1.34 s	[5]Aib-3	[5]Aib-NH
	NH		7.55 s		[5]Aib-3,4, [4]Aib-NH
[6]Gln	1	173.8 s		[7]Aib-NH, [6]Gln-2,3	
	2	56.0 d	3.77 m	[6]Gln-NH,3,4a,4b	[6]Gln-NH,3,4a,4b
	3	26.2 t	1.97 m	[6]Gln-2,4a,4b,NH	[6]Gln-2,NH
	4a	31.5 [f] t	2.15 m	[6]Gln-2,3,NH_2b	[6]Gln-2, 4b,NH_2a
	4b		2.23 m		[6]Gln-2,4a,NH_2a
	5	173.7 s		[6]Gln-3,4a,4b,NH_2a,NH_2b	
	NH_2a		7.16 s		[6]Gln-4a,4b,NH_2b
	NH_2b		6.76 s		[6]Gln-NH_2a
	NH		7.77 m		[6]Gln-2,3
[7]Aib	1	176.0 [e] s		[8]Aib-NH, [7]Aib-NH,3,4	
	2	56.0 [g] s		[7]Aib-NH,3,4	
	3	26.3 [h] q	1.41 [j] s	[7]Aib-NH,4	[7]Aib-NH
	4	23.0 [i] q	1.35 [k] s	[7]Aib-NH,3	[7]Aib-NH
	NH		7.86 s		[7]Aib-3,4, [8]Aib-NH
[8]Aib	1	175.7 s		[9]Aib-NH, [8]Aib-3,4	
	2	56.1 [g] s		[8]Aib-NH,3,4	
	3	26.5 [h] q	1.40 [j] s	[8]Aib-NH,4	[8]Aib-NH
	4	23.2 [i] q	1.37 [k] s	[8]Aib-3	[8]Aib-NH
	NH		7.59 s		[8]Aib-3,4, [7]Aib-NH

Table S12. *Cont.*

9Aib	1	176.4 s		10Ser-NH, 9Aib-3,4,NH	
	2	56.1 g s		9Aib-NH,3,4	
	3	26.6 q	1.48 s	9Aib-NH,4	9Aib-NH
	4	22.8 q	1.42 s	9Aib-3	9Aib-NH
	NH		7.79 s		9Aib-3,4
10Ser	1	170.6 s		11Leu-NH, 10Ser-2	-
	2	58.9 d	4.02 m	10Ser-NH	10Ser-NH,3a,3b, 11Leu-NH
	3a	61.2 t	3.74 m	10Ser-NH,2	10Ser-2
	3b		3.78 m		10Ser-2
	OH		4.80 t (6.2)	-	10Ser-NH
	NH		7.74 m		10Ser-2,OH
11Leu	1	173.6 s	-	12Aib-NH	
	2	51.6 d	4.28 t	11Leu-NH,4	11Leu-NH,5,3a,3b, 13Pro-5a
	3a	39.2 t	1.56 m	11Leu-2.5,6	11Leu-2,5,6,3b
	3b		1.70 m		11Leu-NH,2,3a,6
	4	24.0 d	1.73 m	11Leu-5,6	11Leu-5
	5	20.5 q	0.76 d	11Leu-6,3b	11Leu-2,3a,4
	6	23.0 q	0.82 d	11Leu-5,3b	11Leu-3a,3b
	NH		7.59 d		10Ser-2, 11Leu-2,3
12Aib	1	173.0 s		12Aib-NH,3,4	
	2	56.3 g s		12Aib-NH,3,4	
	3	26.5 h q	1.40 s	12Aib-NH,4	12Aib-NH, 13Pro-5b
	4	23.0 i q	1.46 s	12Aib-3	
	NH		7.92 s		12Aib-3
13Pro	1	173.3 s	-	14Leu-NH, 13Pro-2	-
	2	63.0 d	4.22 t (8.2)	13Pro-3a,4,5b	14Leu-5, 13Pro-4,3b
	3a	28.7 t	1.60 m	13Pro-2,5b,4	13Pro-3b,5a
	3b		2.24 m		13Pro-3a,2, 4
	4	26.1 t	1.86 m	13Pro-2,5b,5a,3a	13Pro-2,3b,5a,5b
	5a	48.7 t	3.44 m	13Pro-3b	13Pro-2,3a,4, 11Leu-2
	5b		3.70 m		13Pro-4, 12Aib-3
14Leu	1	173.7 s	-	15Aib-NH	-
	2	53.6 d	3.95 m	14Leu-NH	14Leu-4,3a,3b,5,6 ,NH
	3a	38.7 t	1.52 m	14Leu-4,5,6	14Leu-2,3b,5,6
	3b		1.78 m		14Leu-2,3a,5,6,NH,4
	4	24.9 d	1.68 m	14Leu-5,6	14Leu-2,5,6,NH,3b
	5	23.0 q	0.92 d (6.0)	14Leu-6,4,3a	14Leu-2,4,3a,3b, 13Pro-2
	6	21.1 q	0.82 d	14Leu-5,4	14Leu-2,3a,3b,4
	NH		7.72 m		14Leu-2, 3b,4
15Aib	1	175.4 s		16Ile-NH, 15Aib-NH,3,4	
	2	56.2 g s		15Aib-NH,3,4	
	3	26.6 h q	1.41 s	15Aib-4	15Aib-NH
	4	24.2 i q	1.38 s	15Aib-NH,3	15Aib-NH
	NH		7.63 s		15Aib-3,4, 16Ile-NH

Table S12. *Cont.*

^{16}Ile	1	172.0 s	-	^{17}Gln-NH, ^{16}Il-2	-
	2	59.0 d	3.91 m	^{16}Ile-NH, ^{16}Ile-6,4b,3	^{16}Ile-NH,6,4b,3, ^{17}Gln-NH
	3	35.6 d	1.88 m	^{16}Ile-2,4a,4b,5,6,NH	^{16}Ile-NH,2,5,6
	4a	25.0 t	1.20 m	^{16}Ile-2,5,6	^{16}Ile-4b
	4b		1.47 m		^{16}Ile-2,4a,5
	5	11.5 q	0.82 t	^{16}Ile-4a,4b	^{16}Ile-4b,3
	6	15.7 q	0.86 d	^{16}Ile-4a,4b,2	^{16}Ile-2,3
	NH		6.97 d (6.4)		^{16}Ile-2,3, ^{15}Aib-NH,3,4
^{17}Gln	1	171.5 s		^{18}Gln-NH, ^{17}Gln-2	
	2	53.8 d	4.02 m	^{17}Gln-NH,4a,4b,3b	^{18}Gln-NH, ^{17}Gln-NH,3a,3b,4a
	3a	27.2 t	1.85 m	^{17}Gln-2,4a,4b	^{17}Gln-NH,2,4b
	3b		1.94 m		^{17}Gln-NH,2.4a
	4a	31.6 [f] t	2.08 m	^{17}Gln-2	^{17}Gln-2,3b
	4b		2.18 m		^{17}Gln-NH,3a
	5	173.7 s		^{17}Gln-4a,4b	
	NH_2a		6.72 s		^{18}Gln-NH_2b
	NH_2b		7.15 s		^{18}Gln-NH_2a,4b
	NH		7.71 m		^{17}Gln-2,3a,3b,4b, ^{16}Ile-2
^{18}Gln	1	170.9 s		^{19}Pheol-NH, ^{18}Gln-2	
	2	53.1 d	4.05 m	^{18}Gln-NH,3a,3b,4a,4b	^{19}Pheol-NH, ^{18}Gln-NH,3a,3b,4b
	3a	27.7 t	1.83 m	^{18}Gln-2,4a,4b,NH	^{18}Gln-2,3b,4a
	3b		1.70 m		^{18}Gln-NH,2,3a,4a
	4a	31.7 [f] t	2.05 m	^{18}Gln-NH_2a,2,3a,3b	^{18}Gln-3a,3b
	4b		1.97 m		^{18}Gln-NH,NH_2b,2
	5	173.7 s		^{18}Gln-NH_2a,NH_2b,3a, 3b,4a,4b	
	NH_2a		6.65 s		^{18}Gln-NH_2b
	NH_2b		7.11 s		^{18}Gln-NH_2a,4b
	NH		7.48 d (7.6)		^{18}Gln-2,3b,4b
^{19}Pheol	1a	62.7 t	3.30 m	^{19}Pheol-2,3a,3b	^{19}Pheol-2,3a,3b,5
	1b		3.32 m		^{19}Pheol-2,3a,3b
	2	52.6 d	3.86 m	^{19}Pheol-1a,1b,3a,3b,NH	^{19}Pheol-NH,1a,1b,3a,3b,5
	3a	36.7 t	2.62 dd (13.4, 8.3)	^{19}Pheol-1a,1b,2,5	^{19}Pheol-NH,1a,1b,2,5
	3b		2.84 dd (13.6, 5.2)		^{19}Pheol-1a,1b,2,5
	4	139.2 s		^{19}Pheol-2,3a,3b,5,7	
	5	129.4 d	7.21 m	^{19}Pheol-3a,3b,6,7	^{19}Pheol-1a,2,3a,3b,7
	6	128.2 d	7.19 m	^{19}Pheol-5,7	
	7	126.0 d	7.13 m	^{19}Pheol-5,6	^{19}Pheol-5
	OH		4.71 t (5.6)		^{19}Pheol-NH
	NH		7.28 d (8.8)		^{18}Gln-2, ^{19}Pheol-2,3a,OH

[a] Carried out on an AVANCE-400 Bruker instrument; [b] Multiplicity and assignment are from HSQC experiment; [c] Values determined from HMBC experiment; $^{n}J_{CH}$ = 8 Hz, recycle time 1 s; [d] Selected NOE's from a ROESY experiment; [e,f,g,h,i,j,k] These signals may interchange.

Reference

1. Bodo, B.; Rebuffat, S.; El Hajii, M.; Davoust, D. Structure of Trichorzianine A IIIC, an antifungal peptide from *Trichoderma harzianum*. *J. Am. Chem. Soc.* **1985**, *107*, 6011–6017.
2. El Hajii, M.; Rebuffat, S.; Lecommandeur, D.; Bodo, B. Isolation and sequence determination of trichorzianines A antifungal peptides from *Trichoderma harzianum*. *Int. J. Peptide Prot. Res.* **1987**, *29*, 207–215.
3. Rebuffat, S.; El Hajii, M.; Hennig, P.; Davoust, D.; Bodo, B. Isolation, sequence and conformation of seven trichorzianines B from *Trichoderma harzianum*. *Int. J. Peptide. Prot. Res.* **1989**, *34*, 200–210.

© 2013 by the authors; licensee MDPI, Basel, Switzerland. This article is an open access article distributed under the terms and conditions of the Creative Commons Attribution license (http://creativecommons.org/licenses/by/3.0/).
